# Metal-Free Cascade Formation of C–C and C–N Bond for the Construction of 3-Cyano-2-Pyridones with Insecticidal Properties

**DOI:** 10.3390/molecules29122792

**Published:** 2024-06-12

**Authors:** Yao Tang, Nvjiang Wu, Junyu Xu, Xiaopo Zhang, Youbin Li, Xuesong Wang

**Affiliations:** Engineering Research Center of Tropical Medicine Innovation and Transformation of Ministry of Education, International Joint Research Center of Human-Machine Intelligent Collaborative for Tumor Precision Diagnosis and Treatment of Hainan Province, Hainan Key Laboratory for Research and Development of Tropical Herbs, Haikou Key Laboratory of Li Nationality Medicine, School of Pharmacy, Academy of Medical Sciences, Hainan Medical University, Haikou 571199, China; ty2024abcd@sina.com (Y.T.); healthyhu@163.com (N.W.); xujy201309@hainmc.edu.cn (J.X.)

**Keywords:** 3-cyano-2-pyridone, insecticidal, C–C and C–N bond, construction, metal-free, cascade

## Abstract

A straightforward and efficient methodology has been developed for the synthesis of 3-cyano-2-pyridones via the C–C and C–N bond formation processes. A total of 51 diverse 3-cyano-2-pyridone derivatives were obtained in moderate to excellent yields. This reaction featured advantages such as a metal-free process, wide functional group tolerance, simple operation, and mild conditions. A plausible mechanism for the reaction was proposed. 3-cyano-2-pyridones as ricinine analogues for insecticidal properties were evaluated, and the compound **3ci** (LC_50_ = 2.206 mg/mL) showed the best insecticidal property.

## 1. Introduction

**Figure 1:**
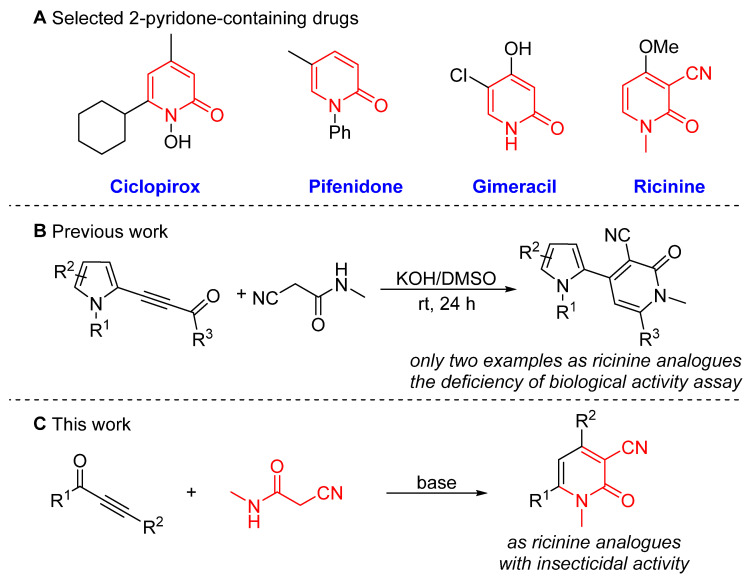
(**A**) Selected 2-pyridone-containing drugs. (**B**) Previous report of the synthesis of 2-pyridones. (**C**) Our synthesis route to 3-cyano-2-pyridones.

2-pyridone is considered a privileged heteroaromatic skeleton endowed with pharmaceutical properties and biological activities [1,2,3,4,5,6,7,8,9,10]. They are also widely applicable in organic synthesis, materials science, and polymer chemistry, which are used as valuable building blocks for the synthesis of nitrogen-containing heterocycles like pyridines, quinolizidines, *β*-lactams, piperidines, and indolizidine alkaloids [11,12,13,14,15,16,17,18,19,20,21,22,23,24]. Owing to their multifarious and prominent properties, 2-pyridones often display promising therapeutical areas such as antifungal, anti-HIV, antibacterial, antiepileptic, anti-inflammatory, and antitumoral activities [25,26,27,28,29,30,31,32,33,34,35,36,37]. As shown in Figure 1A, very typical commercial drugs based on the 2-pyridone scaffold include ciclopirox [38], pifenidone [39], gimeracil [40], and ricinine [41,42,43]. Due to their importance and utility in chemical and pharmaceutical applications, extensive effort has been devoted to the development of efficient methods for the synthesis of diversely functionalized 2-pyridones.

Typically, transition metals such as ruthenium, nickel, and rhodium have been exploited in the cycloaddition of 1,6-diynes with isocyanates towards 2-pyridones [44,45,46,47]. Complementarily, several other methods involving FeCl_3_-catalyzed intramolecular cascade reactions of acetoacetanilide with 3-formyl chromone [48], Ni-catalyzed reactions of alkynes via azazirconacycles [49], gold-catalyzed cycloisomerization of N-alkenyl alkynylamides [50], and indium-catalyzed domino reactions of 3-formylchromones with N-methyl-1-(methylthio)-2-nitroethenamine [51] have also been developed. However, the cytotoxicity of trace transition metals has limited their utilization in the fields of pharmaceutical application. Moreover, a literature survey implied that other existing approaches to 2-pyridones focus on ring-closing metathesis of *α*-amino acrylamides [52], *t*-BuOK-mediated condensation of enones with cyanoacetamides [53], CsF-catalyzed reaction of *α,β*-unsaturated diester chromones with aromatic amines [54], tandem Blaise reaction of nitriles with propiolates [55], heteropolyacid-catalyzed reaction of amines and acetylenic esters [56], intramolecular ketene trapping of enamine-dioxinones [57], nucleophilic addition of malonic esters with alkynyl imines [58], addition reaction of 2-(phenylsulfinyl)acetamide and α,β-unsaturated ketones [59]. Nevertheless, those reported methods for the synthesis of 2-pyridones suffer from considerable disadvantages, like expensive catalysts [44,45,46,47] and contamination of heavy metals [56], which have limited their potential application in the life science and pharmaceutical industries. Consequently, the development of efficient and environmentally benign procedures for the synthesis of 2-pyridones continues to be of great significance.

Ricinine [60] has been reported to exhibit pharmacological activities such as antifertility, antiimplantation, antinociceptive, anticancer, antidiabetic, and insecticidal activities (Figure 1A). On the other hand, ynones have emerged as valuable building blocks for the construction of complex heterocyclic rings, such as indole, pyrrole, pyridine, triazole, quinoline, thiazole, pyrimidine, and so on [61,62,63,64,65,66,67,68,69,70,71,72,73]. Recently, Trofimov and co-workers [74] reported the condensation of acylethynylpyrroles and 2-cyano-N-methylacetamide to form N-methyl-3-cyano-2-pyridones (Figure 1B). However, considerable disadvantages like the relatively poor functional scopes (only 2 examples of N-methyl-3-cyano-2-pyridone as ricinine analogues) and the deficiency of biological activity assays still exist in this process. Inspired by the aforementioned works and previous reports [60,61,62,63,64,65,66,67,68,69,70,71,72,73,74,75,76], our primary aim is to develop an easy and efficacious synthetic strategy for the expeditious assembly of 3-cyano-2-pyridones from the cycloaddition of ynones and 2-cyanoacetamide (Figure 1C). Simultaneously, we strive to identify 3-cyano-2-pyridones that serve as ricinine analogues and display potent insecticidal properties.

## 2. Results and Discussion

### 2.1. Optimization of the Reaction Conditions

In an initial effort, we began our investigation with the cascade cycloaddition of 1,3-diphenylprop-2-yn-1-one **1aa** and 2-cyano-*N*-methylacetamide **2aa** in 1,4-dioxane in the presence of K_2_CO_3_ as a base at 100 °C for 8 h (Table 1). To our delight, the preliminary result showed the desired product **3aa** was obtained with an 81% yield (entry 1). The yield was decreased when the transition-metal-free reaction was conducted with Na_2_CO_3_ or Cs_2_CO_3_ instead of K_2_CO_3_ (entries 2–3). Moreover, various kinds of bases were screened, whereas no reaction or trace transformation was observed, neither strong inorganic bases such as NaOH, NaH, EtONa, LiO*^t^*Bu, NaO*^t^*Bu, and KO*^t^*Bu (entries 4–9) nor organic bases including Et_3_N, DBU, and DABCO (entries 10–12) involving the transformation. Further investigation showed that this reaction was highly solvent-dependent (entries 13–19). When the reaction was carried out in DMSO, DMF, and NMP, an acceptable yield of **3aa** was detected (entries 13–15). However, other solvents, such as CH_3_CN, toluene, THF, and EtOH, afforded only a trace amount of the desired product **3aa** (entries 16–19). Finally, decreasing the amount of K_2_CO_3_ lowered the yield (entry 20), while no obvious deviation was observed when the loading of K_2_CO_3_ was increased (entries 21–22). On the basis of the above results, the optimal conditions for this transformation were obtained as follows: K_2_CO_3_ (1.2 equiv.) in 1,4-dioxane at 100 °C for 8 h.

### 2.2. Scope of the Reaction

With the optimum conditions in hand, the generality and scope of this metral-free cascade reaction of ynones and 2-cyanoacetamides were explored, and the results are shown in Table 2. Firstly, we studied the scope of alkynones with substituents in Ar–R^1^ (entries 2–20). The reaction shows preference for electron-rich alkynones (Me, OMe) (entries 2–6), which provided the corresponding products in relatively higher yields than their counterparts bearing electron-withdrawing groups (F, Cl, Br, I, CF_3_) (entries 7–20). Compared to the yield of **3ab** and the yield of **3ad** (87% vs. 73%), we found that the steric hindrance influenced this reaction. Particularly, a 2-naphthyl-substituted substrate could also be subjected to the reaction, and the corresponding product, **3au**, was obtained with a 90% yield. Moreover, both alkyl- and heterocyclyl involving-alkynones were also suitable candidates for this transformation, and the corresponding products, **3av**, **3aw**, and **3ax** were obtained in 83%, 76%, and 84% yields, respectively.

As for regioisomeric substrates, alkynones with substituents in Ar–R^2^ were also investigated (entries 25–34). Similar to alkynones with functional groups in Ar–R^1^, the cycloaddition reactions proceeded smoothly to give the corresponding 3-cyano-2-pyridones in satisfactory yields. Notably, both electronic effect (entries 25–29 vs. entries 30–34) and steric hindrance influence (**3bg** vs. **3bh**) existed in this reaction. As outlined in Table 2, electron-donating and *para*-substituted alkynones were good substrates, leading to the target products in moderate to excellent yields (entries 25–29). Interestingly, alkynones with cyclopropyl, *n*-hexyl, and 2-thienyl groups were also tolerated in this procedure and provided 3-cyano-2-pyridones in satisfactory yield (entries 35–37).

Additionally, alkynones with substituents in Ar–R^1^ and Ar–R^2^ were also evaluated under the standard reaction conditions. The corresponding 3-cyano-2-pyridones were obtained in moderate to excellent yields regardless of the electron-donating or electron-withdrawing groups at benzene rings (entries 38–45). Encouragingly, alkynones bearing two electron-rich functional groups provided excellent yields because of the strong electron-donating effect (entries 38–40). However, with *ortho*-functional group substrates, the yields of corresponding products decreased, maybe due to the steric hindrance (entries 41–42 and 44–45). Furthermore, alkynones possessing a naphthyl and cyclopropyl ring were carried out in this cascade reaction system and furnished the desired products **3ci**, **3cj**, and **3ck** in 88%, 80%, and 60% yields, respectively (entries 46–48). Finally, 2-cyanoacetamides with functional groups phenyl and benzyl were tested and provided the products in moderate yield (entries 49–50), while 2-cyanoacetamide involved in the transformation obtained the 3-cyano-2-pyridone in excellent yield (entry 51).

### 2.3. Gram-Scale of the Reaction

**Scheme 1:**
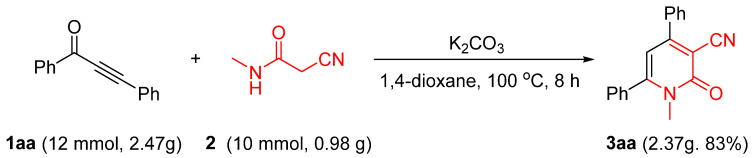
Gram-scale of **3aa**.

To demonstrate the reliability and practicality of this present synthetic protocol, a gram-scale preparation was performed using starting materials **1aa**, where the desired product, **3aa**, was isolated in an 83% yield (Scheme 1).

### 2.4. Plausible Mechanism of the Reaction

**Scheme 2:**
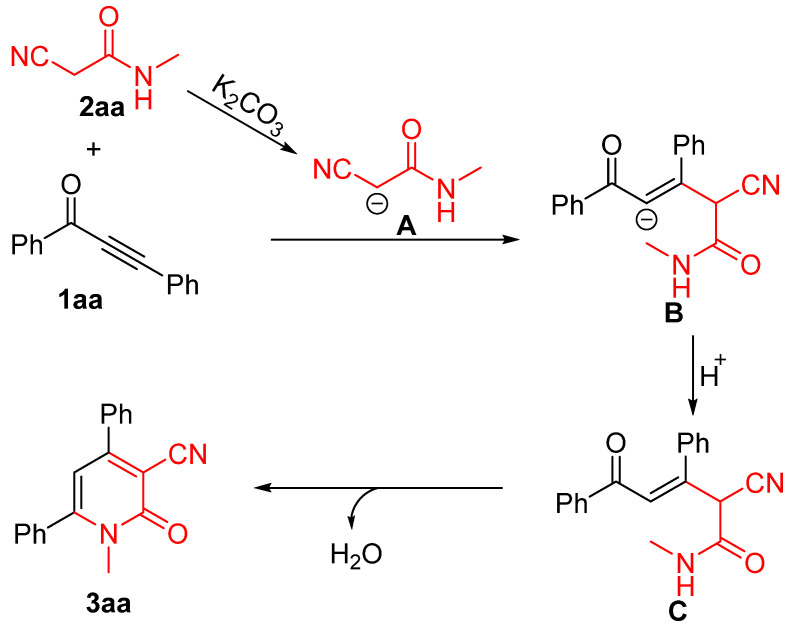
Plausible mechanism.

On the basis of the aforementioned observations and previous reports [74], the mechanistic pathways of this cascade methodology were proposed and presented in Scheme 2. Initially, 2-cyano-*N*-methylacetamide **2aa** undergoes a K_2_CO_3_-initiated deprotonation of the CH_2_ group to give carbanion **A**, and then the nucleophilic addition of carbanion **A** to the triple bond of ynone **1aa** produces carbanion **B**. Subsequently, the carbanion **B** is quenched by a proton of the medium to generate intermediate **C**. Finally, an intramolecular cycloaddition of the intermediate **C** results in the formation of the cyclization product **3aa**.

### 2.5. Study of the Insecticidal Properties of 3-Cyano-2-Pyridones

**Figure 2:**
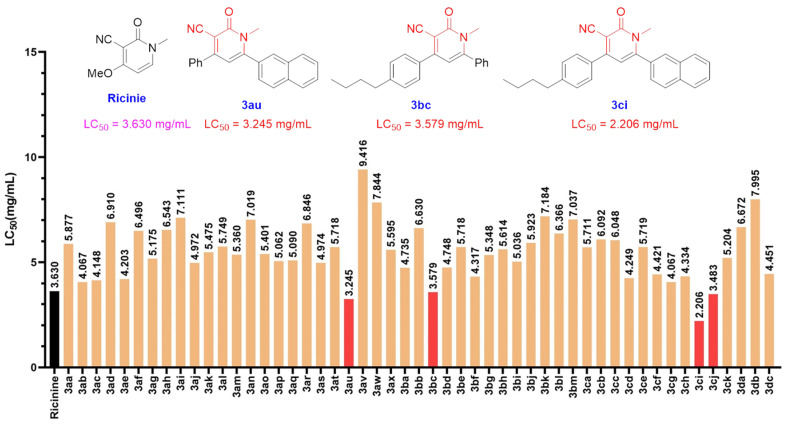
The insecticidal properties of 3-cyano-2-pyridones.

A literature survey implied that ricinine is well known as a compound with a high level of biological activity and has been proven valuable due to its insecticidal properties [77]. Based on the structure of ricinine, we have prepared several 3-cyano-2-pyridones with novel scaffolds as ricinine analogues to evaluate their insecticidal activity (see the experimental details in Supplementary Materials). Fortunately, in the trials of killing mealworm, we observed that the compounds **3au** (LC_50_ = 3.245 mg/mL) and **3bc** (LC_50_ = 3.579 mg/mL) exhibited powerful pesticidal activity using ricinine as a positive control. These results implied that 2-naphthyl and *para-n*-butyl benzene are good functional groups in the 3-cyano-2-pyridone framework as insecticides. Subsequently, the suitable functional candidate **3ci** (LC_50_ = 2.206 mg/mL) bearing 2-naphthyl and *para-n*-butyl benzene groups was obtained, and the insecticidal activity was evaluated. Upon comparing the LC_50_ values in Figure 2, we found that the compound **3ci** was the best candidate drug with insecticidal properties.

## 3. Experimental Section

### 3.1. General Methods and Materials for the Synthesis of 3-Cyano-2-Pyridones 3

Unless otherwise stated, all reagents were used directly without further purification. Silica gel was purchased from Qing Dao Hai Yang Chemical Industry Co. (Qinqdao, China). All melting points were determined on a Beijing Science Instrument Dianguang Instrument Factory XT4B (Beijing, China) melting point apparatus and uncorrected. ^1^H and ^13^C NMR spectra were measured on a 400 MHz JEOL JNM-ECZ400S/L1 spectrometer (Jeol Ltd., Tokyo, Japan), (^1^H 400 MHz, ^13^C 100 MHz), using CDCl_3_ as the solvent with tetramethylsilane (TMS) as the internal standard at room temperature. HRMS-ESI spectra were equipped with an ESI source and a TOF detector. PE is petroleum ether (60–90 °C).

A Typical Procedure for the Synthesis of 1-methyl-2-oxo-4,6-diphenyl-1,2-dihydropyridine-3-carbonitrile (**3aa**).

A suspension of 1,3-diphenylprop-2-yn-1-one **1aa** (0.6 mmol, 123.6 mg), 2-cyano-*N*-methylacetamide **2aa** (0.5 mmol, 49.0 mg), and K_2_CO_3_ (0.6 mmol, 82.8 mg) in 1,4-dioxane (2 mL) was stirred at 100 °C for 8 h. After **2aa** was exhausted completely (monitored by TLC), it was cooled down to room temperature and saturated aqueous brine (20 mL) was added. The mixture was stirred for 10 min and then extracted by EtOAc (3 × 10 mL). The combined organic layers were dried over Na_2_SO_4_. Removal of the solvent gave a residue, which was purified by column chromatography (silica gel, PE/EtOAc = 3/1 *v*/*v*) to afford **3aa** as a yellow solid, 116 mg, 81% yield; mp 148–151 °C. ^1^H NMR (400 MHz, chloroform-*d*) δ 7.63 (d, *J* = 3.96 Hz, 2H), 7.53–7.47 (m, 6H), 7.40 (d, *J* = 3.88 Hz, 2H), 6.32 (s, 1H), 3.47 (s, 3H). ^13^C NMR (100 MHz, chloroform-*d*) δ 161.7, 158.2, 154.0, 135.5, 134.1, 130.5, 130.3, 129.1 (2C), 128.9 (2C), 128.01 (2C), 127.97 (2C), 116.0, 109.1, 100.1, 35.0.

For **3aa** (gram-scale), a mixture of 1,3-diphenylprop-2-yn-1-one **1aa** (12.0 mmol, 2.47 g), 2-cyano-*N*-methylacetamide **2aa** (10 mmol, 0.98 g), and K_2_CO_3_ (12.0 mmol, 1.66 g) in 1,4-dioxane (15 mL) was stirred at 100 °C for 8 h. After completion of the reaction (monitored by TLC), it was cooled down to room temperature, and the resulting solution was poured into water (100 mL), then extracted with EtOAc (3 × 50 mL). The combined EtOAc extracts were dried over Na_2_SO_4_ and concentrated. Then, the solvent was evaporated, and the residue was purified by chromatography (silica gel, PE/EtOAc = 3/1 *v*/*v*) to give **3aa** in 83% yield (2.37 g).

A similar procedure was used for the preparation of products **3ab–3ck**.

1-methyl-2-oxo-4-phenyl-6-(p-tolyl)-1,2-dihydropyridine-3-carbonitrile (**3ab**).

The compound was purified by column chromatography (silica gel, PE/EtOAc = 3/1 *v*/*v*): 130 mg, 87% yield; yellow soild; mp 131–134 °C. ^1^H NMR (400 MHz, CDCl_3_) δ 7.55 (s, 2H), 7.38 (s, 3H), 7.26 (d, *J* = 12.9 Hz, 4H), 6.23 (s, 1H), 3.41 (s, 3H), 2.35 (s, 3H). ^13^C NMR (100 MHz, CDCl_3_) δ 161.9, 158.2, 154.6, 140.8, 135.8, 131.4, 130.6, 129.8 (2C), 129.0 (2C), 128.2 (2C), 128.1 (2C), 116.3, 109.3, 99.7, 35.2, 21.5. HRMS (ESI-TOF) *m/z*: (M+H)^+^ Calcd for C_20_H_17_N_2_O^+^ 301.1335; found, 301.1342.

1-methyl-2-oxo-4-phenyl-6-(m-tolyl)-1,2-dihydropyridine-3-carbonitrile (**3ac**). The compound was purified by column chromatography (silica gel, PE/EtOAc = 3/1 *v*/*v*): 122 mg, 81% yield; yellow soild; mp 157–160 °C. ^1^H NMR (400 MHz, CDCl_3_) δ 7.61 (s, 2H), 7.47 (s, 3H), 7.38 (t, *J* = 7.5 Hz, 1H), 7.31 (d, *J* = 7.9 Hz, 1H), 7.17 (s, 2H), 6.29 (s, 1H), 3.45 (s, 3H), 2.41 (s, 3H). ^13^C NMR (100 MHz, CDCl_3_) δ 161.8, 158.2, 154.3, 139.1, 135.6, 134.1, 131.0, 130.6, 128.9, 128.9 (2C), 128.5, 128.0 (2C), 125.0, 116.0, 109.1, 100.1, 35.1, 21.4. HRMS (ESI-TOF) *m*/*z*: (M+H)^+^ Calcd for C_20_H_17_N_2_O^+^ 301.1335; found, 301.1342.

1-methyl-2-oxo-4-phenyl-6-(o-tolyl)-1,2-dihydropyridine-3-carbonitrile (**3ad**). The compound was purified by column chromatography (silica gel, PE/EtOAc = 3/1 *v*/*v*): 109 mg, 73% yield; yellow soild; mp 170–173 °C. ^1^H NMR (400 MHz, CDCl_3_) δ 7.63 (s, 2H), 7.47 (s, 3H), 7.41 (t, *J* = 7.5 Hz, 1H), 7.32 (d, *J* = 7.5 Hz, 2H), 7.19 (d, *J* = 8.0 Hz, 1H), 6.26 (s, 1H), 3.30 (s, 3H), 2.19 (s, 3H). ^13^C NMR (100 MHz, CDCl_3_) δ 161.6, 158.3, 153.7, 135.5, 135.4, 133.8, 130.8, 130.6, 130.3, 128.9 (2C), 128.1 (2C), 128.0, 126.6, 115.9, 108.6, 100.2, 33.7, 19.3. HRMS (ESI-TOF) *m*/*z*: (M+H)^+^ Calcd for C_20_H_17_N_2_O^+^ 301.1335; found, 301.1345.

6-(4-methoxyphenyl)-1-methyl-2-oxo-4-phenyl-1,2-dihydropyridine-3-carbonitrile (**3ae**). The compound was purified by column chromatography (silica gel, PE/EtOAc = 2/1 *v*/*v*): 136 mg, 86% yield; yellow liquid. ^1^H NMR (400 MHz, CDCl_3_) δ 7.61 (s, 2H), 7.47 (s, 3H), 7.31 (s, 2H), 7.01 (s, 2H), 6.27 (s, 1H), 3.86 (s, 3H), 3.47 (s, 3H). ^13^C NMR (100 MHz, CDCl_3_) δ 161.9, 161.1, 158.1, 154.1, 135.7, 130.5, 129.6 (2C), 128.9 (2C), 128.0 (2C), 126.4, 116.1, 114.4 (2C), 109.2, 99.8, 55.5, 35.1. HRMS (ESI-TOF) *m*/*z*: (M+H)^+^ Calcd for C_20_H_17_N_2_O_2_^+^ 317.1285; found, 317.1297.

6-(2-methoxyphenyl)-1-methyl-2-oxo-4-phenyl-1,2-dihydropyridine-3-carbonitrile (**3af**). The compound was purified by column chromatography (silica gel, PE/EtOAc = 1/1 *v*/*v*): 119 mg, 75% yield; yellow soild; mp 173–176 °C. ^1^H NMR (400 MHz, CDCl_3_) δ 7.64 (s, 2H), 7.46 (s, 4H), 7.25 (s, 1H), 7.05 (d, *J* = 2.7 Hz, 2H), 6.27 (s, 1H), 3.83 (s, 3H), 3.37 (s, 3H). ^13^C NMR (100 MHz, CDCl_3_) δ 161.8, 158.3, 156.4, 152.3, 135.9, 132.3, 130.6, 129.8, 129.0 (2C), 128.3 (2C), 123.3, 121.3, 116.4, 111.2, 109.4, 100.2, 55.7, 34.0. HRMS (ESI-TOF) *m*/*z*: (M+H)^+^ Calcd for C_20_H_17_N_2_O_2_^+^ 317.1285; found, 317.1289.

6-(4-fluorophenyl)-1-methyl-2-oxo-4-phenyl-1,2-dihydropyridine-3-carbonitrile (**3ag**). The compound was purified by column chromatography (silica gel, PE/EtOAc = 3/1 *v*/*v*): 114 mg, 75% yield; brown soild; mp 147–150 °C. ^1^H NMR (400 MHz, CDCl_3_) δ 7.59 (s, 2H), 7.46 (s, 3H), 7.40 (s, 2H), 7.20 (d, *J* = 7.7 Hz, 2H), 6.28 (s, 1H), 3.43 (s, 3H). ^13^C NMR (100 MHz, CDCl_3_) δ 164.9 (d, *J* = 250.9 Hz), 161.6, 158.2, 152.9, 135.4, 130.6, 130.3 (d, *J* = 46.0 Hz, 2C), 128.9 (2C), 128.0 (2C), 116.5 (d, *J* = 22.0 Hz, 2C), 115.9, 109.3, 100.4, 35.0. ^19^F NMR (376 MHz, CDCl_3_) δ −109.13 (s, 1F). HRMS (ESI-TOF) *m*/*z*: (M+Na)^+^ Calcd for C_19_H_13_FN_2_NaO^+^ 327.0904; found, 327.0906.

6-(3-fluorophenyl)-1-methyl-2-oxo-4-phenyl-1,2-dihydropyridine-3-carbonitrile (**3ah**). The compound was purified by column chromatography (silica gel, PE/EtOAc = 3/1 *v*/*v*): 111 mg, 73% yield; brown soild; mp 128–131 °C. ^1^H NMR (400 MHz, CDCl_3_) δ 7.59 (s, 2H), 7.47 (s, 4H), 7.21 (d, *J* = 9.3 Hz, 2H), 7.14 (d, *J* = 8.8 Hz, 1H), 6.30 (s, 1H), 3.45 (s, 3H). ^13^C NMR (100 MHz, CDCl_3_) δ 164.1 (d, *J* = 248.7 Hz), 161.8, 158.5, 152.7, 136.2 (d, *J* = 7.4 Hz), 135.6, 131.4 (d, *J* = 8.2 Hz), 131.0, 129.2 (2C), 128.3 (2C), 124.3, 117.8 (d, *J* = 20.8 Hz), 116.1, 115.8 (d, *J* = 22.9 Hz), 109.4, 101.0, 35.3. ^19^F NMR (376 MHz, CDCl_3_) δ −110.32 (s, 1F). HRMS (ESI-TOF) *m*/*z*: (M+Na)^+^ Calcd for C_19_H_13_FN_2_NaO^+^ 327.0904; found, 327.0912.

6-(2-fluorophenyl)-1-methyl-2-oxo-4-phenyl-1,2-dihydropyridine-3-carbonitrile (**3ai**). The compound was purified by column chromatography (silica gel, PE/EtOAc = 3/1 *v*/*v*): 97 mg, 64% yield; brown soild; mp 161–164 °C. ^1^H NMR (400 MHz, CDCl_3_) δ 7.62 (s, 2H), 7.53 (s, 1H), 7.47 (s, 3H), 7.34 (d, *J* = 5.7 Hz, 2H), 7.24 (s, 1H), 6.33 (s, 1H), 3.42 (s, 3H). ^13^C NMR (100 MHz, CDCl3) δ 161.4, 160.2 (d, *J* = 248.9 Hz), 158.2, 148.6, 135.4, 132.8 (d, *J* = 8.1 Hz), 130.7, 130.1, 128.9 (2C), 128.1 (2C), 125.2, 122.1 (d, *J* = 15.5 Hz), 116.4 (d, *J* = 20.9 Hz), 115.8, 109.7, 101.1, 34.2. ^19^F NMR (376 MHz, CDCl_3_) δ −112.26 (s, 1F). HRMS (ESI-TOF) *m*/*z*: (M+Na)^+^ Calcd for C_19_H_13_FN_2_NaO^+^ 327.0904; found, 327.0907.

6-(4-chlorophenyl)-1-methyl-2-oxo-4-phenyl-1,2-dihydropyridine-3-carbonitrile (**3aj**). The compound was purified by column chromatography (silica gel, PE/EtOAc = 3/1 *v*/*v*): 122 mg, 76% yield; brown soild; mp 171–174 °C. ^1^H NMR (400 MHz, CDCl_3_) δ 7.57 (d, *J* = 7.9 Hz, 2H), 7.49 (s, 1H), 7.47 (s, 1H), 7.44 (s, 2H), 7.42 (d, *J* = 1.7 Hz, 1H), 7.37 (s, 1H), 7.36 (s, 1H), 6.27 (s, 1H), 3.42 (s, 3H). ^13^C NMR (100 MHz, CDCl_3_) δ 161.7, 158.3, 153.0, 136.8, 135.5, 132.6, 130.8, 129.7 (2C), 129.6(2C), 129.1 (2C), 128.2 (2C), 116.1, 109.4, 100.5, 35.1. HRMS (ESI-TOF) *m*/*z*: (M+Na)^+^ Calcd for C_19_H_13_ClN_2_NaO^+^ 343.0609; found, 343.0616.

6-(3-chlorophenyl)-1-methyl-2-oxo-4-phenyl-1,2-dihydropyridine-3-carbonitrile (**3ak**). The compound was purified by column chromatography (silica gel, PE/EtOAc = 3/1 *v*/*v*): 119 mg, 74% yield; brown soild; mp 162–165 °C. ^1^H NMR (400 MHz, CDCl_3_) δ 7.60 (s, 2H), 7.48 (s, 4H), 7.39 (s, 1H), 7.30 (s, 1H), 7.24 (s, 1H), 6.29 (s, 1H), 3.44 (s, 3H). ^13^C NMR (100 MHz, CDCl_3_) δ 161.4, 158.3, 152.2, 135.7, 135.3, 135.2, 130.7, 130.5, 130.5, 129.0 (2C), 128.1, 128.0 (2C), 126.2, 115.8, 109.2, 100.8, 35.0. HRMS (ESI-TOF) *m/z*: (M+Na)^+^ Calcd for C_19_H_13_ClN_2_NaO^+^ 343.0609; found, 327.0610.

6-(2-chlorophenyl)-1-methyl-2-oxo-4-phenyl-1,2-dihydropyridine-3-carbonitrile (**3al**). The compound was purified by column chromatography (silica gel, PE/EtOAc = 3/1 *v*/*v*): 108 mg, 67% yield; brown soild; mp 151–154 °C. ^1^H NMR (400 MHz, CDCl_3_) δ 7.64 (s, 2H), 7.54 (d, *J* = 7.9 Hz, 1H), 7.48 (s, 4H), 7.43 (d, *J* = 7.4 Hz, 1H), 7.34 (d, *J* = 7.8 Hz, 1H), 6.28 (s, 1H), 3.37 (s, 3H). ^13^C NMR (100 MHz, CDCl_3_) δ 161.5, 158.5, 151.1, 135.5, 133.3, 132.9, 131.9, 130.8, 130.30, 130.0, 129.1 (2C), 128.3 (2C), 127.8, 116.0, 109.1, 101.2, 33.8. HRMS (ESI-TOF) *m*/*z*: (M+H)^+^ Calcd for C_19_H_14_ClN_2_O^+^ 321.0789; found, 321.0786.

6-(4-bromophenyl)-1-methyl-2-oxo-4-phenyl-1,2-dihydropyridine-3-carbonitrile (**3am**). The compound was purified by column chromatography (silica gel, PE/EtOAc = 3/1 *v*/*v*): 137 mg, 76% yield; brown soild; mp 176–179 °C. ^1^H NMR (400 MHz, CDCl_3_) δ 7.64 (d, *J* = 8.6 Hz, 2H), 7.57 (d, *J* = 8.0 Hz, 2H), 7.43 (d, *J* = 7.7 Hz, 3H), 7.30 (d, *J* = 8.6 Hz, 2H), 6.27 (s, 1H), 3.42 (s, 3H). ^13^C NMR (100 MHz, CDCl_3_) δ 161.7, 158.3, 153.0, 135.5, 133.1, 132.5 (2C), 130.8, 129.9 (2C), 129.1 (2C), 128.2 (2C), 125.0, 116.1, 109.3, 100.5, 35.1. HRMS (ESI-TOF) *m*/*z*: (M+H)^+^ Calcd for C_19_H_14_BrN_2_O^+^ 365.0284; found, 365.0287.

6-(2-bromophenyl)-1-methyl-2-oxo-4-phenyl-1,2-dihydropyridine-3-carbonitrile (**3an**). The compound was purified by column chromatography (silica gel, PE/EtOAc = 3/1 *v*/*v*): 113 mg, 62% yield; yellow soild; mp 187–190 °C. ^1^H NMR (400 MHz, CDCl_3_) δ 7.69 (d, *J* = 6.5 Hz, 1H), 7.61 (s, 2H), 7.45 (s, 4H), 7.37 (d, *J* = 7.9 Hz, 2H), 6.25 (s, 1H), 3.33 (s, 3H). ^13^C NMR (100 MHz, CDCl_3_) δ 161.4, 158.5, 152.4, 135.5, 135.3, 133.4, 132.0, 130.8, 130.0, 129.0 (2C), 128.4, 128.3 (2C), 122.3, 116.0, 109.1, 101.0, 33.9. HRMS (ESI-TOF) *m*/*z*: (M+H)^+^ Calcd for C_19_H_14_BrN_2_O^+^ 365.0284; found, 365.0267.

1-methyl-2-oxo-4-phenyl-6-(4-(trifluoromethyl)phenyl)-1,2-dihydropyridine-3-carbonitrile (**3ao**). The compound was purified by column chromatography (silica gel, PE/EtOAc = 3/1 *v*/*v*): 115 mg, 65% yield; yellow soild; mp 166–169 °C. ^1^H NMR (400 MHz, CDCl_3_) δ 7.78 (s, 2H), 7.58 (s, 4H), 7.47 (s, 3H), 6.30 (s, 1H), 3.43 (s, 3H). ^13^C NMR (100 MHz, CDCl_3_) δ 161.4, 158.3, 152.2, 137.5, 135.2, 132.6 (d, *J* = 32.7 Hz), 130.8, 129.0 (2C), 128.7 (2C), 128.0 (2C), 126.2, 126.2, 124.8, 115.7, 109.2, 101.0, 35.0. ^19^F NMR (376 MHz, CDCl_3_) δ −62.80 (s, 3F). HRMS (ESI-TOF) *m*/*z*: (M+H)^+^ Calcd for C_20_H_14_F_3_N_2_O^+^ 355.1053; found, 355.1069.

6-(4-fluoro-2-methylphenyl)-1-methyl-2-oxo-4-phenyl-1,2-dihydropyridine-3-carbonitrile (**3ap**). The compound was purified by column chromatography (silica gel, PE/EtOAc = 3/1 *v*/*v*): 112 mg, 70% yield; yellow soild; mp 187–190 °C. ^1^H NMR (400 MHz, CDCl_3_) δ 7.60 (s, 2H), 7.44 (s, 3H), 7.22 (s, 1H), 7.02 (s, 2H), 6.22 (s, 1H), 3.28 (s, 3H), 2.20 (s, 3H). ^13^C NMR (100 MHz, CDCl_3_) δ 164.9 (d, *J* = 249.5 Hz), 161.8, 158.5, 153.0, 138.8 (d, *J* = 8.4 Hz), 135.6, 130.9, 130.4 (d, *J* = 22.7 Hz), 129.1 (2C), 128.3 (2C), 118.0 (d, *J* = 22.0 Hz), 116.1, 114.1 (d, *J* = 22.0 Hz), 109.2, 109.2, 100.7, 34.0, 19.8. ^19^F NMR (376 MHz, CDCl_3_) δ −110.18 (s, 1F). HRMS (ESI-TOF) *m*/*z*: (M+Na)^+^ Calcd for C_20_H_15_FN_2_NaO^+^ 341.1061; found, 341.1061.

6-(2,4-dichlorophenyl)-1-methyl-2-oxo-4-phenyl-1,2-dihydropyridine-3-carbonitrilee (**3aq**). The compound was purified by column chromatography (silica gel, PE/EtOAc = 3/1 *v*/*v*): 98 mg, 55% yield; yellow liquid; ^1^H NMR (400 MHz, CDCl_3_) δ 7.60 (d, *J* = 7.9 Hz, 2H), 7.54 (s, 1H), 7.45 (s, 2H), 7.43 (s, 1H), 7.41 (s, 1H), 7.36 (d, *J* = 8.3 Hz, 1H), 6.25 (s, 1H), 3.34 (s, 3H). ^13^C NMR (100 MHz, CDCl_3_) δ 161.3, 158.4, 150.0, 137.5, 135.3, 133.8, 131.7, 131.1, 130.9, 130.2, 129.1 (2C), 128.3, 128.2 (2C), 115.9, 109.3, 101.4, 33.8. HRMS (ESI-TOF) *m*/*z*: (M+H)^+^ Calcd for C_19_H_13_Cl_2_N_2_O^+^ 355.0399; found, 355.0397.

6-(2,3-dichlorophenyl)-1-methyl-2-oxo-4-phenyl-1,2-dihydropyridine-3-carbonitrile (**3ar**). The compound was purified by column chromatography (silica gel, PE/EtOAc = 3/1 *v*/*v*): 103 mg, 58% yield; brown soild; mp 204–207 °C. ^1^H NMR (400 MHz, CDCl_3_) δ 7.59 (s, 3H), 7.44 (s, 3H), 7.35 (s, 1H), 7.27 (s, 1H), 6.23 (s, 1H), 3.31 (s, 3H). ^13^C NMR (100 MHz, CDCl_3_) δ 161.2, 158.5, 150.3, 135.3, 135.2, 134.4, 132.5, 131.46, 130.9, 129.1 (2C), 128.5, 128.2 (3C), 115.8, 108.9, 101.5, 33.8. HRMS (ESI-TOF) *m*/*z*: (M+Na)^+^ Calcd for C_19_H_12_Cl_2_N_2_NaO^+^ 377.0219; found, 377.0222.

6-(2-chloro-4-fluorophenyl)-1-methyl-2-oxo-4-phenyl-1,2-dihydropyridine-3-carbonitrile (**3as**). The compound was purified by column chromatography (silica gel, PE/EtOAc = 2/1 *v*/*v*): 95 mg, 56% yield; brown soild; mp 151–154 °C. ^1^H NMR (400 MHz, CDCl_3_) δ 7.61 (s, 2H), 7.46 (s, 3H), 7.38 (s, 1H), 7.29 (d, *J* = 5.4 Hz, 1H), 7.15 (s, 1H), 6.26 (s, 1H), 3.34 (s, 3H). ^13^C NMR (100 MHz, CDCl_3_) δ 164.7 (d, *J* = 274.1 Hz), 161.2, 158.3, 149.9, 135.2, 134.2 (d, *J* = 10.2 Hz), 131.4 (d, *J* = 9.1 Hz), 130.8, 129.5, 129.4, 128.9 (2C), 128.1 (2C), 118.0 (d, *J* = 25.0 Hz), 115.7, 115.3 (d, *J* = 21.5 Hz), 109.3, 101.3, 33.7. ^19^F NMR (376 MHz, CDCl_3_) δ −106.66 (s, 1F). HRMS (ESI-TOF) *m*/*z*: (M+H)^+^ Calcd for C_19_H_13_ClFN_2_O^+^ 339.0695; found, 339.0694.

6-(2,4-difluorophenyl)-1-methyl-2-oxo-4-phenyl-1,2-dihydropyridine-3-carbonitrile (**3at**). The compound was purified by column chromatography (silica gel, PE/EtOAc = 2/1 *v*/*v*): 92 mg, 57% yield; yellow soild; mp 136–139 °C. ^1^H NMR (400 MHz, CDCl_3_) δ 7.61 (s, 1H), 7.59 (s, 1H), 7.45 (d, *J* = 7.3 Hz, 3H), 7.41 (s, 1H), 7.08–7.03 (m, 1H), 7.01–6.96 (m, 1H), 6.30 (s, 1H), 3.41 (s, 3H). ^13^C NMR (100 MHz, CDCl_3_) δ 166.0 (d, *J* = 11.5 Hz), 163.4 (d, *J* = 11.6 Hz), 161.5, 161.0 (d, *J* = 12.2 Hz), 158.4, 147.8, 135.5, 131.7 (d, *J* = 9.6 Hz), 131.0, 129.2 (2C), 128.3 (2C), 118.7 (d, *J* = 15.8 Hz), 116.0, 113.1 (d, *J* = 21.6 Hz), 110.2, 105.5 (d, *J* = 50.4 Hz), 105.2, 101.5, 34.4. ^19^F NMR (376 MHz, CDCl_3_) δ −104.13 (s, 1F), −107.54 (s, 1F). HRMS (ESI-TOF) *m*/*z*: (M+H)^+^ Calcd for C_19_H_13_F_2_N_2_O^+^ 323.0990; found, 323.0997.

1-methyl-6-(naphthalen-2-yl)-2-oxo-4-phenyl-1,2-dihydropyridine-3-carbonitrile (**3au**). The compound was purified by column chromatography (silica gel, PE/EtOAc = 2/1 *v*/*v*): 152 mg, 90% yield; yellow soild; mp 191–194 °C. ^1^H NMR (400 MHz, CDCl_3_) δ 7.98 (d, *J* = 8.5 Hz, 1H), 7.90 (s, 3H), 7.66–7.63 (m, 2H), 7.62–7.58 (m, 2H), 7.48 (s, 3H), 7.46–7.43 (m, 1H), 6.41 (s, 1H), 3.50 (s, 3H). ^13^C NMR (100 MHz, CDCl_3_) δ 161.7, 158.2, 154.1, 135.6, 133.5, 132.7, 131.4, 130.6, 128.9, 128.9 (2C), 128.3, 128.0 (3C), 127.9, 127.9, 127.4, 124.6, 116.0, 109.4, 100.2, 35.2. HRMS (ESI-TOF) *m*/*z*: (M+H)^+^ Calcd for C_23_H_17_N_2_O^+^ 337.1335; found, 337.1347.

6-cyclopropyl-1-methyl-2-oxo-4-phenyl-1,2-dihydropyridine-3-carbonitrile (**3av**). The compound was purified by column chromatography (silica gel, PE/EtOAc = 2/1 *v*/*v*): 104 mg, 83% yield; yellow soild; mp 154–157 °C. ^1^H NMR (400 MHz, CDCl_3_) δ 7.52 (s, 2H), 7.45 (s, 3H), 6.09 (s, 1H), 3.74 (s, 3H), 1.89 (s, 1H), 1.16 (s, 2H), 0.87 (s, 2H). ^13^C NMR (100 MHz, CDCl_3_) δ 161.7, 158.4, 155.7, 135.9, 130.3, 128.8 (2C), 127.9 (2C), 116.1, 105.7, 99.0, 31.6, 15.0, 7.6 (2C). HRMS (ESI-TOF) *m*/*z*: (M+Na)^+^ Calcd for C_16_H_14_N_2_NaO^+^ 273.0998; found, 273.1005.

1,6-dimethyl-2-oxo-4-phenyl-1,2-dihydropyridine-3-carbonitrile (**3aw**). The compound was purified by column chromatography (silica gel, PE/EtOAc = 3/1 *v*/*v*): 170 mg, 76% yield; yellow soild; mp 164–167 °C. ^1^H NMR (400 MHz, CDCl_3_) δ 7.56 (s, 2H), 7.47 (s, 3H), 6.23 (s, 1H), 3.58 (s, 3H), 2.46 (s, 3H). ^13^C NMR (100 MHz, CDCl_3_) δ 161.7, 158.1, 151.6, 135.6, 130.4, 128.8 (2C), 127.9 (2C), 116.1, 108.1, 98.7, 31.8, 21.6.

1-methyl-2-oxo-4-phenyl-6-(thiophen-2-yl)-1,2-dihydropyridine-3-carbonitrile (**3ax**). The compound was purified by column chromatography (silica gel, PE/EtOAc = 3/1 *v*/*v*): 123 mg, 84% yield; yellow soild; mp 144–147 °C. ^1^H NMR (400 MHz, CDCl_3_) δ 7.61 (s, 2H), 7.55 (d, *J* = 3.9 Hz, 1H), 7.48 (d, *J* = 2.7 Hz, 3H), 7.29 (d, *J* = 3.7 Hz, 1H), 7.16 (d, *J* = 1.4 Hz, 1H), 6.46 (s, 1H), 3.62 (s, 3H). ^13^C NMR (100 MHz, CDCl_3_) δ 161.6, 157.8, 147.0, 135.4, 134.1, 130.6, 130.0, 129.2, 128.9, 128.0 (2C), 127.9 (2C), 115.9, 110.4, 100.6, 35.0. HRMS (ESI-TOF) *m*/*z*: (M+Na)^+^ Calcd for C_17_H_12_N_2_NaOS^+^ 315.0563; found, 315.0567.

1-methyl-2-oxo-6-phenyl-4-(p-tolyl)-1,2-dihydropyridine-3-carbonitrile (**3ba**). The compound was purified by column chromatography (silica gel, PE/EtOAc = 3/1 *v*/*v*): 132 mg, 88% yield; white soild; mp 129–132 °C. ^1^H NMR (400 MHz, CDCl_3_) δ 7.50 (s, 5H), 7.37 (s, 2H), 7.26 (s, 2H), 6.28 (s, 1H), 3.42 (s, 3H), 2.37 (s, 3H). ^13^C NMR (100 MHz, CDCl_3_) δ 161.8, 158.1, 153.8, 141.0, 134.2, 132.6, 130.3, 129.6 (2C), 129.1 (2C), 128.0 (4C), 116.2, 109.1, 99.8, 35.9, 21.4.

1-methyl-2-oxo-6-phenyl-4-(m-tolyl)-1,2-dihydropyridine-3-carbonitrile (**3bb**). The compound was purified by column chromatography (silica gel, PE/EtOAc = 3/1 *v*/*v*): 126 mg, 84% yield; yellow soild; mp 159–162 °C. ^1^H NMR (400 MHz, CDCl_3_) δ 7.50 (s, 3H), 7.39 (s, 4H), 7.32 (s, 1H), 7.27 (s, 1H), 6.28 (s, 1H), 3.44 (s, 3H), 2.36 (s, 3H). ^13^C NMR (100 MHz, CDCl_3_) δ 161.9, 158.5, 154.1, 138.8, 135.7, 134.3, 131.4, 130.4, 129.2 (2C), 128.9, 128.7, 128.2 (2C), 125.3, 116.2, 109.3, 100.2, 35.1, 21.5. HRMS (ESI-TOF) *m*/*z*: (M+H)^+^ Calcd for C_20_H_17_N_2_O^+^ 301.1335; found, 301.134.

4-(4-butylphenyl)-1-methyl-2-oxo-6-phenyl-1,2-dihydropyridine-3-carbonitrile (**3bc**). The compound was purified by column chromatography (silica gel, PE/EtOAc = 3/1 *v*/*v*): 146 mg, 85% yield; white liquid. ^1^H NMR (400 MHz, CDCl_3_) δ 7.55 (d, *J* = 8.2 Hz, 2H), 7.51 (s, 3H), 7.38 (s, 2H), 7.27 (d, *J* = 8.3 Hz, 2H), 6.29 (s, 1H), 3.44 (s, 3H), 2.63 (s, 2H), 1.59 (s, 2H), 1.35 (s, 2H), 0.90 (s, 3H). ^13^C NMR (100 MHz, CDCl_3_) δ 161.8, 158.2, 153.8, 146.0, 134.3, 132.8, 130.3, 129.1 (2C), 129.0 (2C), 128.0 (2C), 128.0 (2C), 116.2, 109.1, 99.8, 35.5, 35.0, 33.3, 22.3, 13.9. HRMS (ESI-TOF) *m*/*z*: (M+H)^+^ Calcd for C_23_H_23_N_2_O^+^ 343.1805; found, 343.1818.

4-(4-methoxyphenyl)-1-methyl-2-oxo-6-phenyl-1,2-dihydropyridine-3-carbonitrile (**3bd**). The compound was purified by column chromatography (silica gel, PE/EtOAc = 1/1 *v*/*v*): 151 mg, 95% yield; yellow soild; mp 139–142 °C. ^1^H NMR (400 MHz, CDCl_3_) δ 7.56 (d, *J* = 8.9 Hz, 2H), 7.47 (s, 3H), 7.38 (s, 2H), 6.90 (d, *J* = 8.9 Hz, 2H), 6.26 (s, 1H), 3.77 (s, 3H), 3.38 (s, 3H). ^13^C NMR (100 MHz, CDCl_3_) δ 162.0, 161.6, 157.6, 154.0, 134.4, 130.4, 130.0 (2C), 129.2 (2C), 128.2 (2C), 127.7, 116.7, 114.4 (2C), 109.2, 99.1, 55.5, 35.0. HRMS (ESI-TOF) *m/z*: (M+H)^+^ Calcd for C_20_H_17_N_2_O_2_^+^ 317.1285; found, 317.1293.

4-(3-methoxyphenyl)-1-methyl-2-oxo-6-phenyl-1,2-dihydropyridine-3-carbonitrile (**3be**). The compound was purified by column chromatography (silica gel, PE/EtOAc = 1/1 *v*/*v*): 146 mg, 92% yield; yellow soild; mp 149–152 °C. ^1^H NMR (400 MHz, CDCl_3_) δ 7.50 (s, 3H), 7.39 (s, 2H), 7.33 (d, *J* = 5.8 Hz, 1H), 7.16 (d, *J* = 7.8 Hz, 1H), 7.11 (s, 1H), 6.99 (s, 1H), 6.30 (s, 1H), 3.79 (s, 3H), 3.44 (s, 3H). ^13^C NMR (100 MHz, CDCl_3_) δ 161.8, 159.8, 158.2, 154.3, 136.9, 135.0, 130.5, 130.1, 129.2 (2C), 128.2 (2C), 120.5, 116.6, 116.1, 113.4, 109.3, 100.2, 55.6, 35.2. HRMS (ESI-TOF) *m*/*z*: (M+H)^+^ Calcd for C_20_H_17_N_2_O_2_^+^ 317.1285; found, 317.1292.

4-(4-fluorophenyl)-1-methyl-2-oxo-6-phenyl-1,2-dihydropyridine-3-carbonitrile (**3bf**). The compound was purified by column chromatography (silica gel, PE/EtOAc = 3/1 *v*/*v*): 113 mg, 74% yield; brown soild; mp 172–175 °C. ^1^H NMR (400 MHz, CDCl_3_) δ 7.62 (s, 2H), 7.52 (s, 3H), 7.38 (s, 2H), 7.14 (d, *J* = 7.6 Hz, 2H), 6.26 (s, 1H), 3.44 (s, 3H). ^13^C NMR (100 MHz, CDCl_3_) δ 165.3, 162.8, 161.6, 157.1, 154.3, 134.1, 131.6, 130.4 (2C), 130.3 (d, *J* = 8.7 Hz, 2C), 129.1 (2C), 128.0 (2C), 116.2 (d, *J* = 21.8 Hz), 108.9, 100.1, 35.1. ^19^F NMR (376 MHz, CDCl_3_) δ −109.15 (s, 1F).

4-(4-chlorophenyl)-1-methyl-2-oxo-6-phenyl-1,2-dihydropyridine-3-carbonitrile (**3bg**). The compound was purified by column chromatography (silica gel, PE/EtOAc = 3/1 *v*/*v*): 121 mg, 75% yield; brown soild; mp 165–168 °C. ^1^H NMR (400 MHz, CDCl_3_) δ 7.56 (s, 1H), 7.53 (s, 1H), 7.51 (d, *J* = 2.3 Hz, 3H), 7.42 (s, 1H), 7.40 (s, 3H), 6.26 (s, 1H), 3.44 (s, 3H). ^13^C NMR (100 MHz, CDCl_3_) δ 161.6, 157.0, 154.6, 137.0, 134.2, 134.1, 130.6, 129.6 (2C), 129.3 (2C), 129.3 (2C), 128.1 (2C), 116.0, 108.9, 100.1, 35.2. HRMS (ESI-TOF) *m*/*z*: (M+Na)^+^ Calcd for C_19_H_13_ClN_2_NaO^+^ 343.0609; found, 343.0615.

4-(2-chlorophenyl)-1-methyl-2-oxo-6-phenyl-1,2-dihydropyridine-3-carbonitrile (**3bh**). The compound was purified by column chromatography (silica gel, PE/EtOAc = 3/1 *v*/*v*): 97 mg, 60% yield; brown soild; mp 172–175 °C. ^1^H NMR (400 MHz, CDCl_3_) δ 7.49 (d, *J* = 2.4 Hz, 2H), 7.48 (s, 3H), 7.38 (s, 2H), 7.37 (s, 2H), 6.22 (s, 1H), 3.47 (s, 3H). ^13^C NMR (100 MHz, CDCl_3_) δ 161.4, 156.7, 154.1, 134.9, 134.1, 131.8, 131.2, 130.6, 130.4, 130.0, 129.2 (2C), 128.2 (2C), 127.3, 115.2, 110.1, 102.8, 35.3.

4-(4-bromophenyl)-1-methyl-2-oxo-6-phenyl-1,2-dihydropyridine-3-carbonitrile (**3bi**). The compound was purified by column chromatography (silica gel, PE/EtOAc = 3/1 *v*/*v*): 143 mg, 78% yield; white liquid. ^1^H NMR (400 MHz, CDCl_3_) δ 7.58 (s, 2H), 7.51 (s, 5H), 7.38 (s, 2H), 6.25 (s, 1H), 3.44 (s, 3H). ^13^C NMR (100 MHz, CDCl_3_) δ 161.5, 156.9, 154.5, 134.4, 134.0, 132.2 (2C), 130.4, 129.6 (2C), 129.1 (2C), 127.9 (2C), 125.2, 115.8, 108.7, 100.0, 35.1. HRMS (ESI-TOF) *m*/*z*: (M+H)^+^ Calcd for C_19_H_14_BrN_2_O^+^ 365.0284; found, 365.0292.

Methyl 4-(3-cyano-1-methyl-2-oxo-6-phenyl-1,2-dihydropyridin-4-yl)benzoate (**3bj**). The compound was purified by column chromatography (silica gel, PE/EtOAc = 2/1 *v*/*v*): 121 mg, 70% yield; orange soild; mp 121–124 °C. ^1^H NMR (400 MHz, CDCl_3_) δ 8.11 (d, *J* = 8.6 Hz, 2H), 7.67 (d, *J* = 8.5 Hz, 2H), 7.52 (s, 3H), 7.39 (s, 2H), 6.29 (s, 1H), 3.92 (s, 3H), 3.46 (s, 3H). ^13^C NMR (100 MHz, CDCl_3_) δ 166.2, 161.4, 157.1, 154.5, 139.8, 134.0, 131.8, 130.5, 130.1 (2C), 129.2 (2C), 128.2 (2C), 128.0 (2C), 115.6, 108.7, 100.5, 52.4, 35.2. HRMS (ESI-TOF) *m*/*z*: (M+H)^+^ Calcd for C_21_H_17_N_2_O_3_^+^ 345.1234; found, 345.1240.

4-cyclopropyl-1-methyl-2-oxo-6-phenyl-1,2-dihydropyridine-3-carbonitrile (**3bk**). The compound was purified by column chromatography (silica gel, PE/EtOAc = 2/1 *v*/*v*): 109 mg, 87% yield; white soild; mp 167–170 °C. ^1^H NMR (400 MHz, CDCl_3_) δ 7.45 (s, 3H), 7.28 (s, 2H), 5.49 (s, 1H), 3.28 (s, 3H), 2.21 (s, 1H), 1.20 (s, 2H), 0.88 (s, 2H). ^13^C NMR (100 MHz, CDCl_3_) δ 164.2, 161.0, 154.2, 134.5, 130.3, 129.1 (2C), 128.1 (2C), 115.9, 102.8, 101.4, 34.7, 15.0, 11.4 (2C). HRMS (ESI-TOF) *m*/*z*: (M+Na)^+^ Calcd for C_16_H_14_N_2_NaO^+^ 273.0998; found, 273.1001.

4-hexyl-1-methyl-2-oxo-6-phenyl-1,2-dihydropyridine-3-carbonitrile (**3bl**). The compound was purified by column chromatography (silica gel, PE/EtOAc = 3/1 *v*/*v*): 121 mg, 82% yield; yellow soild; mp 115–118 °C. ^1^H NMR (400 MHz, CDCl_3_) δ 7.49 (d, *J* = 2.3 Hz, 3H), 7.32 (d, *J* = 3.1 Hz, 2H), 6.08 (s, 1H), 3.35 (s, 3H), 2.70–2.65 (m, 2H), 1.65–1.57 (m, 2H), 1.36 (s, 2H), 1.27 (d, *J* = 3.3 Hz, 2H), 1.26 (s, 2H), 0.86–0.81 (m, 3H). ^13^C NMR (100 MHz, CDCl_3_) δ 162.5, 161.5, 153.9, 134.3, 130.3, 129.2 (2C), 128.1 (2C), 115.4, 109.2, 102.1, 35.0, 34.9, 31.5, 29.4, 29.0, 22.5, 14.1. HRMS (ESI-TOF) *m*/*z*: (M+Na)^+^ Calcd for C_19_H_22_N_2_NaO^+^ 317.1624; found, 317.1627.

1-methyl-2-oxo-6-phenyl-4-(thiophen-2-yl)-1,2-dihydropyridine-3-carbonitrile (**3bm**). The compound was purified by column chromatography (silica gel, PE/EtOAc = 3/1 *v*/*v*): 117 mg, 80% yield; orange soild; mp 151–154 °C. ^1^H NMR (400 MHz, CDCl_3_) δ 8.00 (d, *J* = 3.0 Hz, 1H), 7.52 (d, *J* = 2.1 Hz, 2H), 7.51 (d, *J* = 1.3 Hz, 1H), 7.49 (s, 1H), 7.42 (d, *J* = 5.1 Hz, 1H), 7.39 (d, *J* = 2.2 Hz, 1H), 7.38 (s, 1H), 6.36 (s, 1H), 3.42 (s, 3H). ^13^C NMR (100 MHz, CDCl_3_) δ 161.9, 154.0, 151.3, 135.9, 134.2, 130.3, 129.1 (2C), 128.24, 128.0 (2C), 127.0, 126.9, 116.6, 108.3, 98.6, 38.3. HRMS (ESI-TOF) *m*/*z*: (M+Na)^+^ Calcd for C_17_H_12_N_2_NaOS^+^ 315.0563; found, 315.0571.

4-(4-methoxyphenyl)-1-methyl-2-oxo-6-(p-tolyl)-1,2-dihydropyridine-3-carbonitrile (**3ca**). The compound was purified by column chromatography (silica gel, PE/EtOAc = 1/1 *v*/*v*): 159 mg, 96% yield; orange soild; mp 155–158 °C. ^1^H NMR (400 MHz, CDCl_3_) δ 7.58 (s, 2H), 7.28 (s, 4H), 6.93 (s, 2H), 6.26 (s, 1H), 3.80 (s, 3H), 3.41 (s, 3H), 2.40 (s, 3H). ^13^C NMR (100 MHz, CDCl_3_) δ 162.1, 161.6, 157.7, 154.1, 140.7, 131.6, 129.9 (2C), 129.8 (2C), 128.1 (2C), 127.9, 116.7, 114.4 (2C), 109.1, 99.1, 55.5, 35.1, 21.5. HRMS (ESI-TOF) *m*/*z*: (M+H)^+^ Calcd for C_21_H_19_N_2_O_2_^+^ 331.1441; found, 331.1447.

4-(4-butylphenyl)-1-methyl-2-oxo-6-(p-tolyl)-1,2-dihydropyridine-3-carbonitrile (**3cb**). The compound was purified by column chromatography (silica gel, PE/EtOAc = 3/1 *v*/*v*): 161 mg, 90% yield; yellow liquid. ^1^H NMR (400 MHz, CDCl_3_) δ 7.55 (s, 1H), 7.53 (s, 1H), 7.29 (s, 2H), 7.27 (s, 2H), 7.25 (s, 2H), 6.28 (s, 1H), 3.45 (s, 3H), 2.63 (t, *J* = 7.8 Hz, 2H), 2.42 (s, 3H), 1.64–1.55 (m, 2H), 1.38–1.31 (m, 2H), 0.91 (d, *J* = 14.6 Hz, 3H). ^13^C NMR (100 MHz, CDCl_3_) δ 162.1, 158.2, 154.2, 146.1, 140.8, 133.0, 131.6, 129.8 (2C), 129.1 (2C), 128.2 (2C), 128.1 (2C), 116.4, 109.3, 99.7, 35.6, 35.1, 33.4, 22.4, 21.5, 14.0. HRMS (ESI-TOF) *m*/*z*: (M+H)^+^ Calcd for C_24_H_25_N_2_O^+^ 357.1961; found, 357.1973.

4,6-bis(4-methoxyphenyl)-1-methyl-2-oxo-1,2-dihydropyridine-3-carbonitrile (**3cc**). The compound was purified by column chromatography (silica gel, PE/EtOAc = 1/1 *v*/*v*): 170 mg, 98% yield; white soild; mp 162–165 °C. ^1^H NMR (400 MHz, CDCl_3_) δ 7.50 (s, 2H), 7.24 (s, 2H), 6.91 (s, 2H), 6.85 (s, 2H), 6.19 (s, 1H), 3.77 (s, 3H), 3.74 (s, 3H), 3.36 (s, 3H). ^13^C NMR (100 MHz, CDCl_3_) δ 162.2, 161.5, 161.1, 157.5, 153.9, 129.8 (2C), 129.7 (2C), 127.8, 126.6, 116.7, 114.5 (2C), 114.3 (2C), 109.2, 98.7, 55.6, 55.5, 35.1.

4-(2-chlorophenyl)-6-(2-fluorophenyl)-1-methyl-2-oxo-1,2-dihydropyridine-3-carbonitrile (**3cd**). The compound was purified by column chromatography (silica gel, PE/EtOAc = 3/1 *v*/*v*): 107 mg, 64% yield; yellow liquid. ^1^H NMR (400 MHz, CDCl_3_) δ 7.54 (s, 1H), 7.47 (d, *J* = 7.4 Hz, 1H), 7.37 (d, *J* = 5.8 Hz, 4H), 7.30 (d, *J* = 7.4 Hz, 1H), 7.22 (d, *J* = 8.5 Hz, 1H), 6.25 (s, 1H), 3.44 (s, 3H). ^13^C NMR (100 MHz, CDCl_3_) δ 161.1, 160.4 (d, *J* = 249.1 Hz), 156.8, 148.8, 134.8, 133.2 (d, *J* = 8.2 Hz), 131.9, 131.4, 130.5, 130.4, 130.2, 127.5, 125.4, 122.1 (d, *J* = 15.4 Hz), 116.7 (d, *J* = 20.6 Hz), 115.1, 110.8, 103.9, 34.6. ^19^F NMR (376 MHz, CDCl_3_) δ −112.17 (s, 1F). HRMS (ESI-TOF) *m*/*z*: (M+H)^+^ Calcd for C_19_H_13_ClFN_2_O^+^ 339.0695; found, 339.0708.

6-(2-bromophenyl)-4-(4-methoxyphenyl)-1-methyl-2-oxo-1,2-dihydropyridine-3-carbonitrile (**3ce**). The compound was purified by column chromatography (silica gel, PE/EtOAc = 2/1 *v*/*v*): 135 mg, 68% yield; yellow liquid. ^1^H NMR (400 MHz, CDCl_3_) δ 7.70 (d, *J* = 8.0 Hz, 1H), 7.64 (d, *J* = 6.6 Hz, 2H), 7.47 (s, 1H), 7.38 (s, 1H), 7.33 (s, 1H), 6.97 (d, *J* = 8.6 Hz, 2H), 6.25 (s, 1H), 3.83 (s, 3H), 3.33 (s, 3H). ^13^C NMR (100 MHz, CDCl_3_) δ 161.7, 161.5, 157.8, 151.8, 135.4, 133.3, 131.7, 129.9 (2C), 129.8, 128.1, 127.5, 122.4, 116.3, 114.3 (2C), 108.7, 100.1, 55.4, 33.62. HRMS (ESI-TOF) *m*/*z*: (M+Na)^+^ Calcd for C_20_H_15_BrN_2_NaO_2_^+^ 417.0209; found, 417.0212.

6-(4-cyanophenyl)-4-(4-methoxyphenyl)-1-methyl-2-oxo-1,2-dihydropyridine-3-carbonitrile (**3cf**). The compound was purified by column chromatography (silica gel, PE/EtOAc = 1/1 *v*/*v*): 92 mg, 70% yield; brown soild; mp 226–229 °C. ^1^H NMR (400 MHz, DMSO-*d*_6_) δ 8.01 (s, 1H), 7.99 (s, 1H), 7.80 (s, 1H), 7.78 (s, 1H), 7.68 (s, 1H), 7.66 (s, 1H), 7.07 (s, 1H), 7.05 (s, 1H), 6.46 (s, 1H), 3.79 (s, 3H), 3.25 (s, 3H). ^13^C NMR (100 MHz, DMSO-*d*_6_) δ 161.8, 161.5, 157.7, 152.9, 138.9, 133.2 (2C), 130.6 (2C), 130.2 (2C), 127.7, 118.8, 117.3, 114.9 (2C), 113.3, 109.2, 98.7, 56.0, 35.2.

4-(2-chlorophenyl)-6-(2,4-difluorophenyl)-1-methyl-2-oxo-1,2-dihydropyridine-3-carbonitrile (**3cg**). The compound was purified by column chromatography (silica gel, PE/EtOAc = 3/1 *v*/*v*): 95 mg, 53% yield; yellow liquid. ^1^H NMR (400 MHz, CDCl_3_) δ 7.46 (s, 1H), 7.37 (s, 4H), 7.01 (s, 1H), 6.98 (s, 1H), 6.23 (s, 1H), 3.44 (s, 3H). ^13^C NMR (100 MHz, CDCl_3_) δ 165.6 (t, *J* = 253.0 Hz, *J* = 488.0 Hz), 160.7, 156.5, 147.3, 134.4, 131.6, 131.2 (2C), 130.3, 129.9, 127.2, 118.2 (d, *J* = 15.5 Hz), 114.7, 112.8 (d, *J* = 21.5 Hz), 110.7, 105.2 (d, *J* = 49.9 Hz), 105.0, 104.0, 34.2. ^19^F NMR (376 MHz, CDCl_3_) δ −104.04 (s, 1F), −107.46 (s, 1F). HRMS (ESI-TOF) *m*/*z*: (M+H)^+^ Calcd for C_19_H_12_ClF_2_N_2_O^+^ 357.0601; found, 357.0614.

4-(2-chlorophenyl)-6-(2,3-dichlorophenyl)-1-methyl-2-oxo-1,2-dihydropyridine-3-carbonitrile (**3ch**). The compound was purified by column chromatography (silica gel, PE/EtOAc = 3/1 *v*/*v*): 115 mg, 59% yield; yellow soild; mp 178–181 °C. ^1^H NMR (400 MHz, CDCl_3_) δ 7.62 (s, 1H), 7.44 (s, 1H), 7.37 (s, 2H), 7.35 (s, 2H), 7.29 (s, 1H), 6.19 (s, 1H), 3.34 (s, 3H). ^13^C NMR (100 MHz, CDCl_3_) δ 160.8, 157.0, 150.2, 134.9, 134.5, 134.4, 132.6, 131.8, 131.4 (2C), 130.4, 130.0, 128.6, 128.2, 127.4, 114.9, 110.0, 104.1, 33.9. HRMS (ESI-TOF) *m*/*z*: (M+H)^+^ Calcd for C_19_H_12_Cl_3_N_2_O^+^ 389.0010; found, 389.0018.

4-(4-butylphenyl)-1-methyl-6-(naphthalen-2-yl)-2-oxo-1,2-dihydropyridine-3-carbonitrile (**3ci**). The compound was purified by column chromatography (silica gel, PE/EtOAc = 3/1 *v*/*v*): 145 mg, 88% yield; yellow soild; mp 144–147 °C. ^1^H NMR (400 MHz, CDCl_3_) δ 7.97 (d, *J* = 8.6 Hz, 1H), 7.91 (s, 3H), 7.59 (d, *J* = 1.5 Hz, 1H), 7.59 (s, 2H), 7.57–7.56 (m, 1H), 7.45 (d, *J* = 10.1 Hz, 1H), 7.26 (d, *J* = 8.3 Hz, 2H), 6.41 (s, 1H), 3.48 (s, 3H), 2.63 (t, *J* = 7.7 Hz, 2H), 1.59 (t, *J* = 8.4 Hz, 2H), 1.38–1.31 (m, 2H), 0.90 (t, *J* = 7.3 Hz, 3H). ^13^C NMR (100 MHz, CDCl_3_) δ 162.0, 158.3, 154.1, 146.1, 133.7, 133.0, 132.9, 131.7, 129.1, 129.1 (2C), 128.5, 128.2 (3C), 128.1, 128.0, 127.5, 124.8, 116.5, 109.6, 99.9, 35.6, 35.2, 33.4, 22.4, 14.0. HRMS (ESI-TOF) *m*/*z*: (M+H)^+^ Calcd for C_27_H_25_N_2_O^+^ 393.1961; found, 393.1964.

4-(2-chlorophenyl)-1-methyl-6-(naphthalen-2-yl)-2-oxo-1,2-dihydropyridine-3-carbonitrile (**3cj**). The compound was purified by column chromatography (silica gel, PE/EtOAc = 3/1 *v*/*v*): 149 mg, 80% yield; orange liquid. ^1^H NMR (400 MHz, CDCl_3_) δ 7.97 (d, *J* = 8.6 Hz, 1H), 7.90 (s, 3H), 7.59 (d, *J* = 3.1 Hz, 2H), 7.48 (s, 1H), 7.45 (s, 1H), 7.41 (s, 1H), 7.38 (d, *J* = 3.0 Hz, 2H), 6.35 (s, 1H), 3.53 (s, 3H). ^13^C NMR (100 MHz, CDCl_3_) δ 161.4, 156.8, 154.2, 134.9, 133.7, 132.9, 131.8, 131.4, 131.3, 130.4, 130.1, 129.1, 128.5, 128.3, 128.1 (2C), 127.6, 127.4, 124.8, 115.2, 110.4, 103.0, 35.5. HRMS (ESI-TOF) *m*/*z*: (M+H)^+^ Calcd for C_23_H_16_ClN_2_O^+^ 371.0946; found, 371.0950.

6-(2-bromophenyl)-4-cyclopropyl-1-methyl-2-oxo-1,2-dihydropyridine-3-carbonitrile (**3ck**). The compound was purified by column chromatography (silica gel, PE/EtOAc = 2/1 *v*/*v*): 99 mg, 60% yield; yellow liquid. ^1^H NMR (400 MHz, CDCl_3_) δ 7.65 (s, 1H), 7.44 (s, 1H), 7.36 (s, 1H), 7.24 (s, 1H), 5.46 (s, 1H), 3.23 (s, 3H), 2.28 (s, 1H), 1.26 (s, 2H), 0.89 (s, 2H). ^13^C NMR (100 MHz, CDCl_3_) δ 164.4, 160.5, 152.1, 135.4, 133.3, 131.6, 129.7, 128.1, 122.3, 115.6, 102.4, 33.3, 15.0, 11.4, 11.3. HRMS (ESI-TOF) *m*/*z*: (M+H)^+^ Calcd for C_16_H_14_BrN_2_O^+^ 329.0284; found, 329.0283.

2-oxo-1,4-diphenyl-6-(m-tolyl)-1,2-dihydropyridine-3-carbonitrile (**3da**). The compound was purified by column chromatography (silica gel, PE/EtOAc = 6/1 *v*/*v*): 100 mg, 53% yield; white soild; mp204–207 °C. ^1^H NMR (400 MHz, CDCl_3_) δ 7.70 (s, 2H), 7.50 (s, 3H), 7.26 (s, 3H), 7.10 (s, 2H), 7.03 (s, 2H), 6.94 (s, 1H), 6.88 (s, 1H), 6.43 (s, 1H), 2.19 (s, 3H). ^13^C NMR (100 MHz, CDCl_3_) δ 161.3, 159.2, 153.7, 138.1, 137.3, 135.7, 134.1, 130.7, 130.2, 129.3, 129.0(2C), 128.9(2C), 128.6(3C), 128.1(2C), 128.0, 125.8, 115.7, 109.3, 101.1, 21.1. HRMS (ESI-TOF) *m*/*z*: (M+Na)^+^ Calcd for C_25_H_18_N_2_NaO^+^ 385.1311; found, 385.1320.

1-benzyl-2-oxo-4-phenyl-6-(m-tolyl)-1,2-dihydropyridine-3-carbonitrile (**3db**). The compound was purified by column chromatography (silica gel, PE/EtOAc = 10/1 *v*/*v*): 125 mg, 69% yield; white liquid. ^1^H NMR (400 MHz, CDCl_3_) δ 7.65 (s, 2H), 7.46 (s, 3H), 7.26 (s, 2H), 7.21 (s, 3H), 6.98 (s, 1H), 6.93 (s, 2H), 6.89 (s, 1H), 6.27 (s, 1H), 5.20 (s, 2H), 2.27 (s, 3H). ^13^C NMR (100 MHz, CDCl_3_) δ 161.8, 158.6, 154.6, 138.7, 136.3, 135.6, 134.0, 131.0, 130.8, 129.1, 129.1 (2C), 128.7, 128.6 (2C), 128.2 (2C), 127.7, 127.5 (2C), 125.3, 116.2, 109.6, 100.9, 49.7, 21.4.

2-oxo-4-phenyl-6-(m-tolyl)-1,2-dihydropyridine-3-carbonitrile (**3dc**). The compound was purified by column chromatography (silica gel, PE/EtOAc = 3/1 *v*/*v*): 129mg, 90% yield; white solid; mp 169–172 °C. ^1^H NMR (400 MHz, CDCl_3_) δ 7.86 (s, 1H), 7.70 (s, 3H), 7.54 (s, 3H), 7.45 (s, 1H), 7.39 (s, 1H), 6.76 (s, 1H), 2.54 (s, 3H), 1.26 (s, 1H). ^13^C NMR (100 MHz, CDCl_3_) δ 164.1, 161.0, 151.0, 139.9, 136.1, 132.7, 131.5, 130.6, 129.3, 129.0 (2C), 128.1 (2C), 127.9, 124.3, 115.7, 106.5, 99.7, 21.4.

### 3.2. Insecticidal Properties Study Methods

The experimental details are presented in the Supplementary Materials.

## 4. Conclusions

In summary, we have developed an efficient and straightforward methodology to construct functional 3-cyano-2-pyridones. The C–C and C–N bond formation were the key procedures of this transformation. Our study presents the utilization of these pyridones for insecticidal properties, and compound **3ci** was selected as the best insecticide. With advantages such as a metal-free process, wide functional group tolerance, simple operation, and mild conditions, further chemical modification and biological exploration of these types of compounds are underway in our laboratory.

## Figures and Tables

**Table 1:** Optimization of the reaction conditions *^a^*.

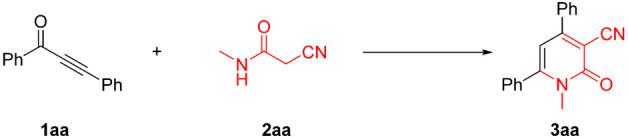			
**Entry**	**Base**	**Solvent**	**Yield(%) *^b^***
1	K_2_CO_3_	1,4-dioxane	81
2	Na_2_CO_3_	1,4-dioxane	70
3	Cs_2_CO_3_	1,4-dioxane	74
4	NaOH	1,4-dioxane	Trace
5	NaH	1,4-dioxane	Trace
6	EtONa	1,4-dioxane	Trace
7	LiO*^t^*Bu	1,4-dioxane	NR
8	NaO*^t^*Bu	1,4-dioxane	Trace
9	KO*^t^*Bu	1,4-dioxane	Trace
10	Et_3_N	1,4-dioxane	NR
11	DBU	1,4-dioxane	NR
12	DABCO	1,4-dioxane	NR
13	K_2_CO_3_	DMSO	54
14	K_2_CO_3_	DMF	66
15	K_2_CO_3_	NMP	50
16	K_2_CO_3_	CH_3_CN	Trace
17	K_2_CO_3_	Toluene	Trace
18	K_2_CO_3_	THF	Trace
19	K_2_CO_3_	CH_3_OH	Trace
20 *^c^*	K_2_CO_3_	1,4-dioxane	45
21 *^d^*	K_2_CO_3_	1,4-dioxane	83
22 *^e^*	K_2_CO_3_	1,4-dioxane	85

*^a^* Reaction conditions: **1aa** (0.6 mmol), **2aa** (0.5 mmol), and base (1.2 mmol) in 2.0 mL of solvent at 100 °C for 8 h under N_2_. *^b^* Isolated yield based on **2aa**. *^c^* 1.0 equiv. K_2_CO_3_. *^d^* 1.5 equiv. K_2_CO_3_. *^e^* 2.5 equiv. K_2_CO_3_. NR = no reaction. DMSO = dimethyl-sulfoxide, DMF = *N,N*-dimethylformamide, THF = tetrahydrofuran, DBU = 1,8-diazabicyclo[5.4.0]undec-7-ene, DABCO = triethylenediamine.

**Table 2:** Chemical structures and synthesis yields of 3-cyano-2-pyridones *^a^*.

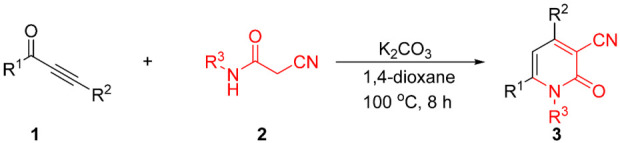					
**Entry**	**R^1^**	**R^2^**	**R^3^**	**3**	**Yield(%) *^b^***
1	Ph	Ph	Me	**3aa**	81
2	4-MeC_6_H_4_	Ph	Me	**3ab**	87
3	3-MeC_6_H_4_	Ph	Me	**3ac**	83
4	2-MeC_6_H_4_	Ph	Me	**3ad**	73
5	4-OMeC_6_H_4_	Ph	Me	**3ae**	86
6	2-OMeC_6_H_4_	Ph	Me	**3af**	75
7	4-FC_6_H_4_	Ph	Me	**3ag**	75
8	3-FC_6_H_4_	Ph	Me	**3ah**	73
9	2-FC_6_H_4_	Ph	Me	**3ai**	64
10	4-ClC_6_H_4_	Ph	Me	**3aj**	76
11	3-ClC_6_H_4_	Ph	Me	**3ak**	74
12	2-ClC_6_H_4_	Ph	Me	**3al**	67
13	4-BrC_6_H_4_	Ph	Me	**3am**	75
14	2-BrC_6_H_4_	Ph	Me	**3an**	62
15	4-CF_3_C_6_H_4_	Ph	Me	**3ao**	65
16	2-Me-4-FC_6_H_3_	Ph	Me	**3ap**	70
17	2,4-ClC_6_H_3_	Ph	Me	**3aq**	55
18	2,3-ClC_6_H_3_	Ph	Me	**3ar**	58
19	2-Cl-4-FC_6_H_3_	Ph	Me	**3as**	56
20	2,4-FC_6_H_3_	Ph	Me	**3at**	57
21	2-naphthyl	Ph	Me	**3au**	90
22	cyclopropyl	Ph	Me	**3av**	83
23	Me	Ph	Me	**3aw**	76
24	2-thienyl	Ph	Me	**3ax**	84
25	Ph	4-MeC_6_H_4_	Me	**3ba**	88
26	Ph	3-MeC_6_H_4_	Me	**3bb**	84
27	Ph	4-*^n^*ButylC_6_H_4_	Me	**3bc**	85
28	Ph	4-OMeC_6_H_4_	Me	**3bd**	95
29	Ph	3-OMeC_6_H_4_	Me	**3be**	92
30	Ph	4-FC_6_H_4_	Me	**3bf**	74
31	Ph	4-ClC_6_H_4_	Me	**3bg**	75
32	Ph	2-ClC_6_H_4_	Me	**3bh**	60
33	Ph	4-BrC_6_H_4_	Me	**3bi**	78
34	Ph	4-COOMeC_6_H_4_	Me	**3bj**	70
35	Ph	cyclopropyl	Me	**3bk**	87
36	Ph	*n*-hexyl	Me	**3bl**	82
37	Ph	2-thienyl	Me	**3bm**	80
38	4-MeC_6_H_4_	4-OMeC_6_H_4_	Me	**3ca**	96
39	4-MeC_6_H_4_	4-*^n^*ButylC_6_H_4_	Me	**3cb**	90
40	4-OMeC_6_H_4_	4-OMeC_6_H_4_	Me	**3cc**	98
41	2-FC_6_H_4_	2-ClC_6_H_4_	Me	**3cd**	64
42	2-BrC_6_H_4_	4-OMeC_6_H_4_	Me	**3ce**	68
43	4-cynaoC_6_H_4_	4-OMeC_6_H_4_	Me	**3cf**	70
44	2,4-FC_6_H_3_	2-ClC_6_H_4_	Me	**3cg**	53
45	2,3-ClC_6_H_3_	2-ClC_6_H_4_	Me	**3ch**	59
46	2-naphthyl	4-*^n^*ButylC_6_H_4_	Me	**3ci**	88
47	2-naphthyl	2-ClC_6_H_4_	Me	**3cj**	80
48	2-BrC_6_H_4_	cyclopropyl	Me	**3ck**	60
49	3-MeC_6_H_4_	Ph	Ph	**3da**	53
50	3-MeC_6_H_4_	Ph	Bn	**3db**	69
51	3-MeC_6_H_4_	Ph	H	**3dc**	90

*^a^* Reaction conditions: the reactions were performed in a round-bottomed flask on a 0.6 mmol scale of **1**, 0.5 mmol scale of **2** and 0.6 mmol scale of K_2_CO_3_ in 1,4-dioxane (2 mL) at 100 °C for 8 h. *^b^* Isolated yields.

## Data Availability

The data presented in this study are available upon request from the corresponding author.

## Supplementary Materials

The following supporting information can be downloaded at: https://www.mdpi.com/article/10.3390/molecules29122792/s1, Copies of NMR spectra for compounds **3aa**–**3dc** (page 2). Insecticidal properties study methods (page 58).
