# Supplementary material for: Metal-Free Cascade Formation of C–C and C–N Bond for the Construction of 3-Cyano-2-Pyridones with Insecticidal Properties
Source: Molecules. 2024 Jun 12;29(12):2792. doi: 10.3390/molecules29122792 (PMC11206961; doi:10.3390/molecules29122792)
Supplement: Supplementary file 1 [file molecules-29-02792-s001.zip › molecules-3017776-supplementary.pdf]

Metal-free Cascade Formation of C–C and C–N Bond for the Construction of 3-Cyano-2-Pyridones with Insecticidal Properties

Yao Tang, Nvjiang Wu, Junyu Xu, Xiaopo Zhang,* Youbin Li* and Xuesong Wang*

Engineering Research Center of Tropical Medicine Innovation and Transformation of Ministry of Education, International Joint Research Center of Human-machine Intelligent Collaborative for Tumor Precision Diagnosis and Treatment of Hainan Province, Hainan Key Laboratory for Research and Development of Tropical Herbs, Haikou Key Laboratory of Li Nationality Medicine, School of Pharmacy, Academy of Medical Sciences, Hainan Medical University, Haikou 571199, China; ty2024abcd@sina.com (Y. T.); healthyhu@163.com (N. W.); xujy201309@hainmc.edu.cn (J. X.)

* Correspondence: z_xp1412@163.com (X. Z.); liyoubinli@sohu.com (Y. L.) and hy0207110@hainmc.edu.cn (X. W.); Tel.: +86-0898-66890907 (X. W.)

Context

^{1}H NMR, ^{13}C NMR, ^{19}F NMR spectra of products…………………………………………..S2-57

Insecticidal Properties Study Methods……………………………………..……..……..S58-61

^{1}H NMR, ^{13}C NMR and ^{19}F NMR of products

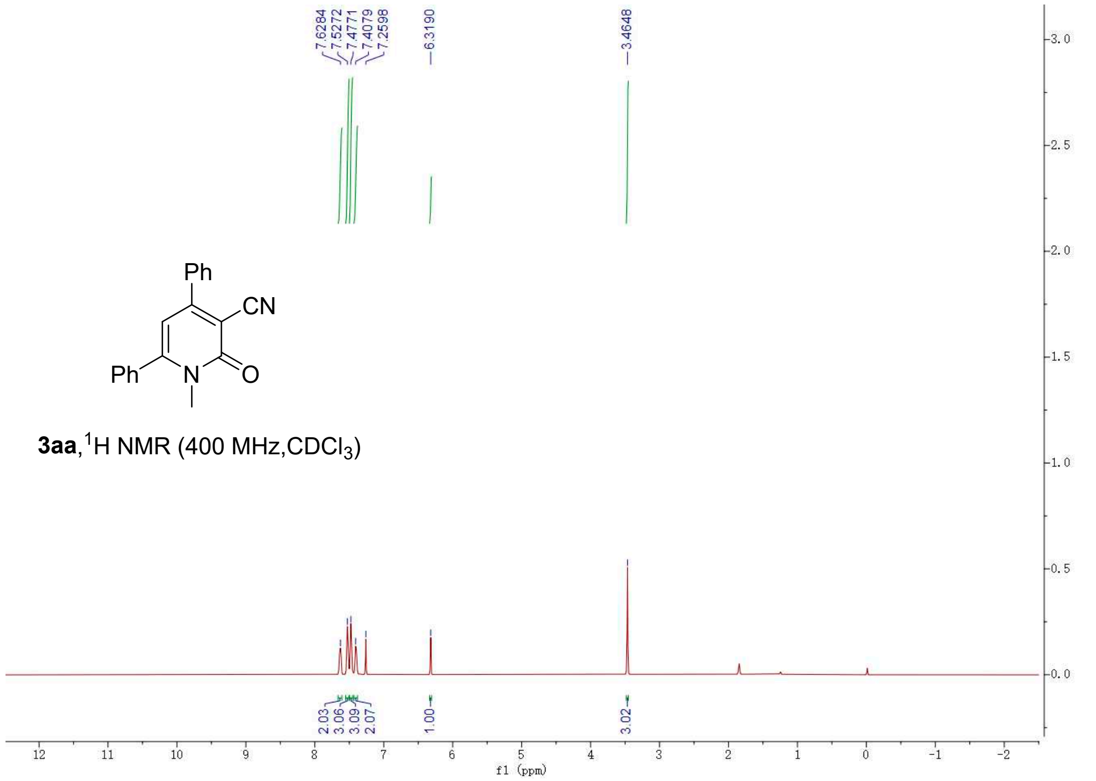

3aa, ^{1}H NMR (400 MHz, $CDCl_3$)

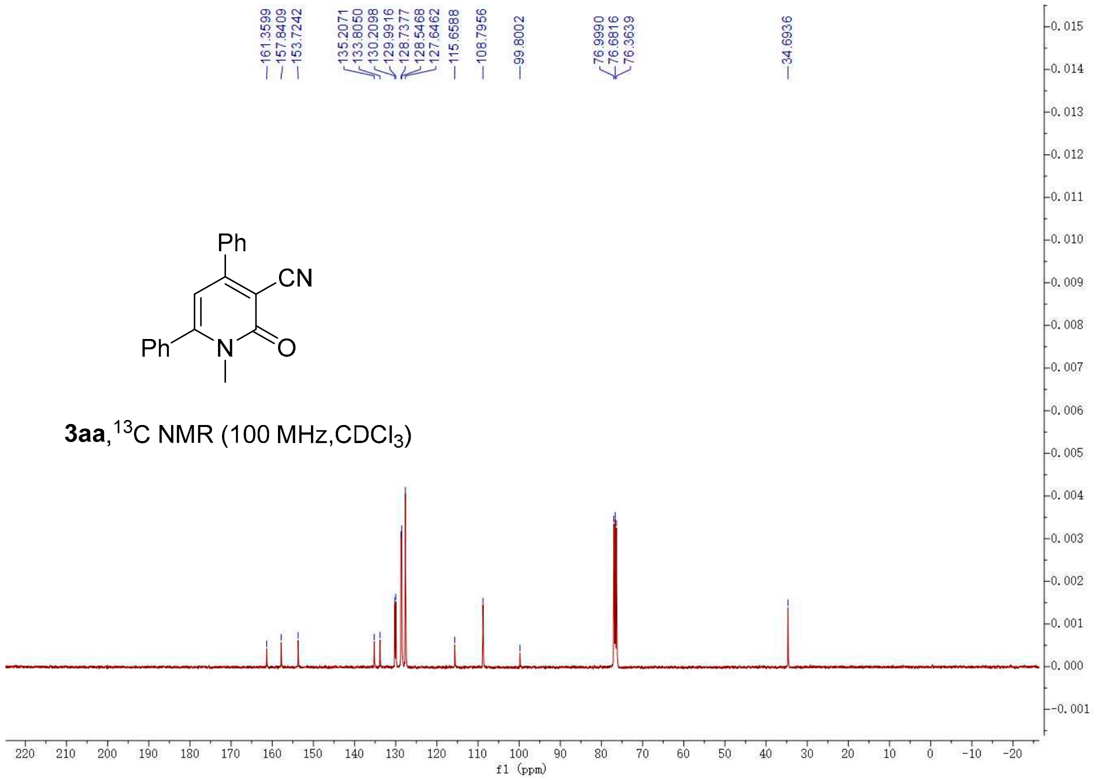

3aa, ^{13}C NMR (100 MHz, $CDCl_3$)

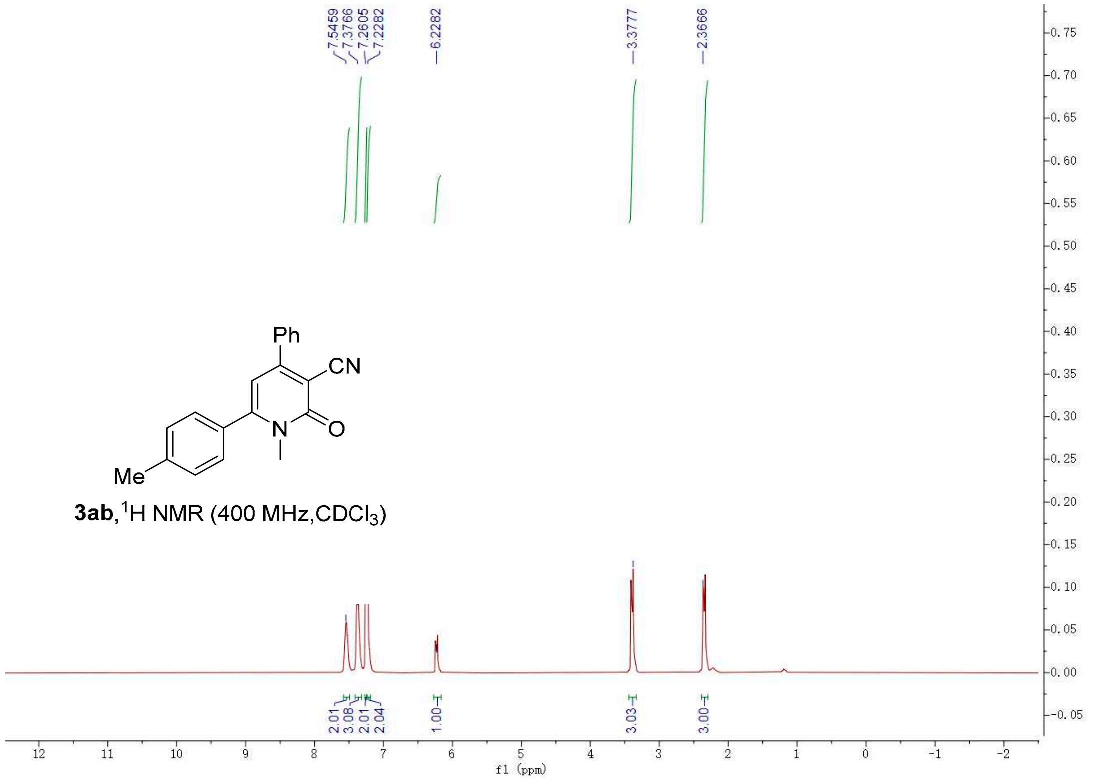
7.5459
7.3766
7.2605
7.2282
6.2282
3.3777
2.3666
Ph
CN
N
O
Me
3ab, ¹H NMR (400 MHz,CDCl₃)
2.01
3.08
2.01
2.04
1.00
3.03
3.00
f1 (ppm)

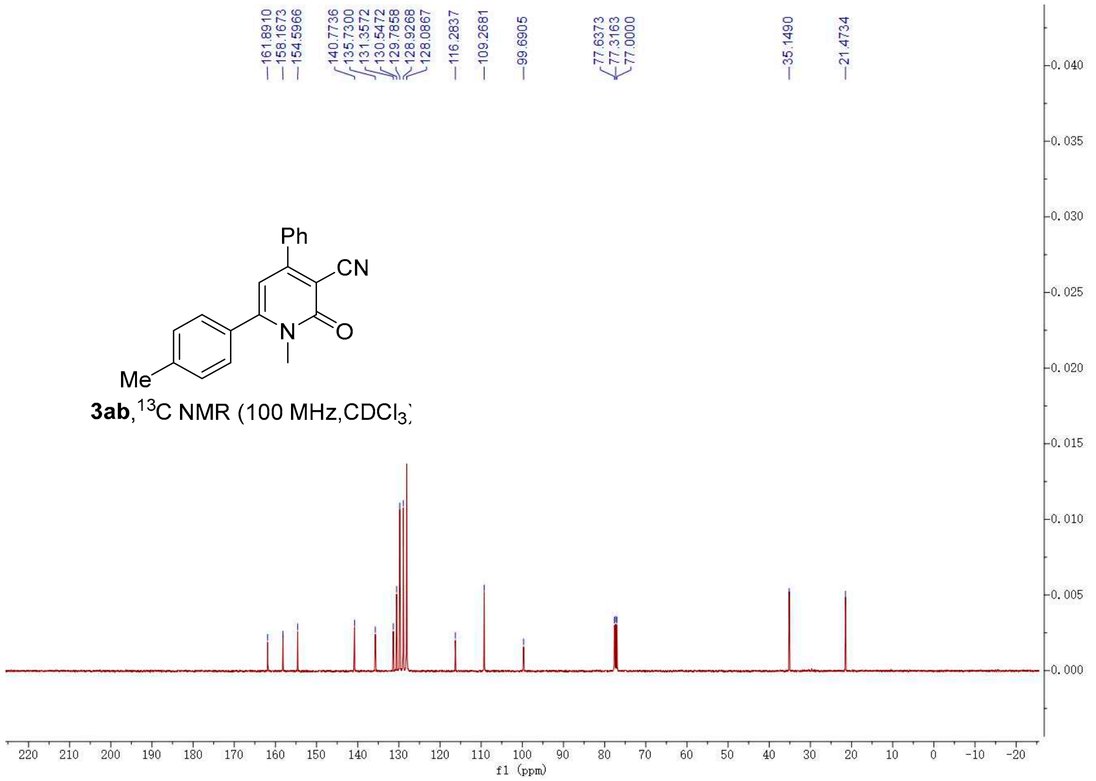
161.8910
158.1673
154.5966
140.7736
135.7300
131.3572
130.5472
129.7858
128.9268
128.0867
116.2837
109.2681
99.6905
77.6373
77.3163
77.0000
35.1490
21.4734
Ph
CN
N
O
Me
3ab, ¹³C NMR (100 MHz,CDCl₃)
f1 (ppm)

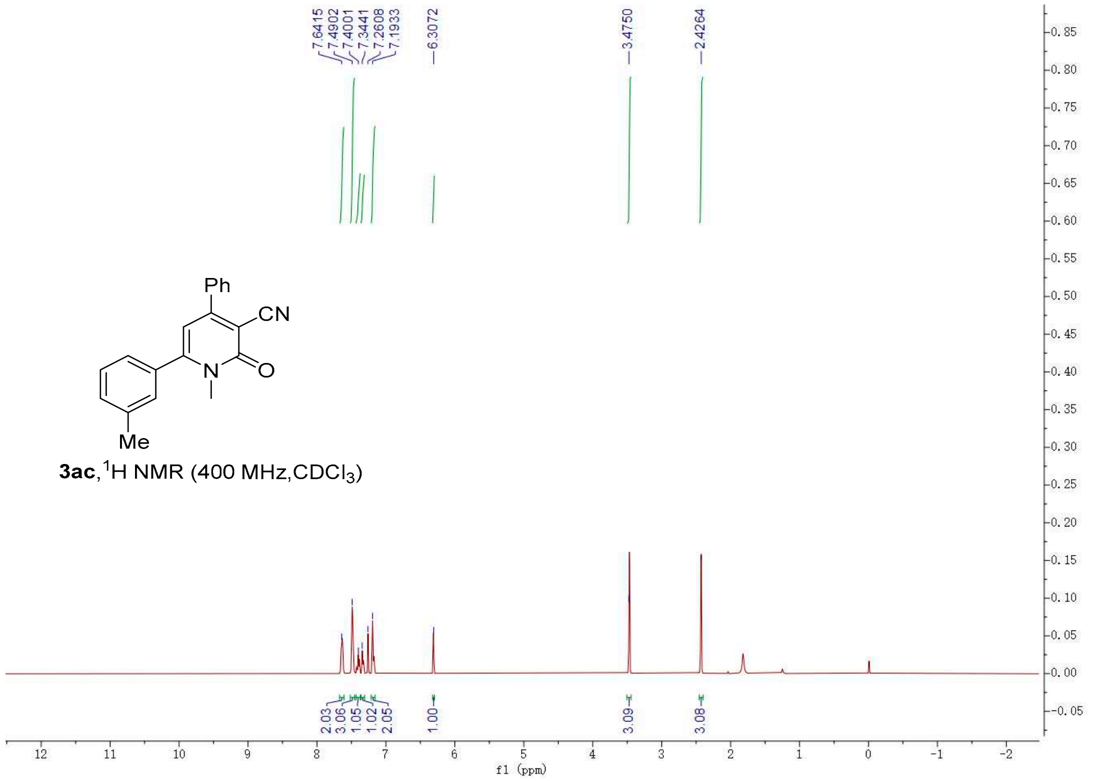
Ph
CN
N
O
Me
3ac, 1H NMR (400 MHz,CDCl3)
f1 (ppm)

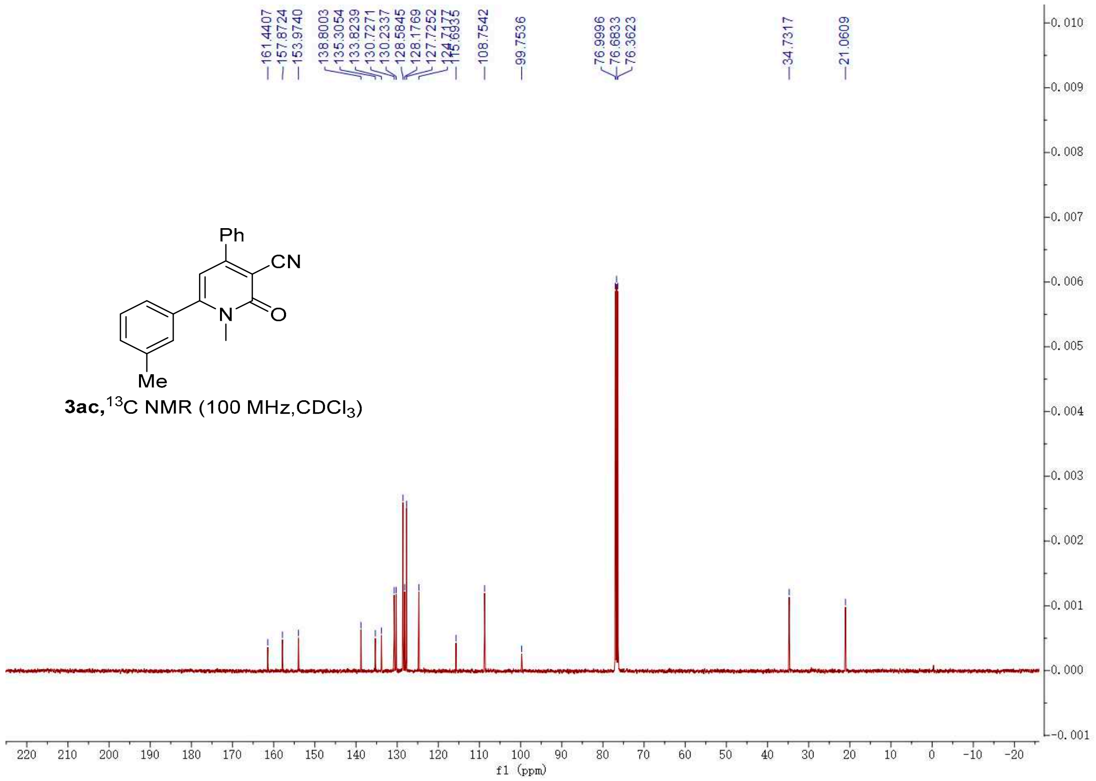
Ph
CN
N
O
Me
3ac, 13C NMR (100 MHz,CDCl3)
f1 (ppm)

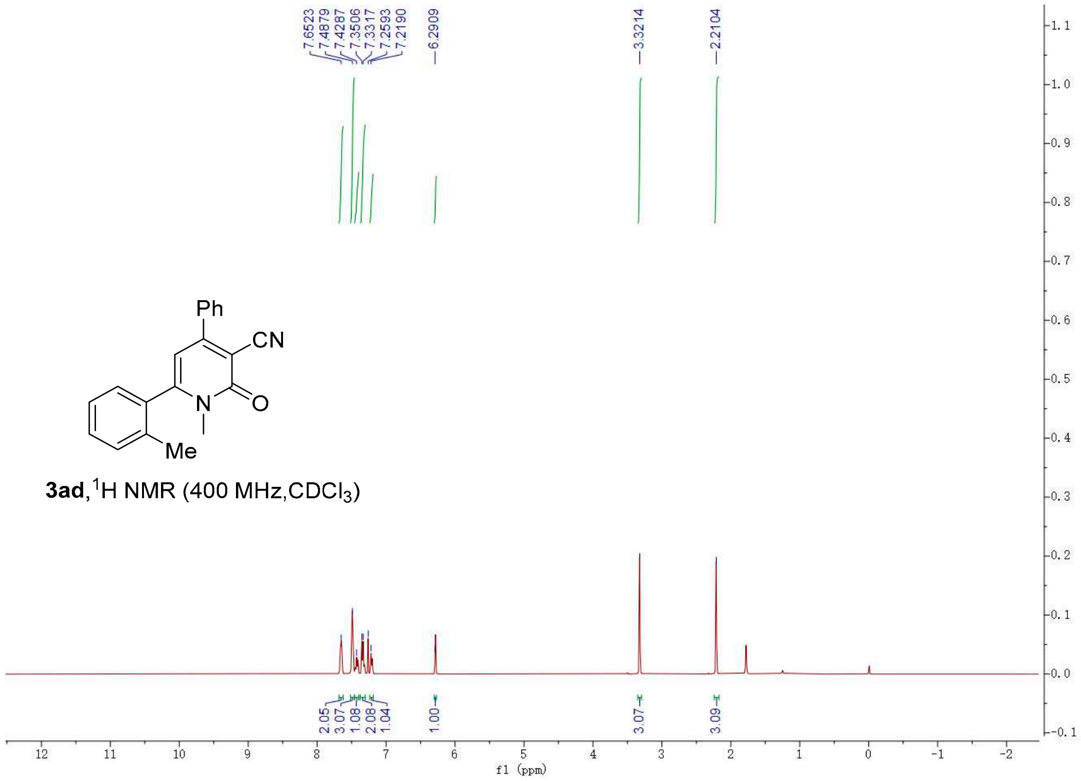

3ad, ^{1}H NMR (400 MHz, $CDCl_3$)

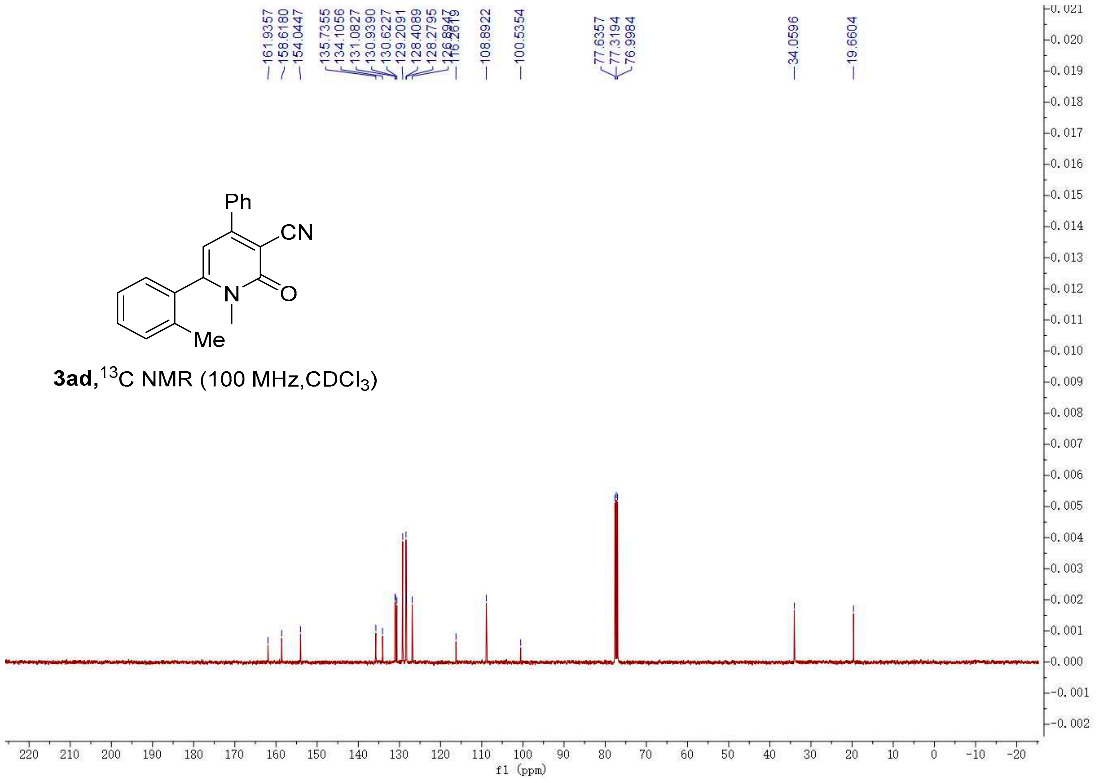

3ad, ^{13}C NMR (100 MHz, $CDCl_3$)

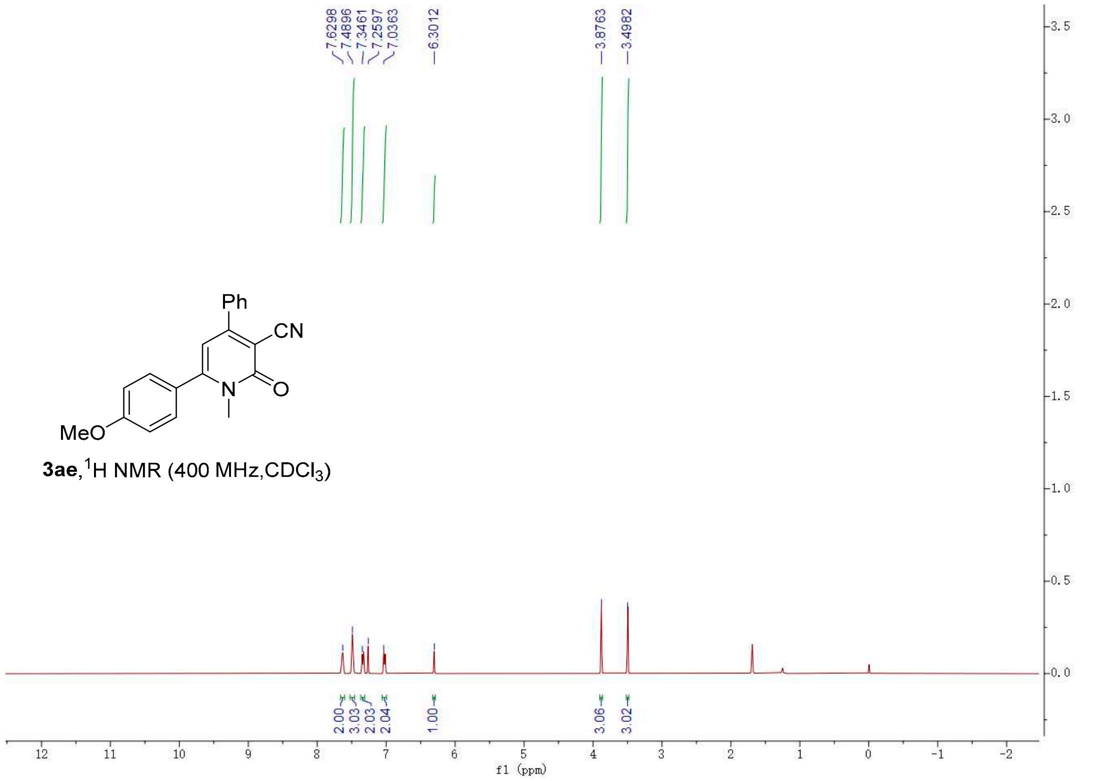

3ae, ^{1}H NMR (400 MHz, $CDCl_3$)

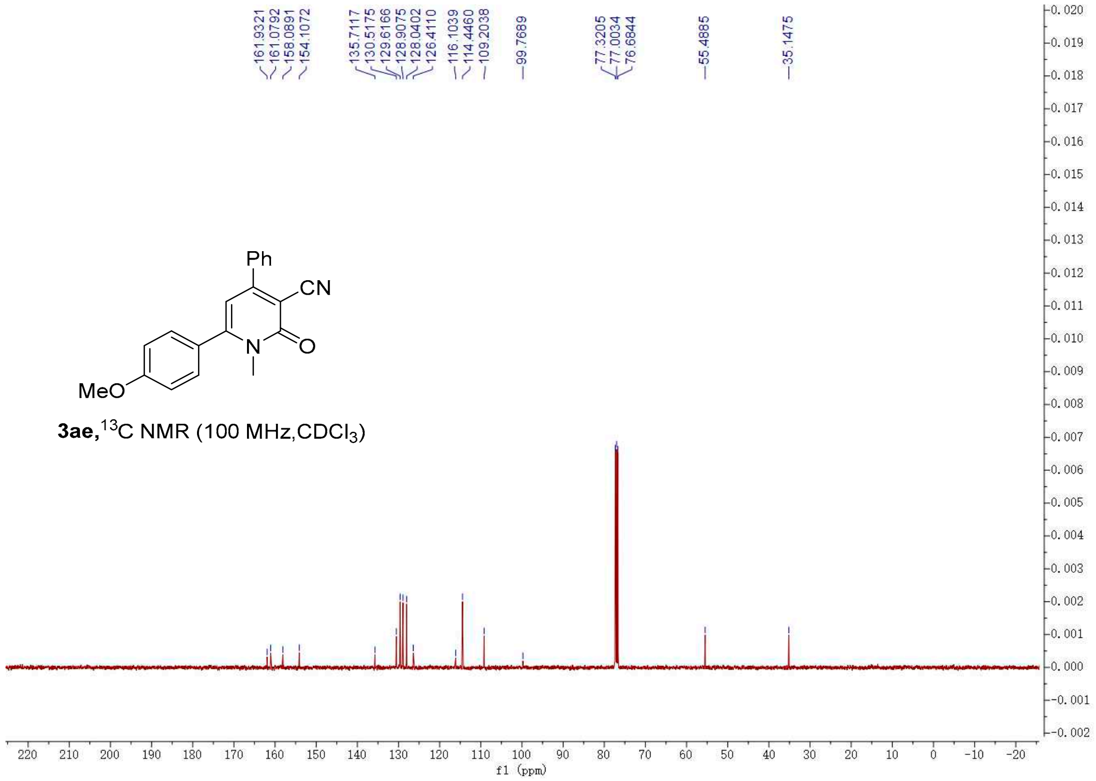

3ae, ^{13}C NMR (100 MHz, $CDCl_3$)

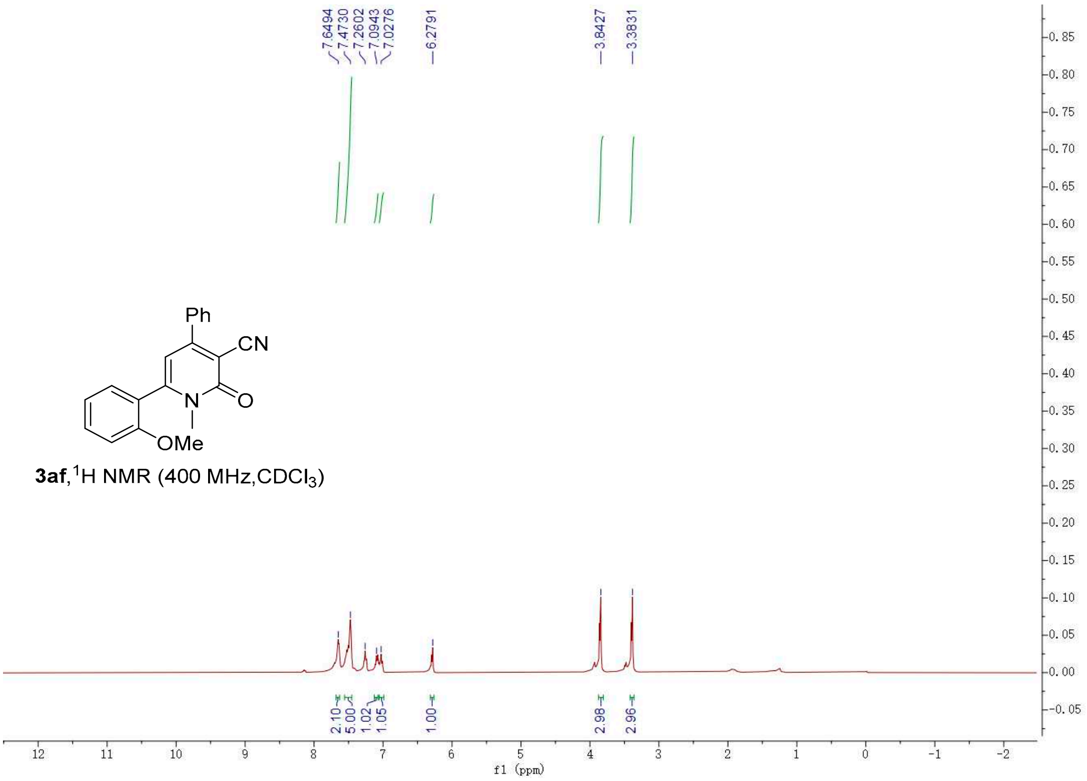
7.6494
7.4730
7.2602
7.0943
7.0276
6.2791
3.8427
3.3831
Ph
CN
N
O
OMe
3af,1H NMR (400 MHz,CDCl3)
2.10
5.00
1.02
1.05
1.00
2.98
2.96
f1 (ppm)

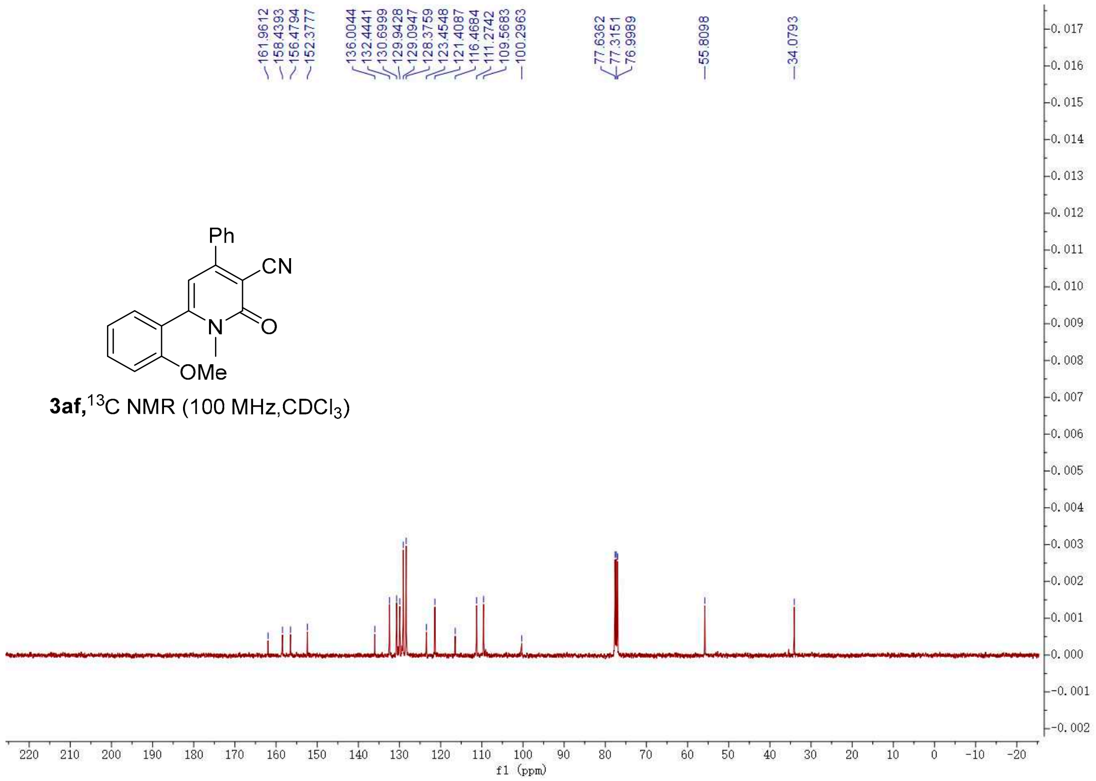
161.9612
158.4393
156.4794
152.3777
136.0044
132.4441
130.6999
129.9428
129.0947
128.3759
123.4548
121.4087
116.4684
111.2742
109.5683
100.2963
77.6362
77.3151
76.9989
55.8098
34.0793
Ph
CN
N
O
OMe
3af,13C NMR (100 MHz,CDCl3)
f1 (ppm)

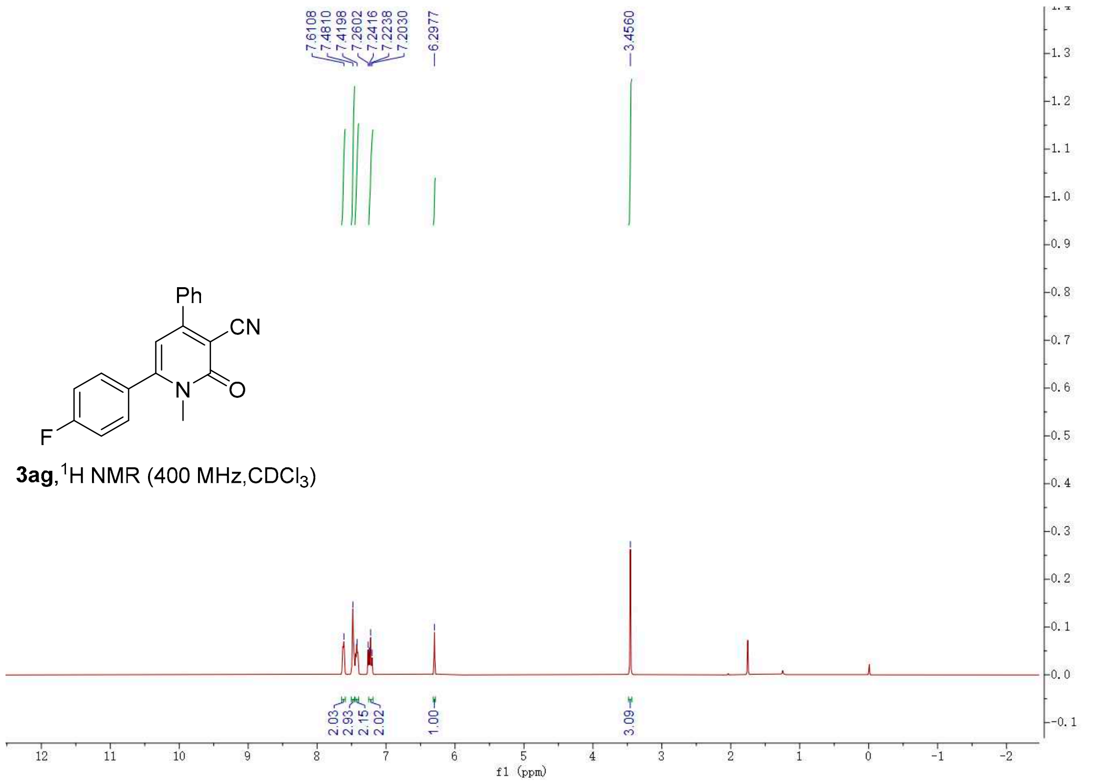

3ag,^{1}H NMR (400 MHz,$CDCl_3$)

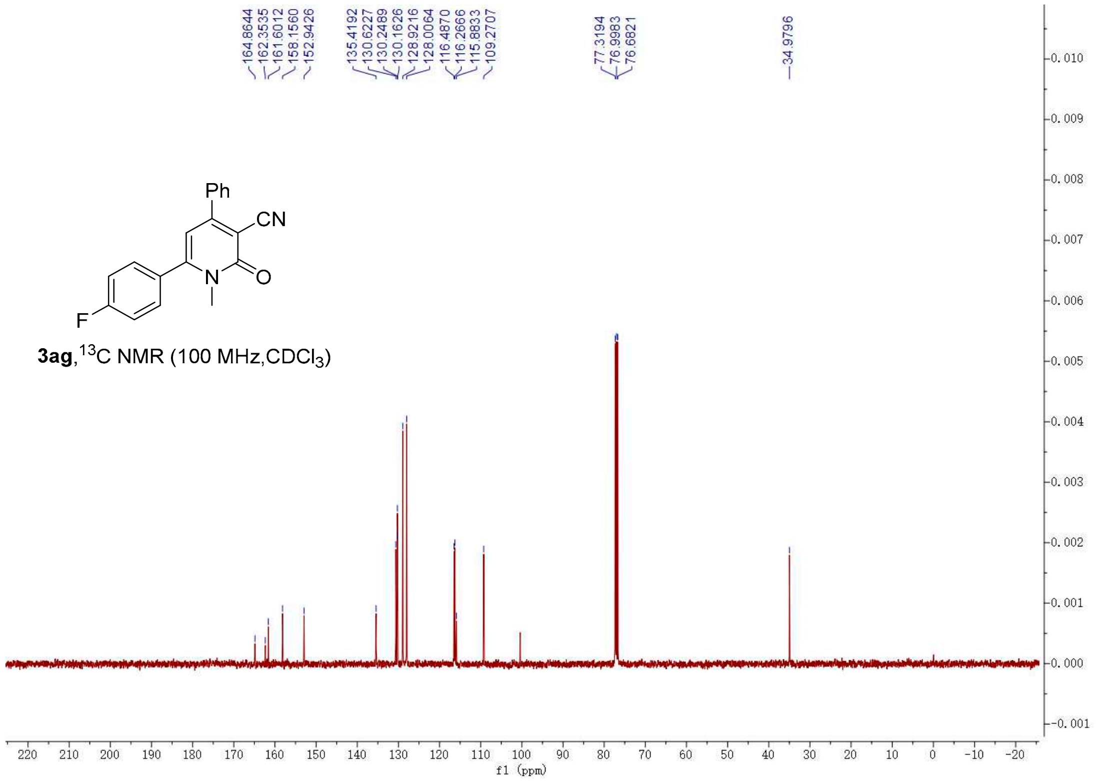

3ag,^{13}C NMR (100 MHz,$CDCl_3$)

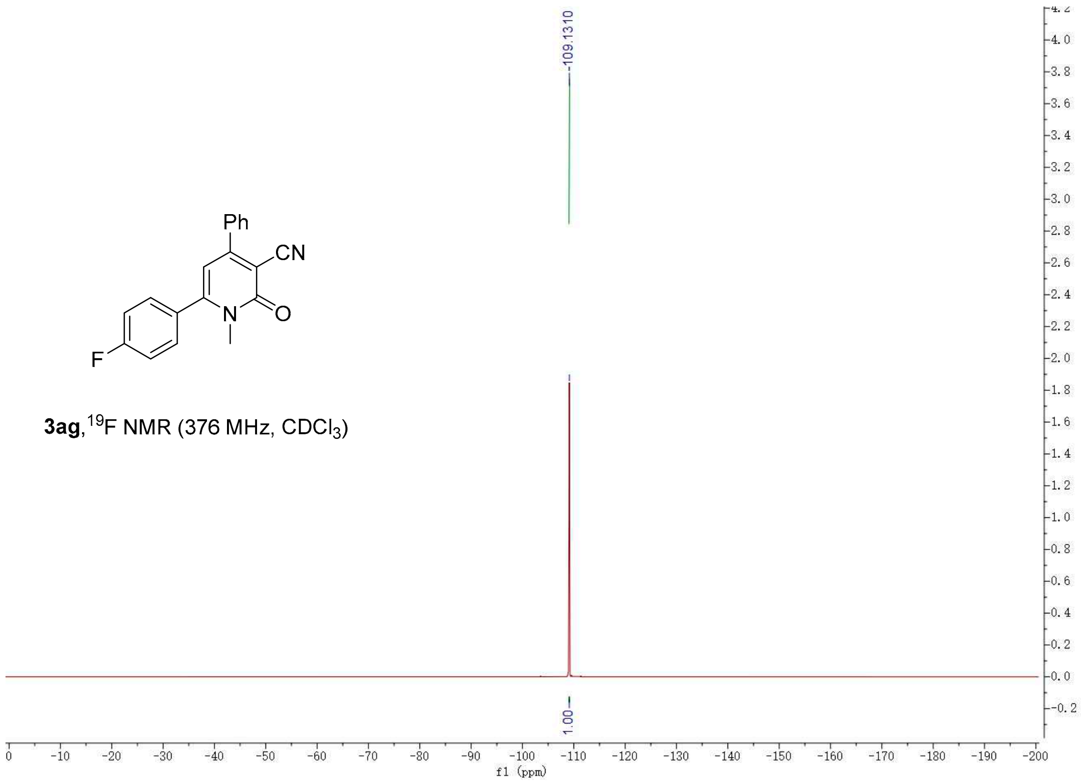

3ag, ^{19}F NMR (376 MHz, $CDCl_3$)

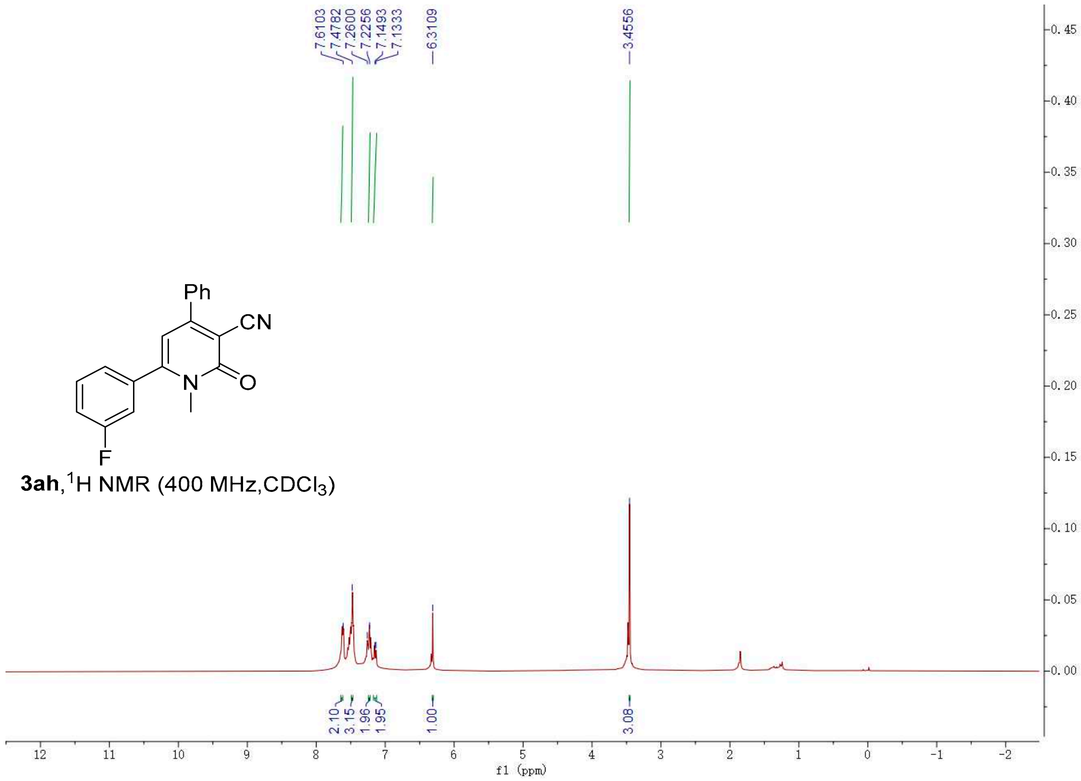

3ah, ^{1}H NMR (400 MHz, $CDCl_3$)

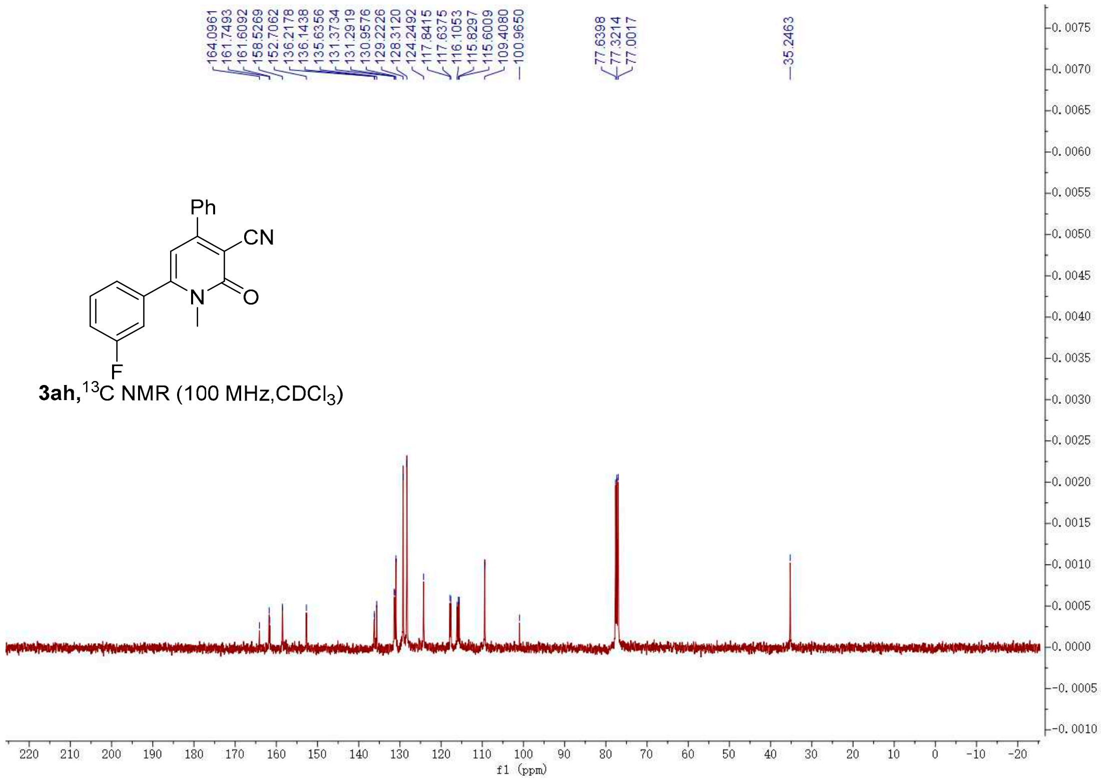

3ah,^{13}C NMR (100 MHz,$CDCl_3$)

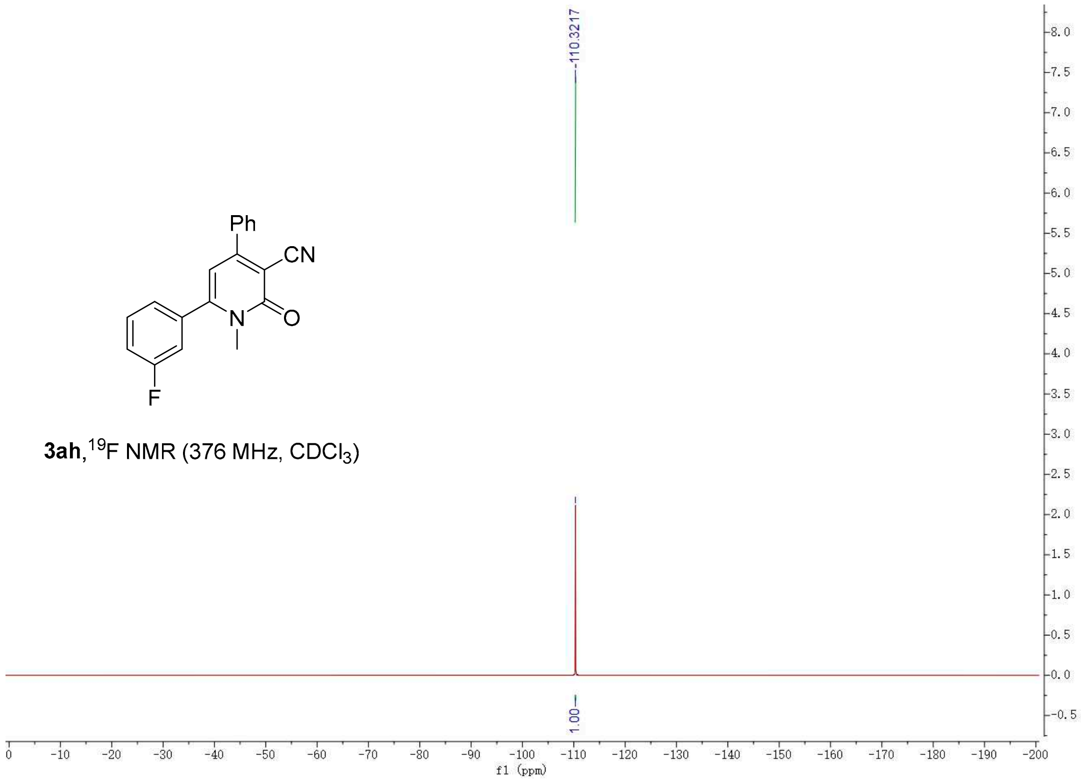

3ah,^{19}F NMR (376 MHz, $CDCl_3$)

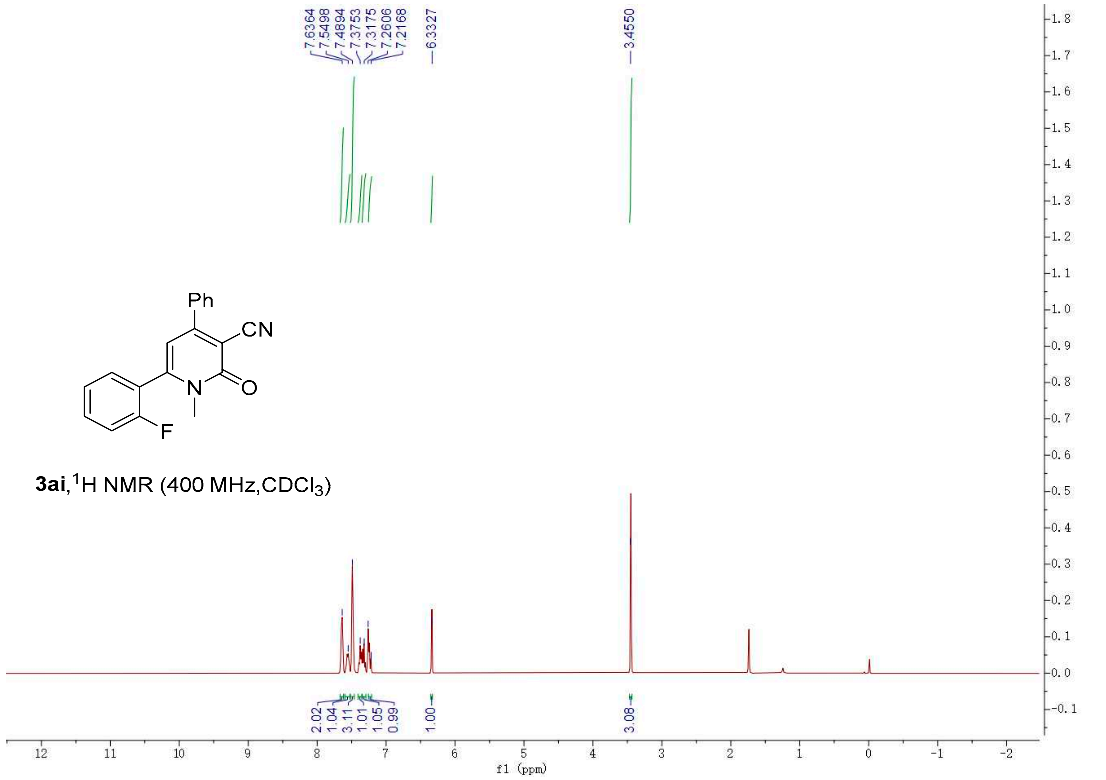

3ai, ^{1}H NMR (400 MHz, $CDCl_3$)

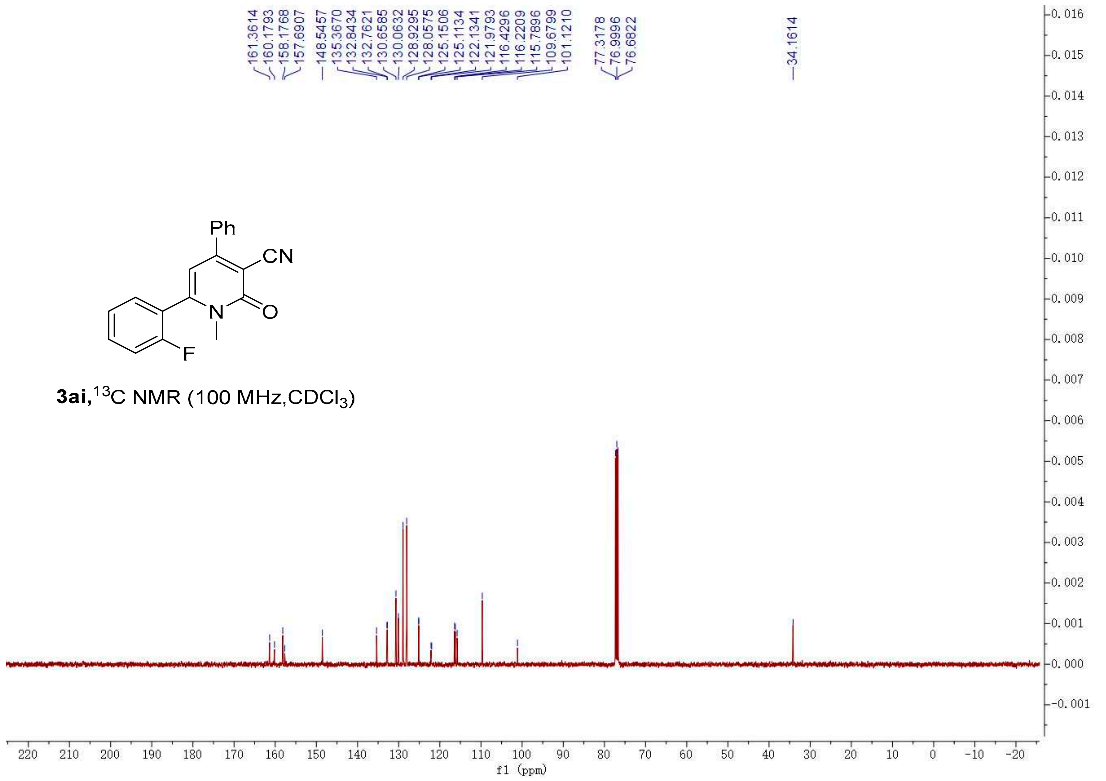

3ai, ^{13}C NMR (100 MHz, $CDCl_3$)

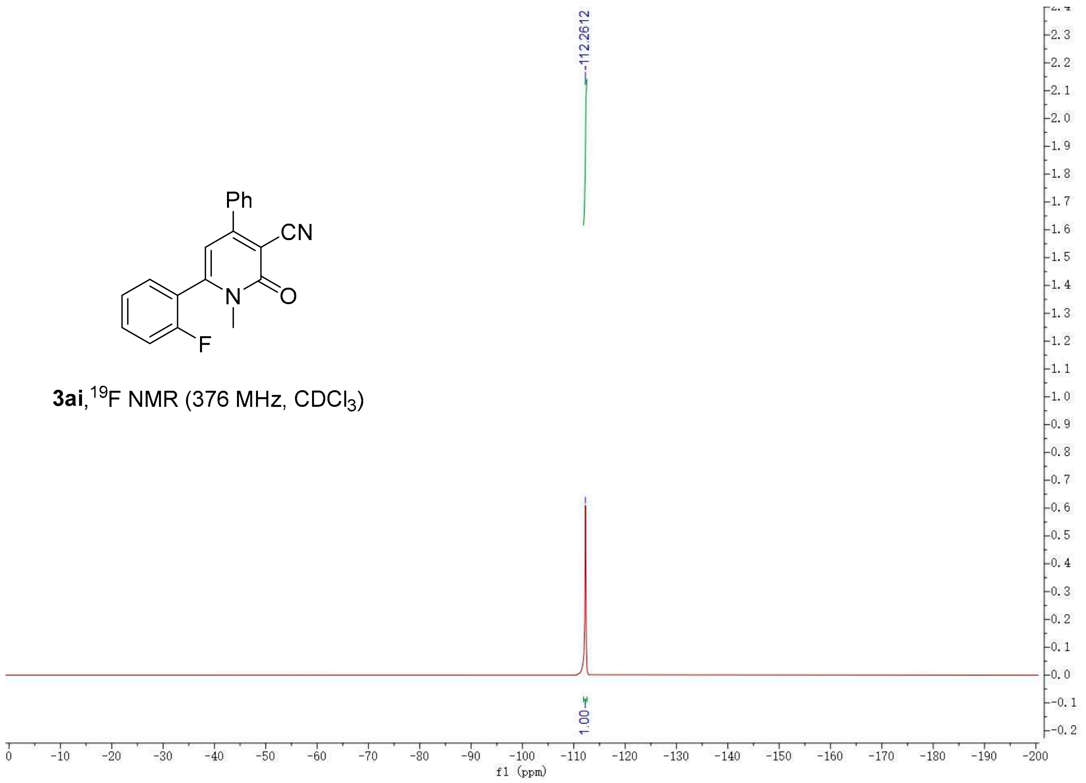

3ai, ^{19}F NMR (376 MHz, $CDCl_3$)

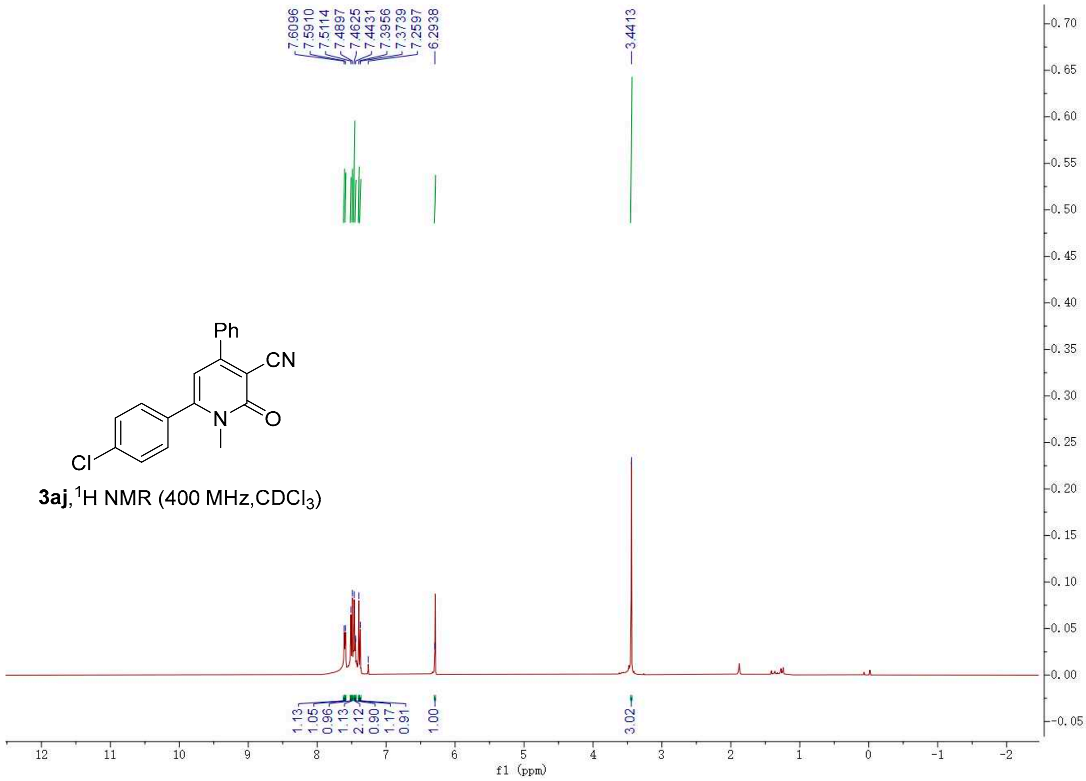

3aj, ^{1}H NMR (400 MHz, $CDCl_3$)

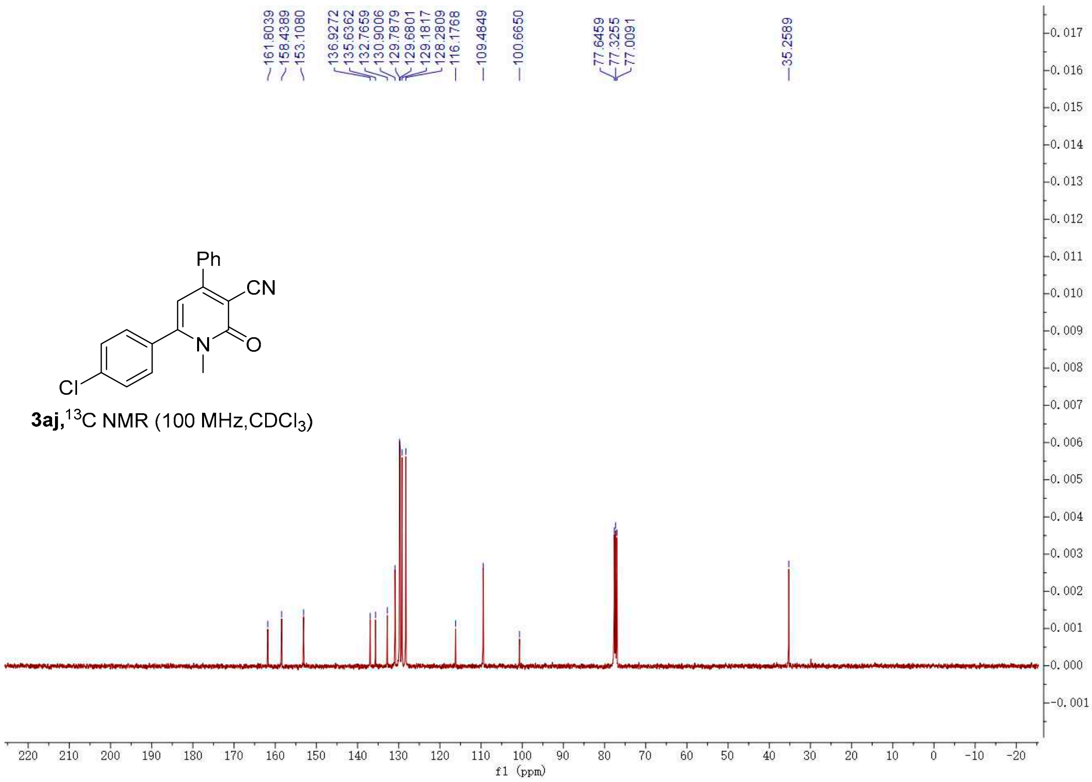

3aj, ^{13}C NMR (100 MHz, $CDCl_3$)

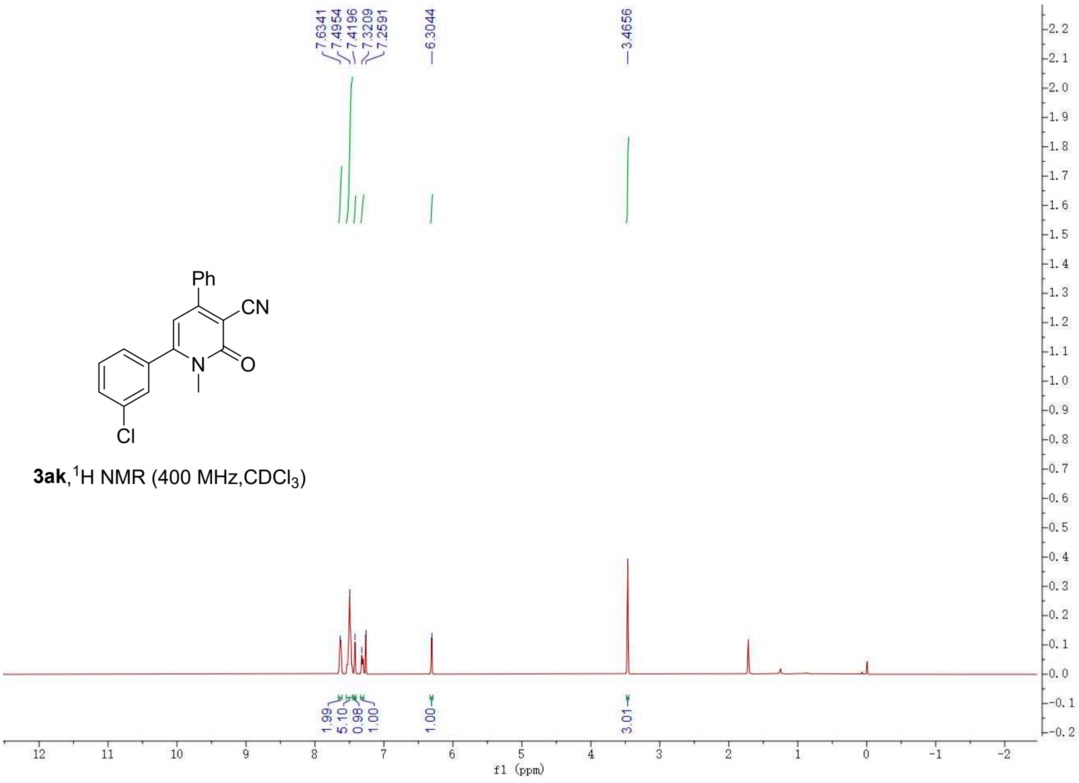

3ak, ^{1}H NMR (400 MHz, $CDCl_3$)

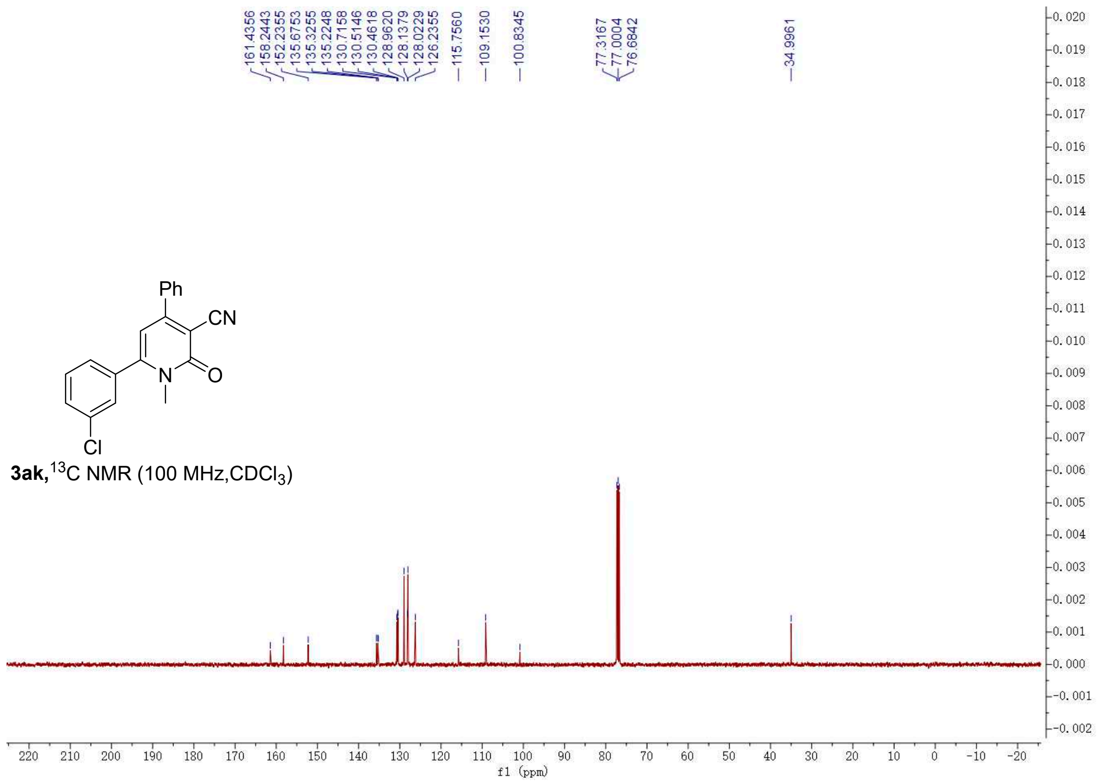

161.4356
158.2443
152.2355
135.6753
135.3255
135.2248
130.7158
130.5146
130.4618
128.9620
128.1379
128.0229
126.2355
115.7560
109.1530
100.8345
77.3167
77.0004
76.6842
34.9961
Ph
CN
N
O
Cl
3ak, ^{13}C NMR (100 MHz,CDCl$_3$)
f1 (ppm)

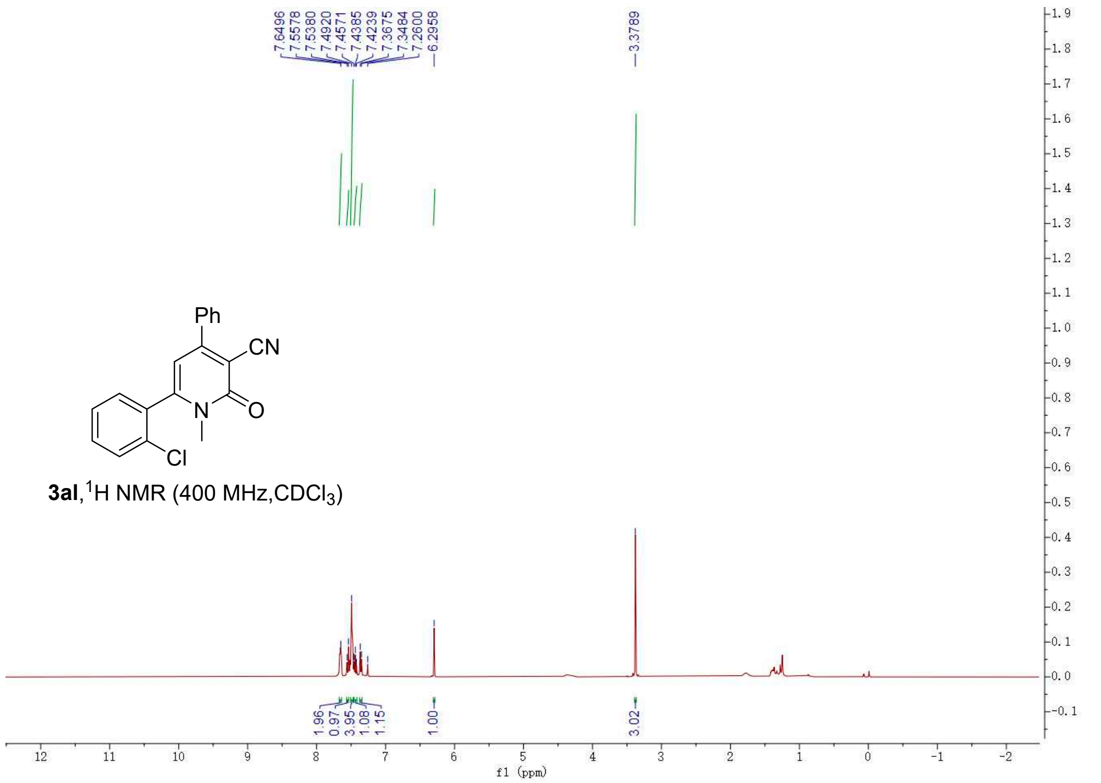

7.6496
7.5578
7.5380
7.4920
7.4571
7.4365
7.4239
7.3675
7.3484
7.2600
6.2958
3.3789
Ph
CN
N
O
Cl
3al, ^{1}H NMR (400 MHz,CDCl$_3$)
1.96
0.97
3.95
1.08
1.15
1.00
3.02
f1 (ppm)

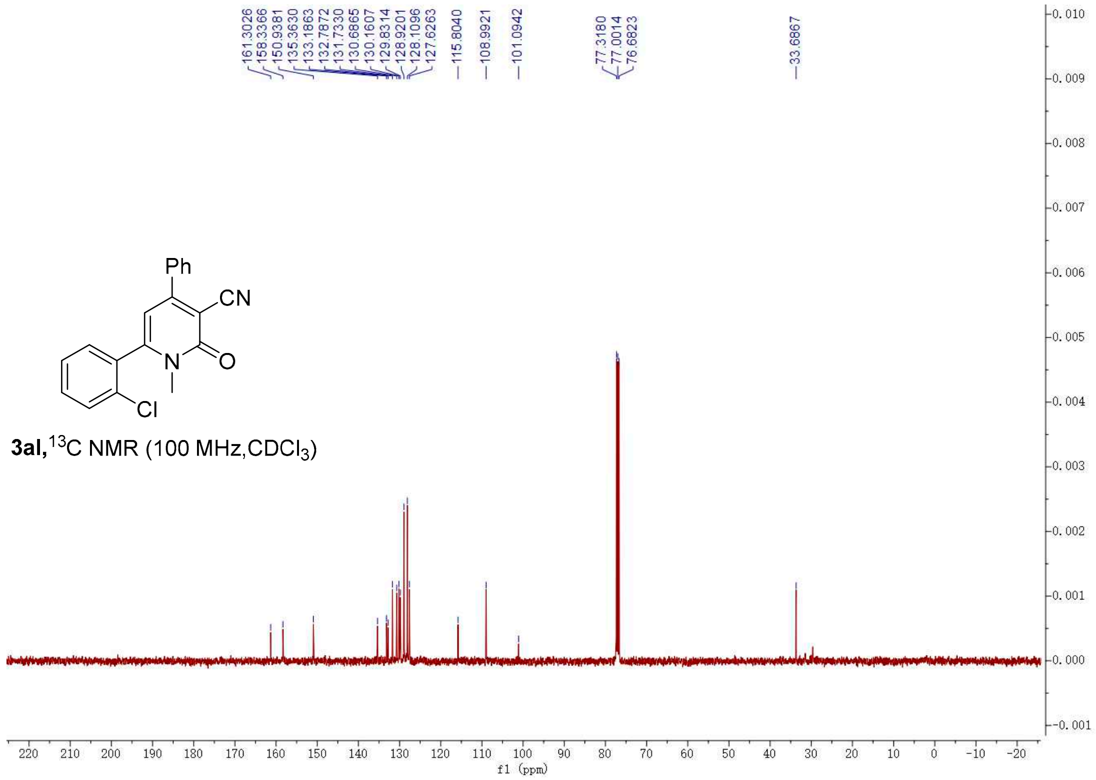

3al,^{13}C NMR (100 MHz,CDCl$_3$)

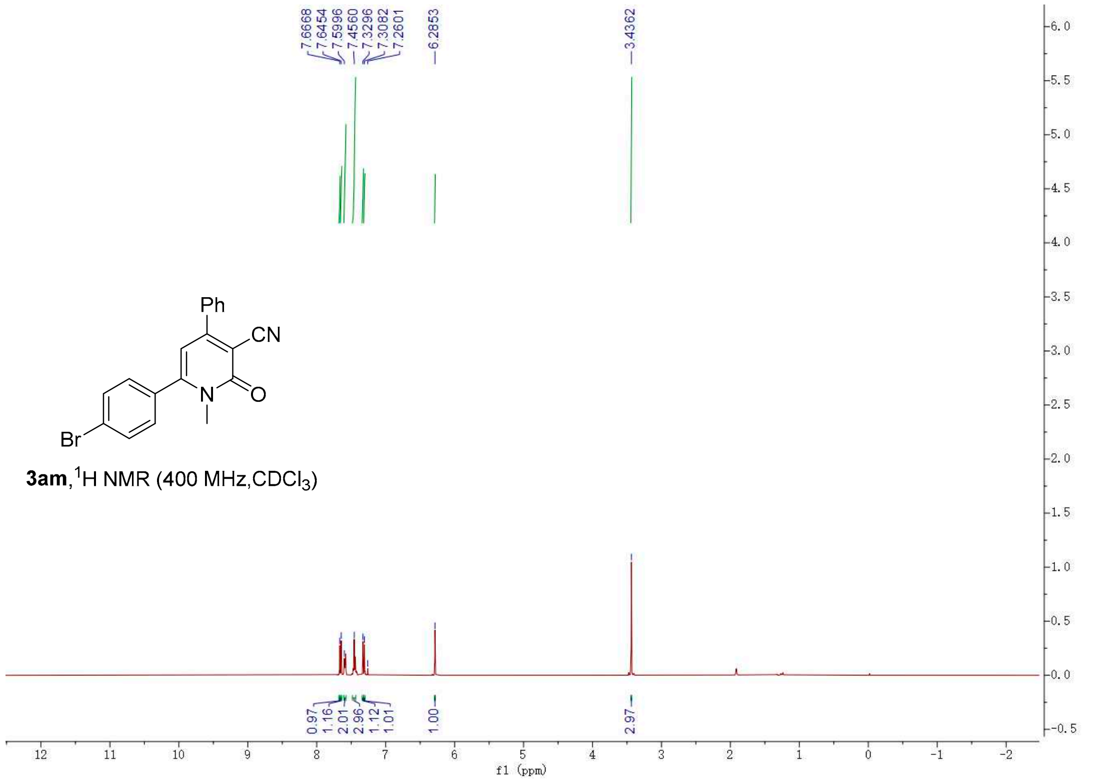

3am,^{1}H NMR (400 MHz,CDCl$_3$)

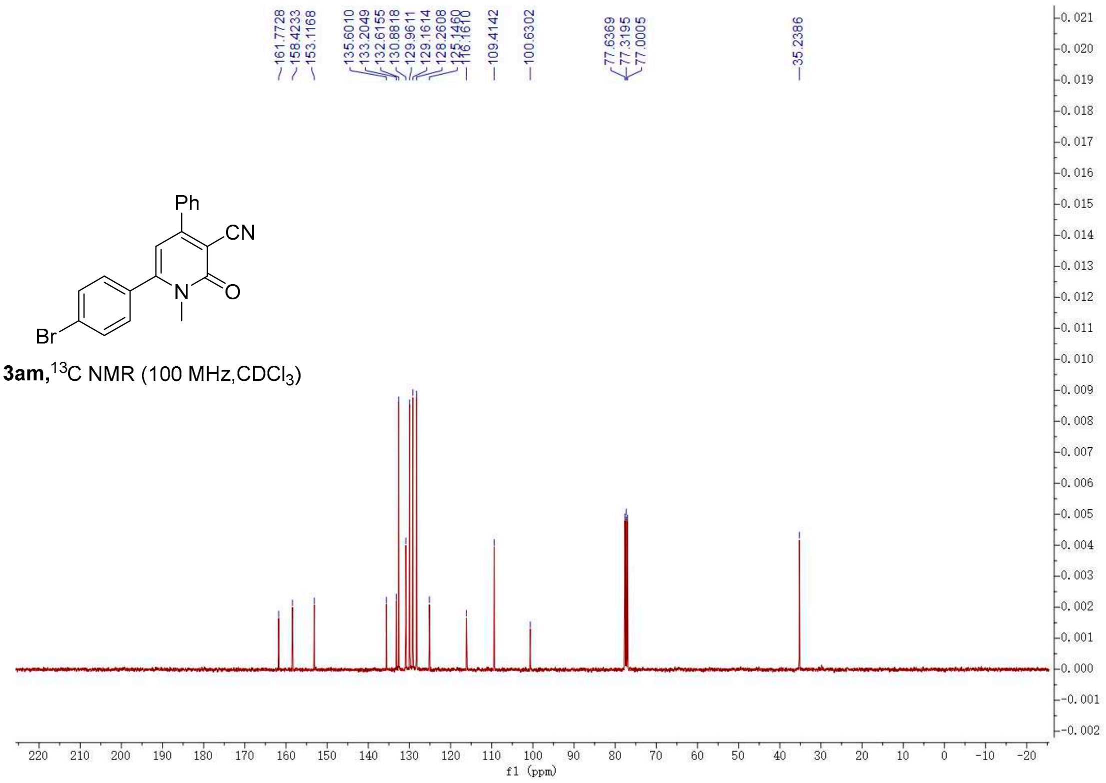

3am,^{13}C NMR (100 MHz,$CDCl_3$)

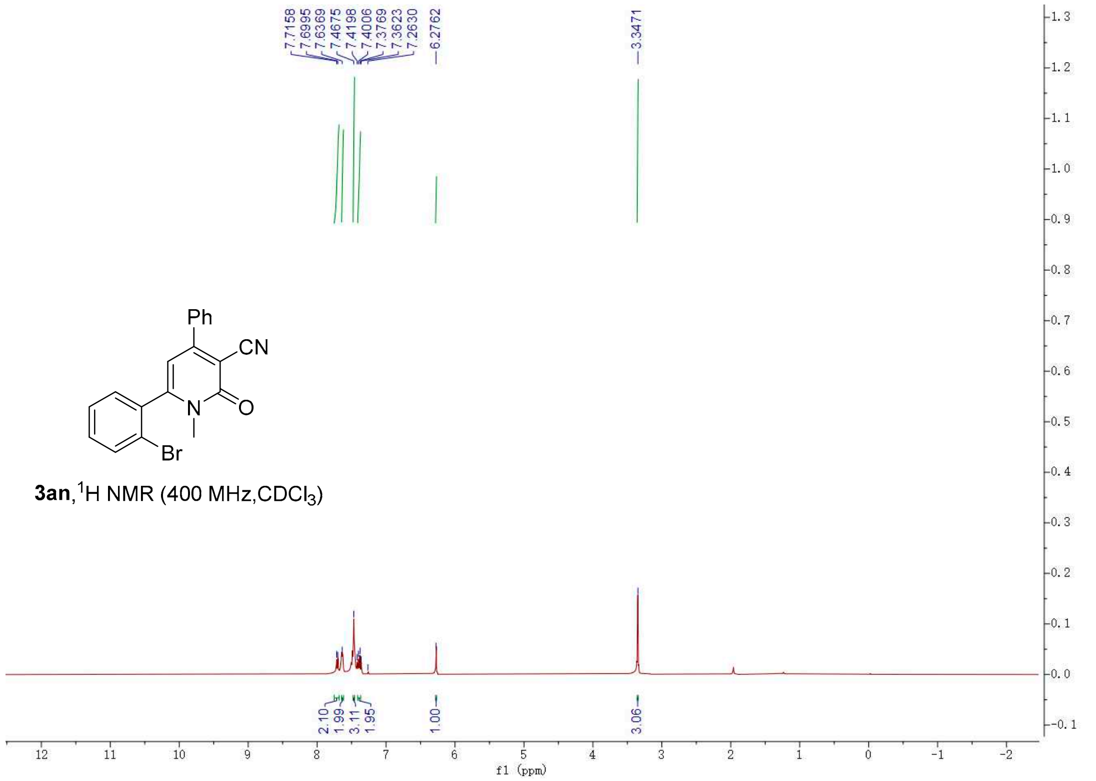

3an,^{1}H NMR (400 MHz,$CDCl_3$)

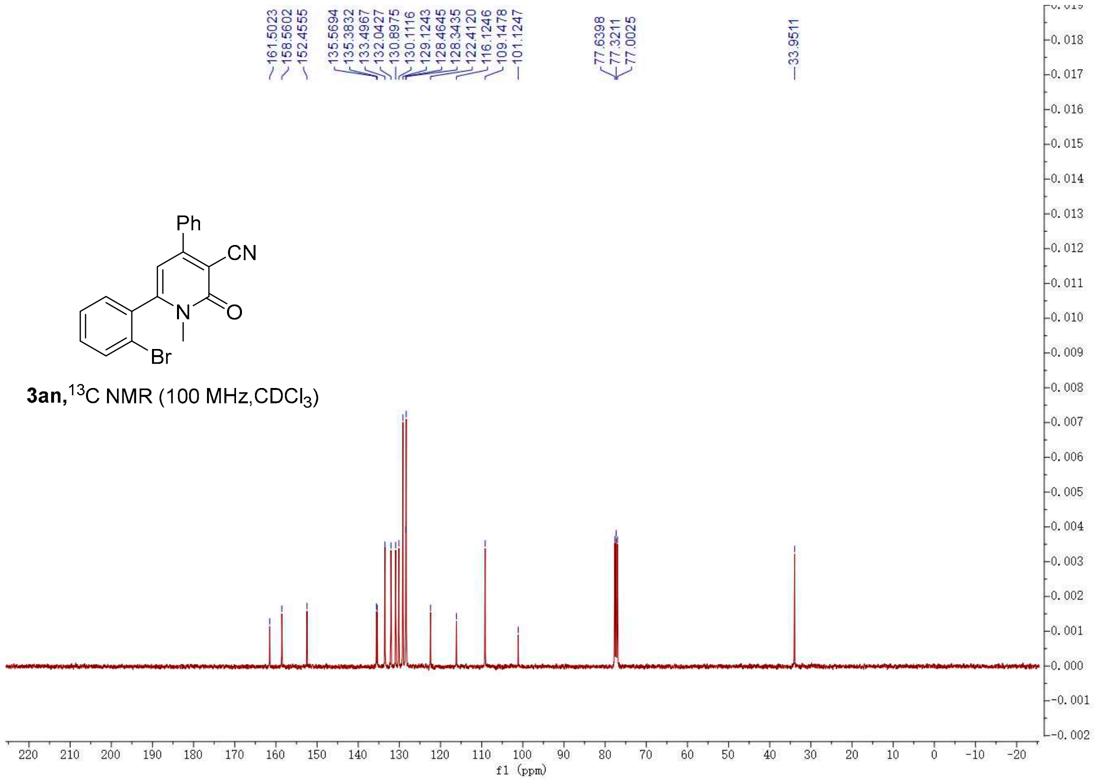

3an, ^{13}C NMR (100 MHz, $CDCl_3$)

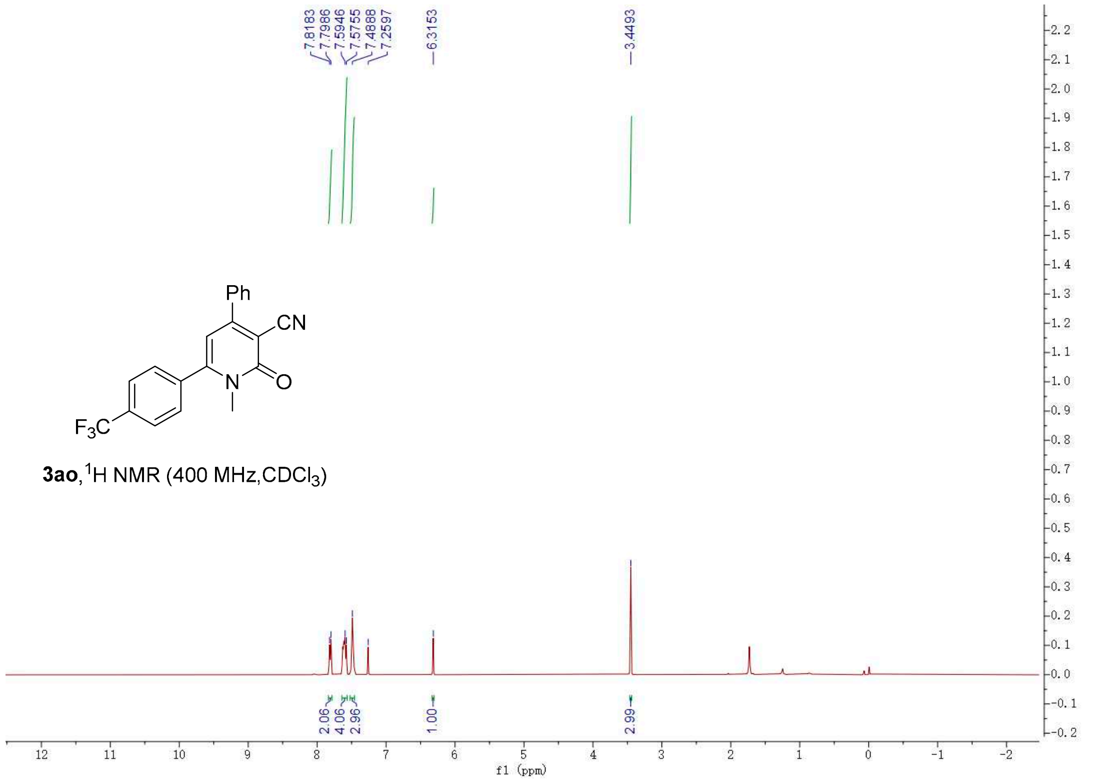

3ao, ^{1}H NMR (400 MHz, $CDCl_3$)

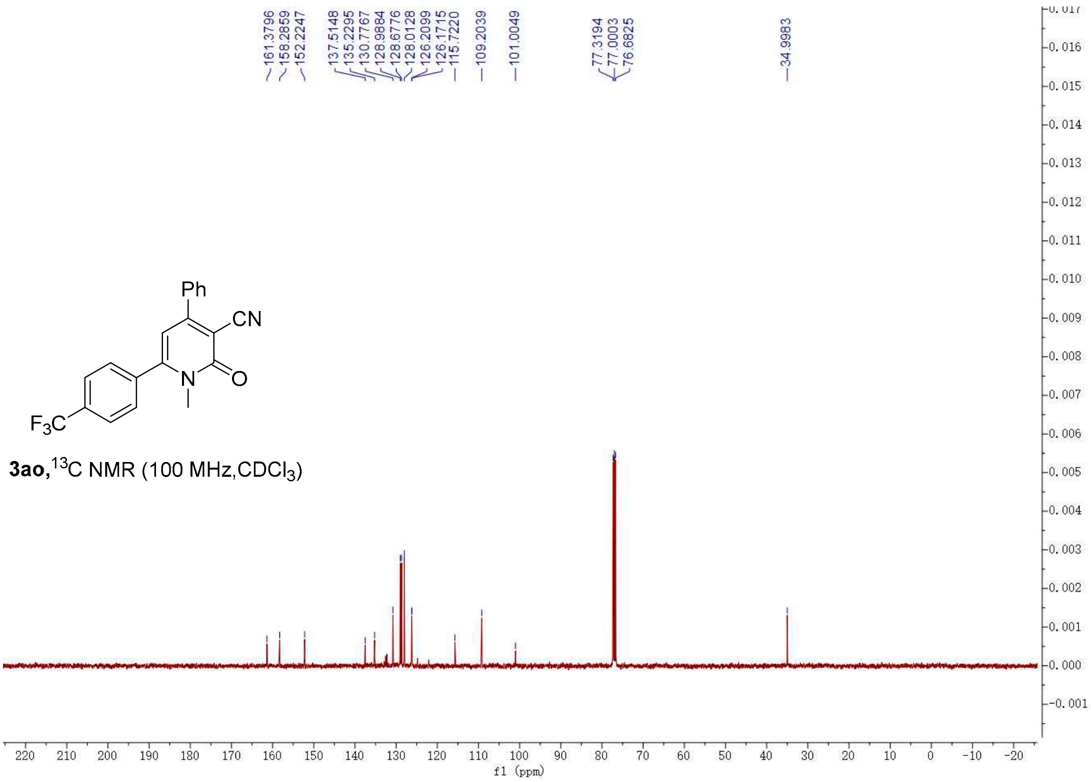

3ao,^{13}C NMR (100 MHz,$CDCl_3$)

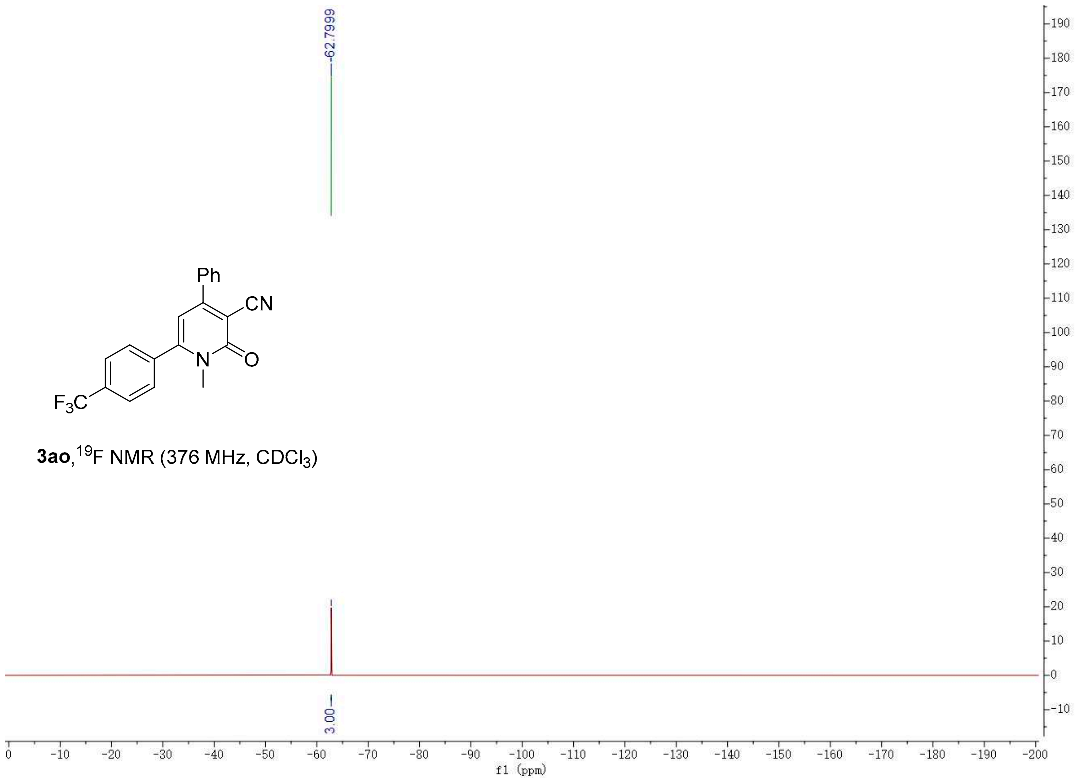

3ao,^{19}F NMR (376 MHz, $CDCl_3$)

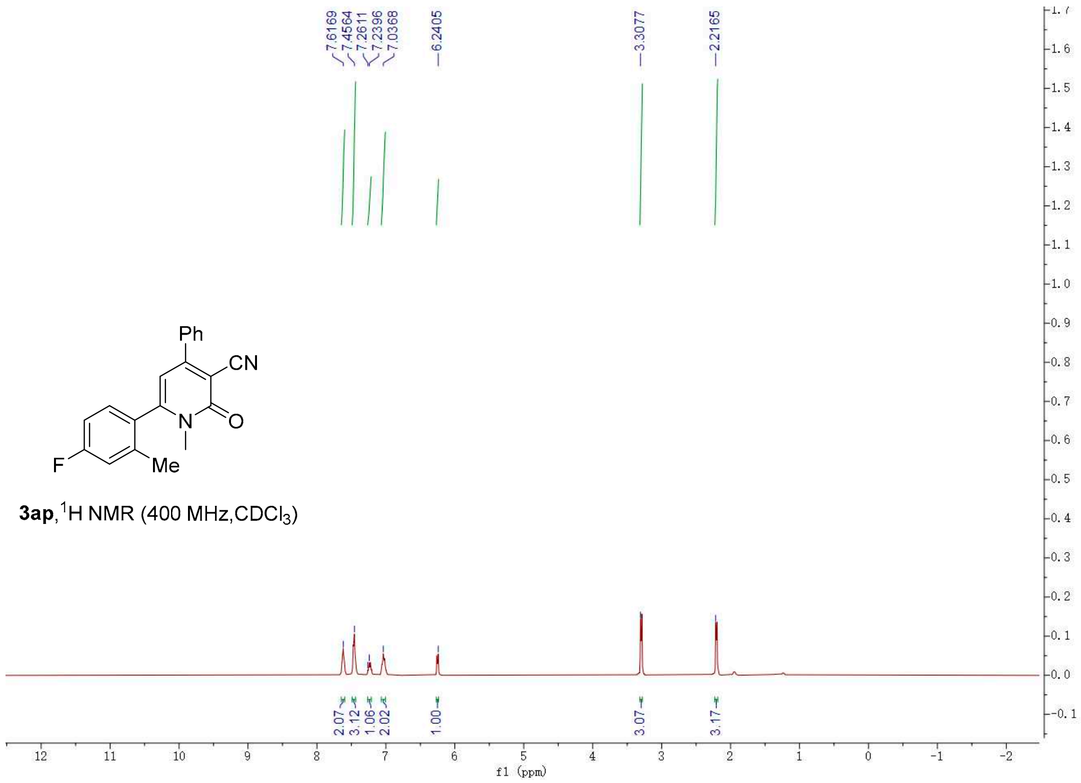

3ap, ^{1}H NMR (400 MHz, $CDCl_3$)

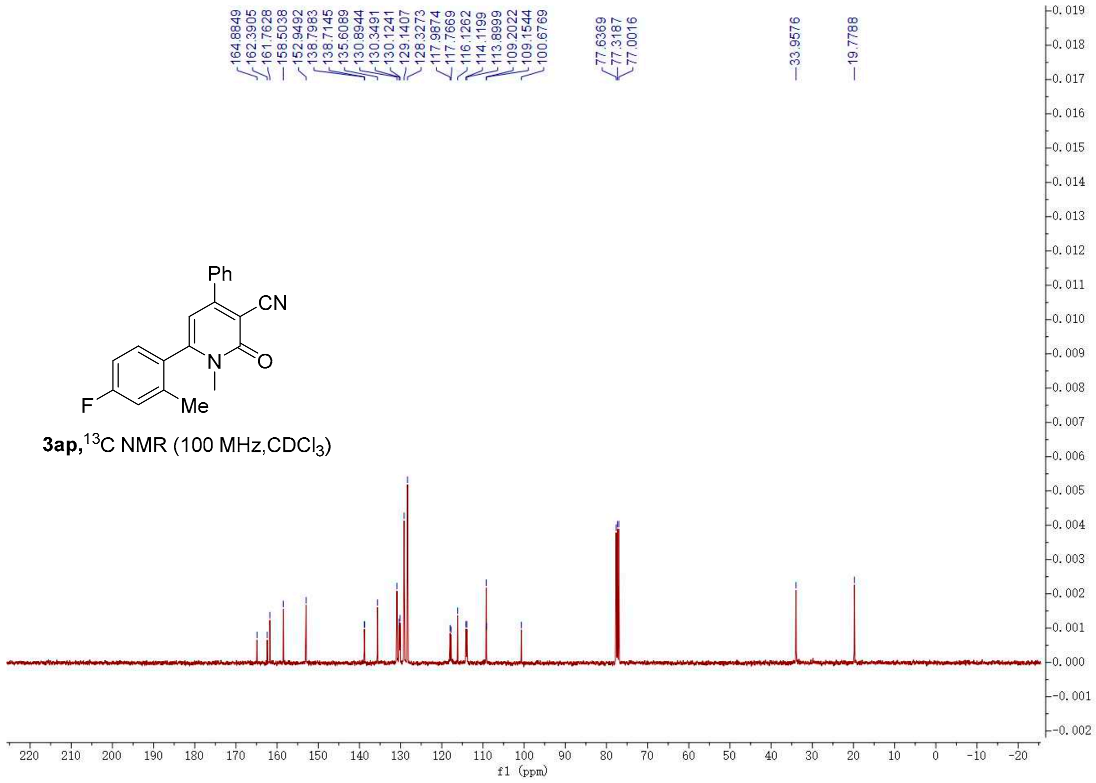

3ap, ^{13}C NMR (100 MHz, $CDCl_3$)

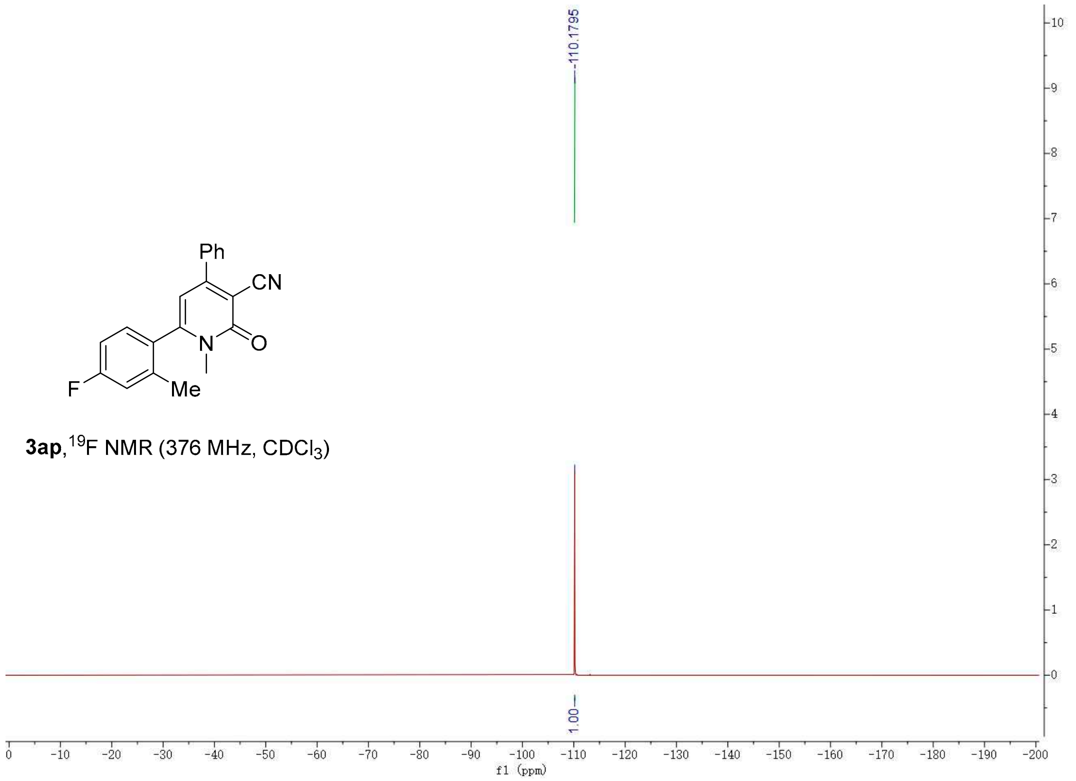

3ap, ^{19}F NMR (376 MHz, $CDCl_3$)

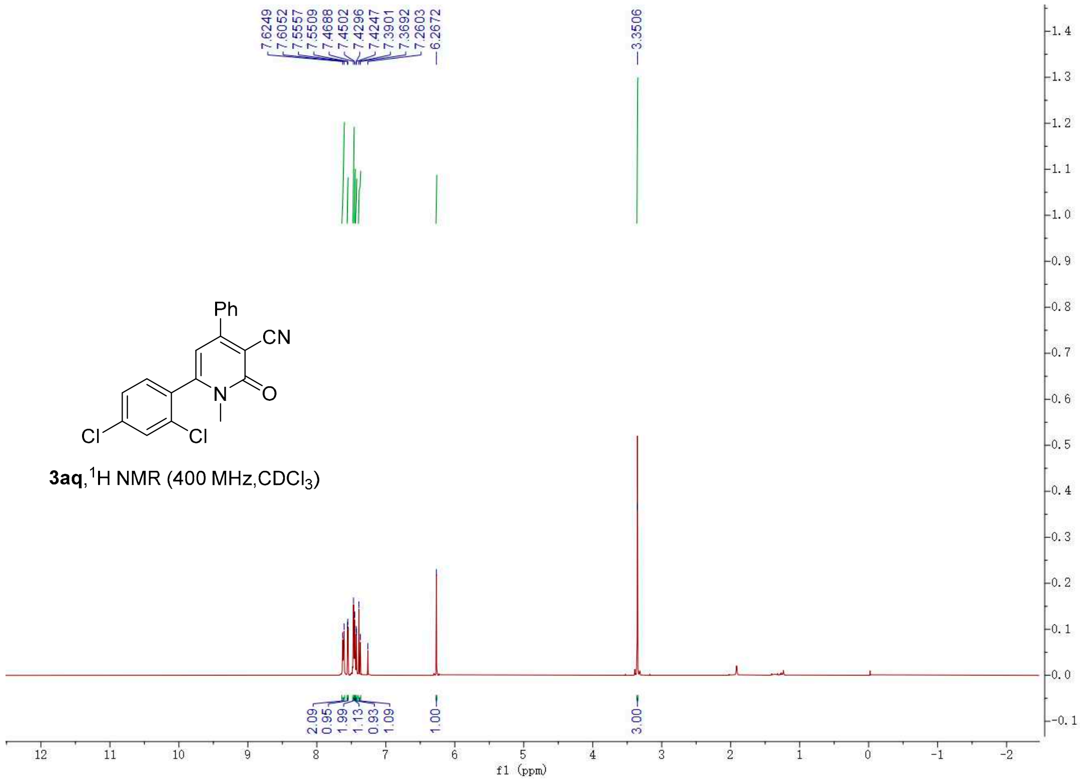

3aq, ^{1}H NMR (400 MHz, $CDCl_3$)

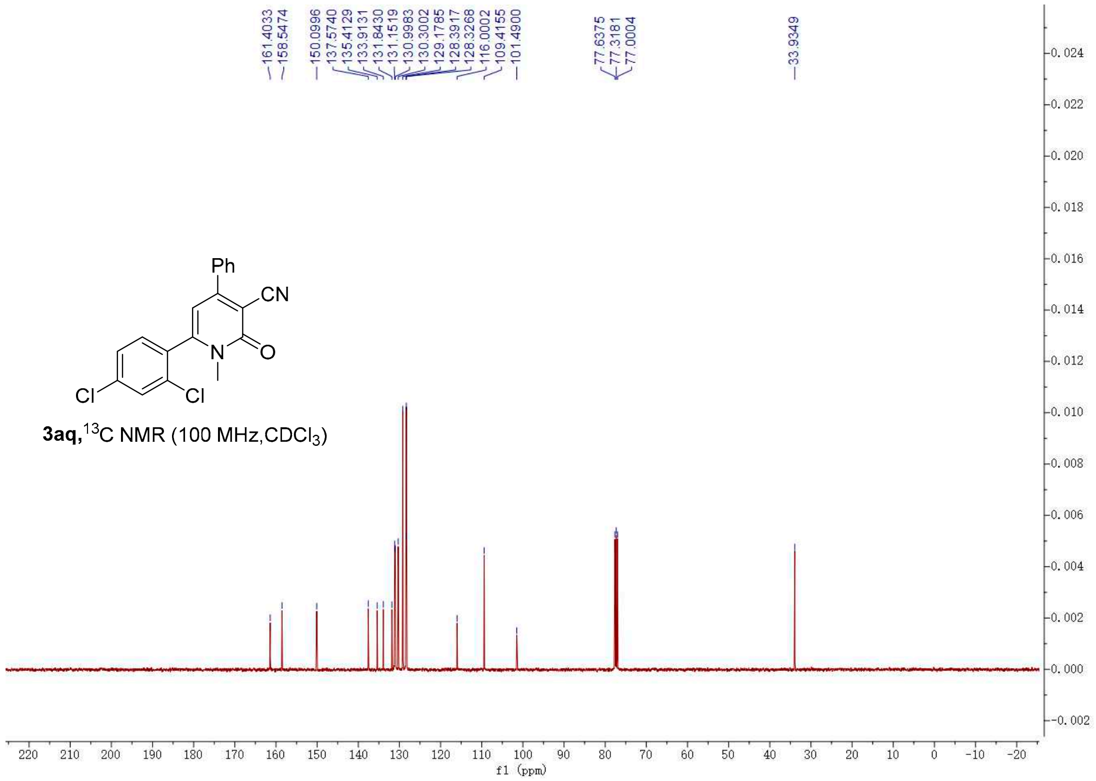

3aq,^{13}C NMR (100 MHz,CDCl$_3$)

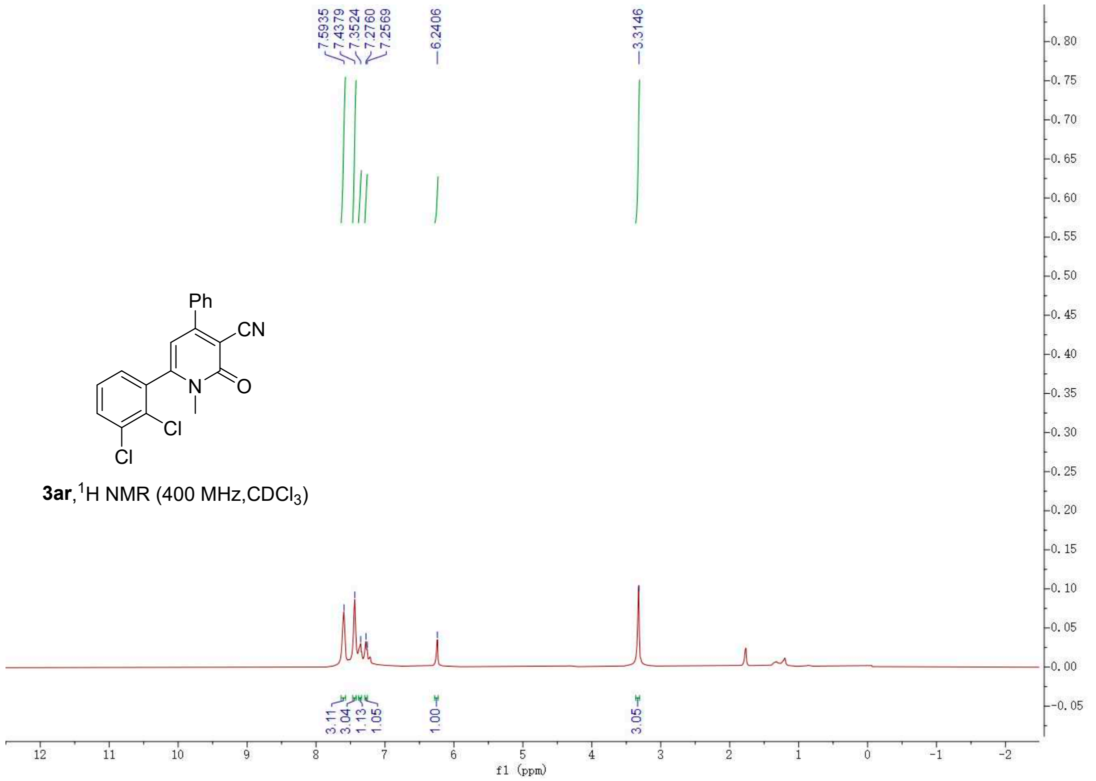

3ar,^{1}H NMR (400 MHz,CDCl$_3$)

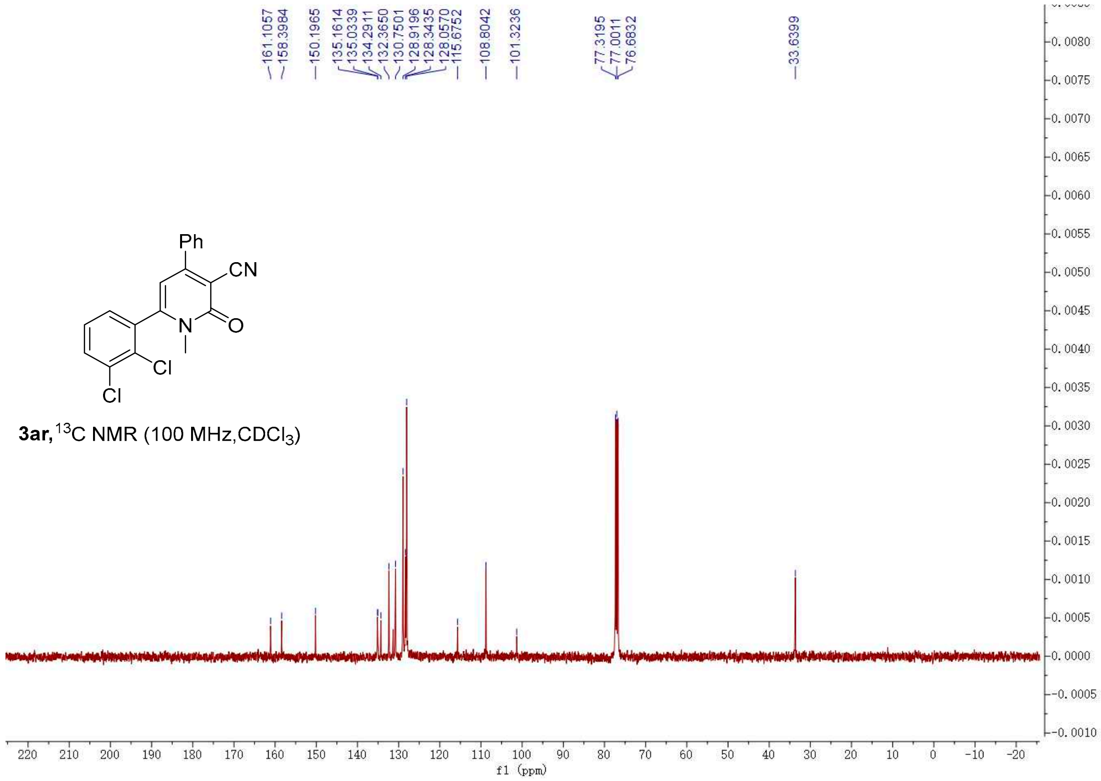

3ar, ^{13}C NMR (100 MHz,CDCl$_3$)

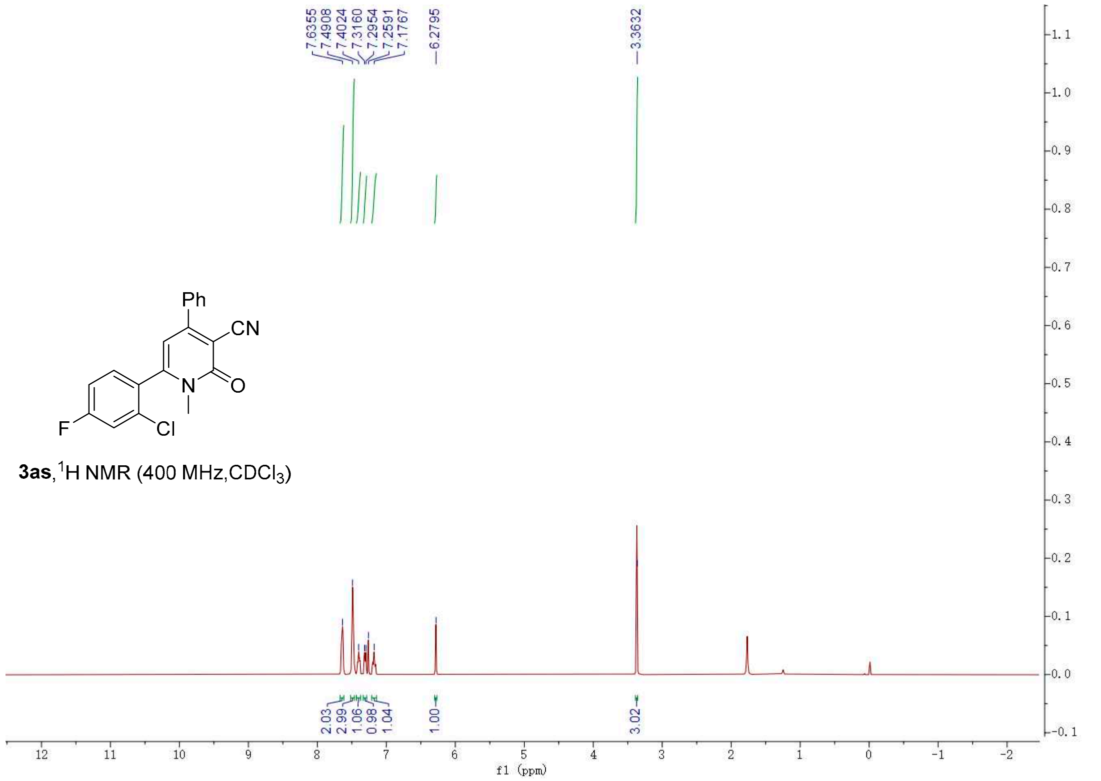

3as,^{1}H NMR (400 MHz,CDCl$_3$)

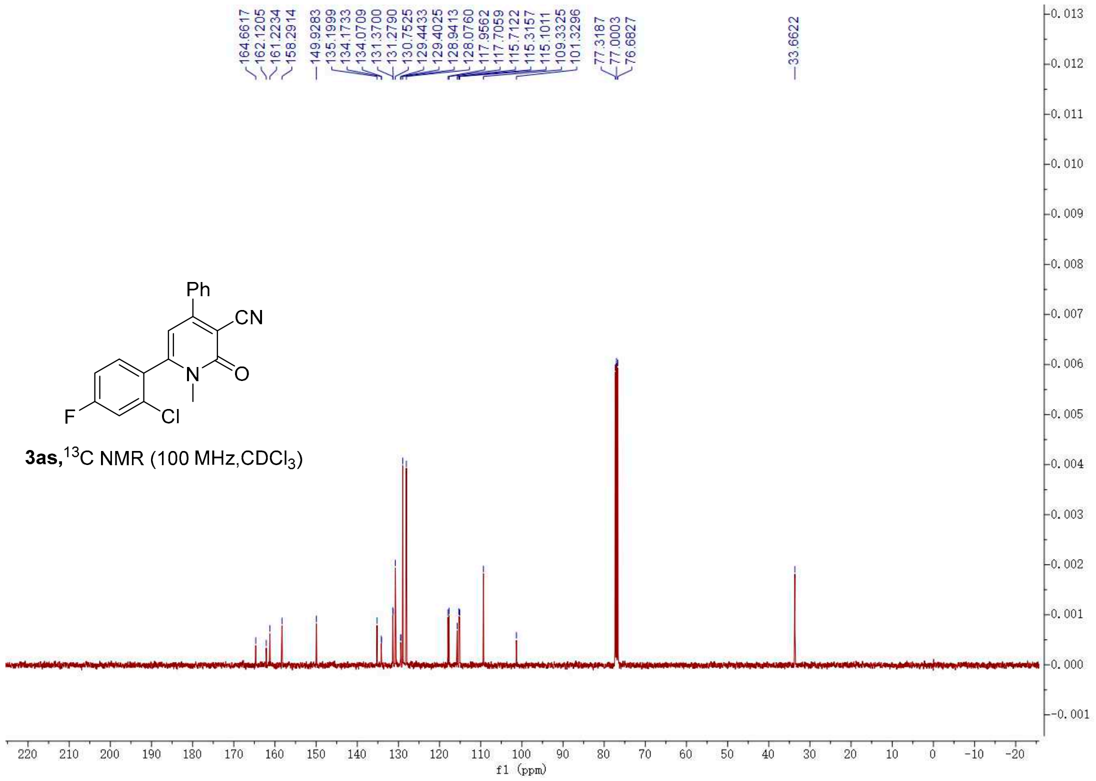

3as, ^{13}C NMR (100 MHz,$CDCl_3$)

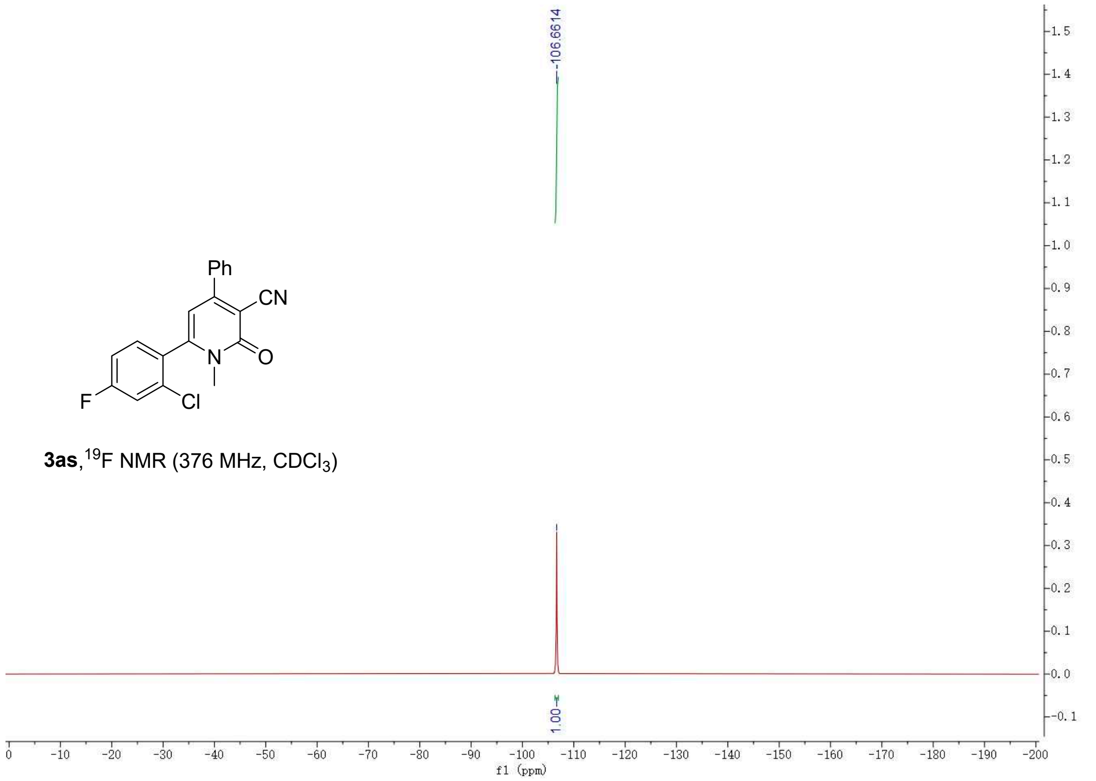

3as, ^{19}F NMR (376 MHz, $CDCl_3$)

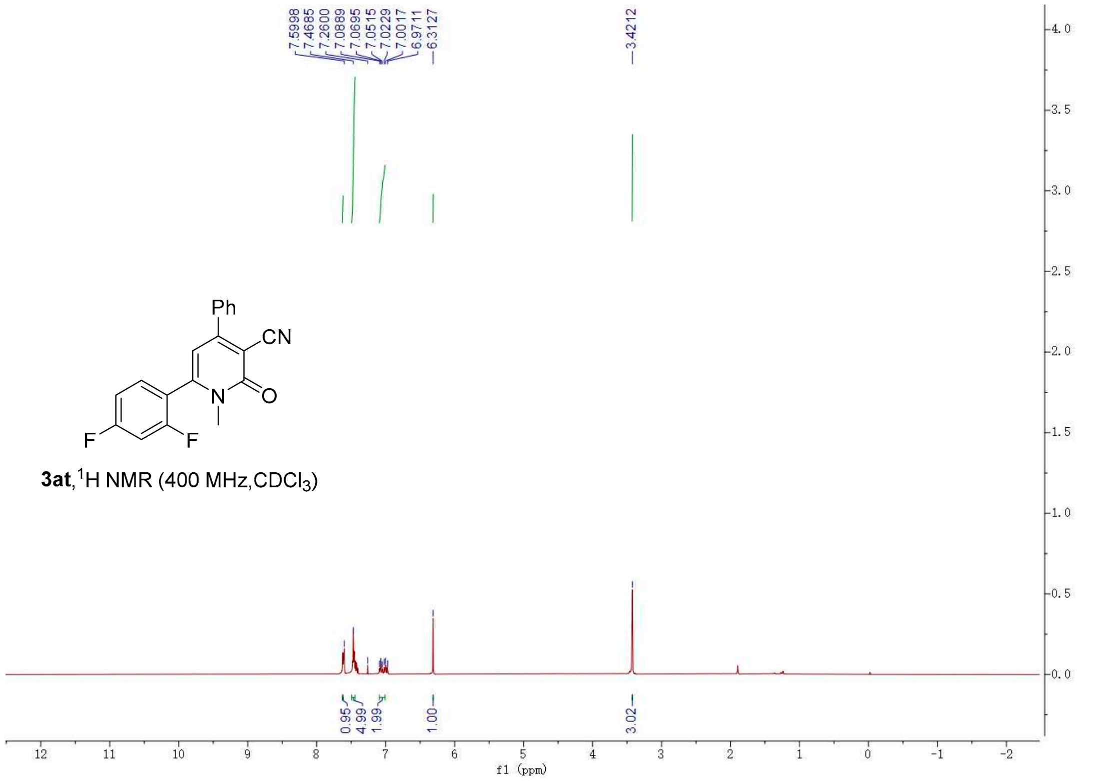

3at,^{1}H NMR (400 MHz,$CDCl_3$)

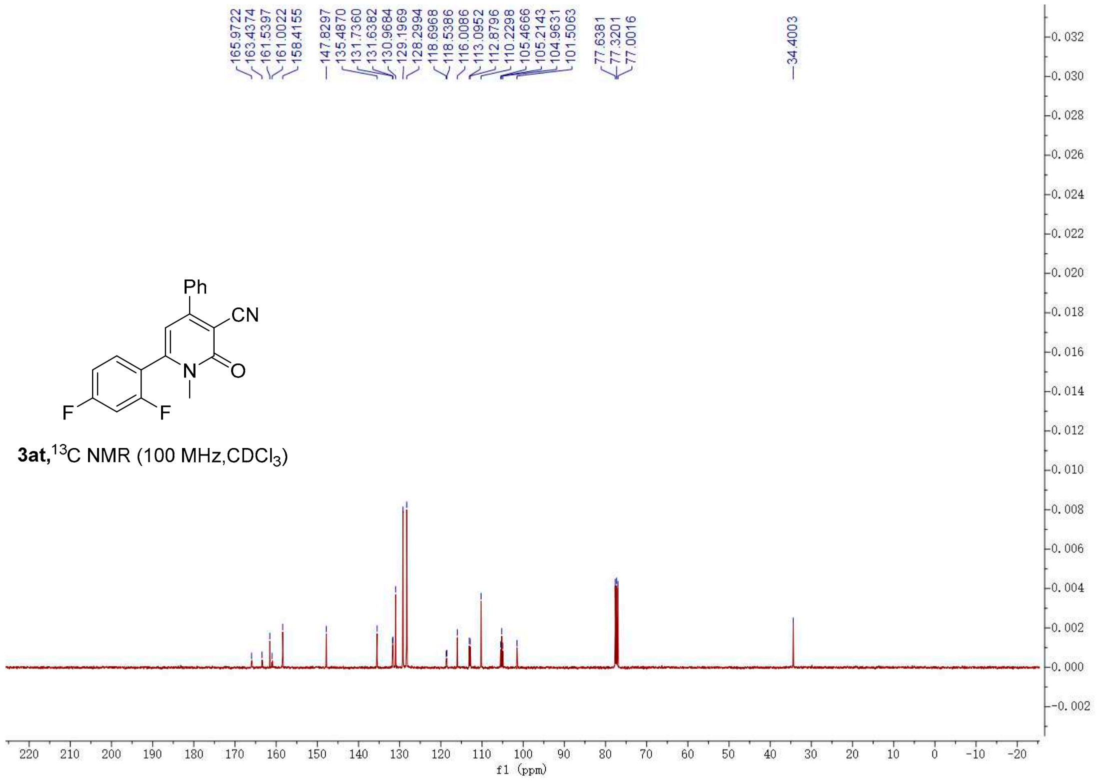

3at,^{13}C NMR (100 MHz,$CDCl_3$)

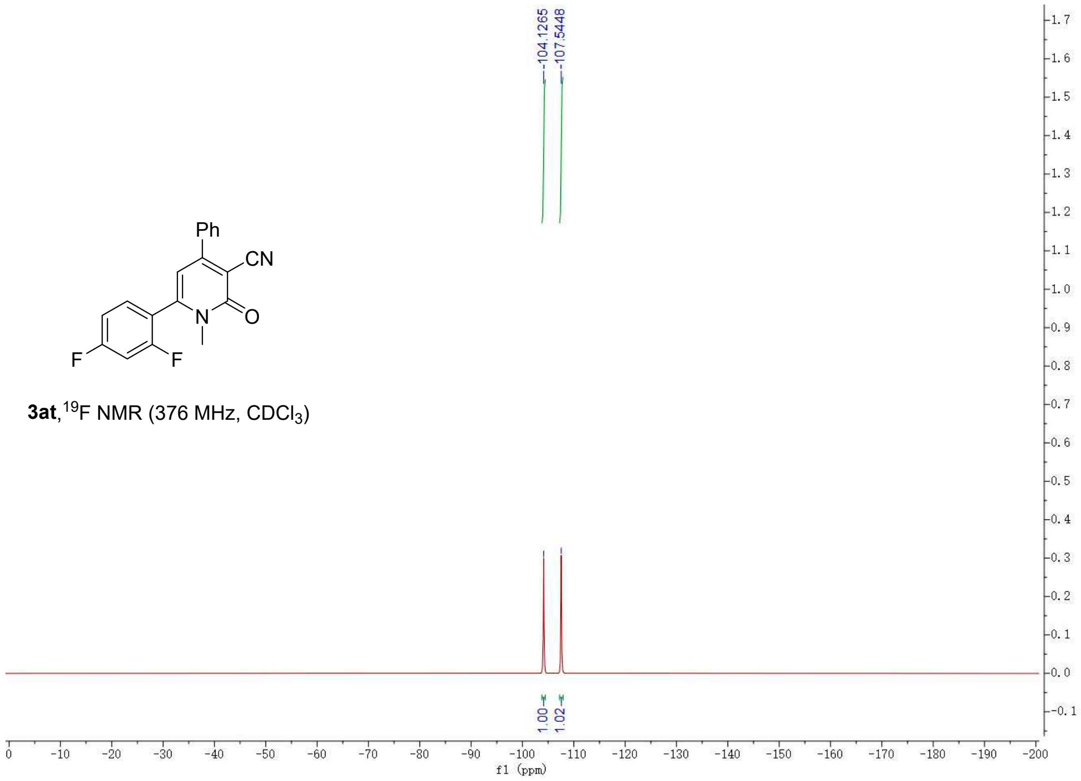

3at, ^{19}F NMR (376 MHz, $CDCl_3$)

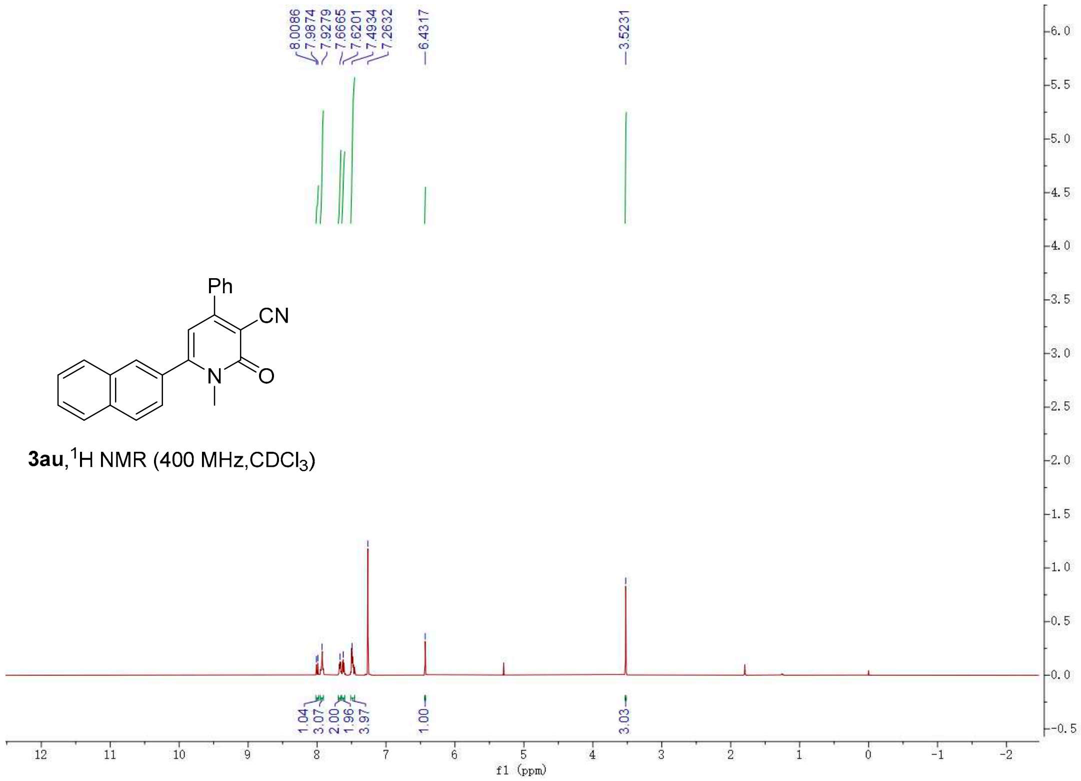

3au, ^{1}H NMR (400 MHz, $CDCl_3$)

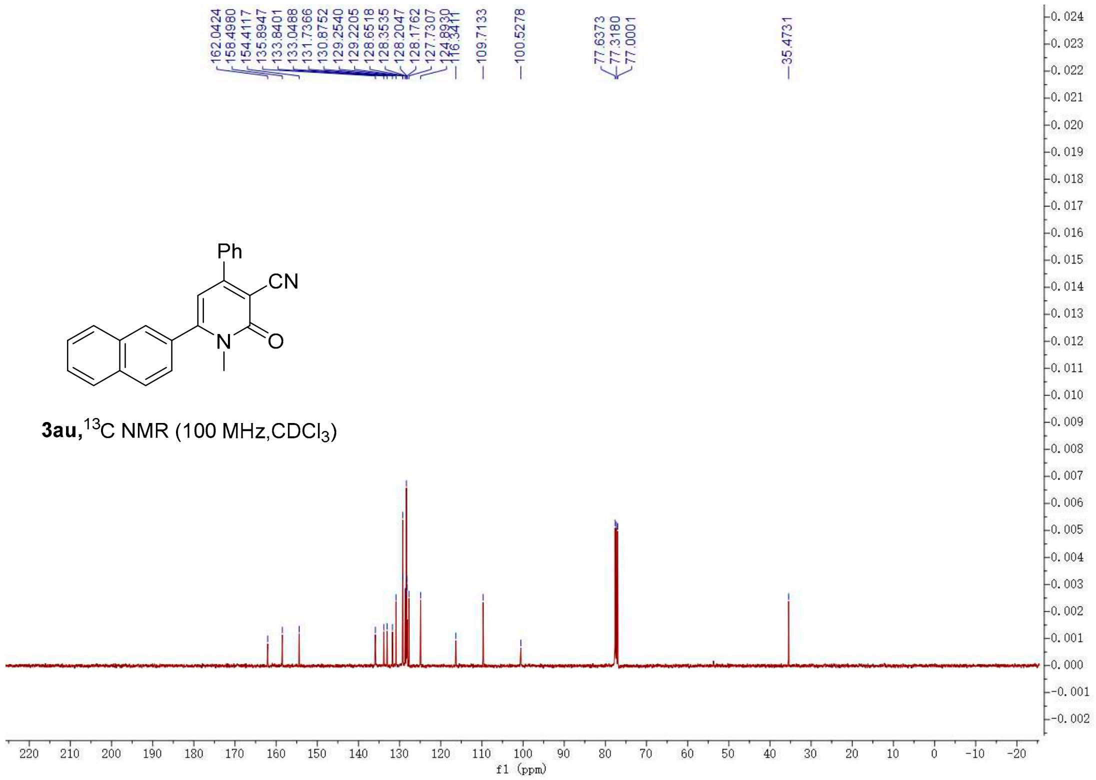
162.0424
158.4980
154.4117
135.8947
133.8401
133.0488
131.7366
130.8752
129.2540
129.2205
128.6518
128.3535
128.2047
128.1762
127.7307
124.8930
116.3411
109.7133
100.5278
77.6373
77.3180
77.0001
35.4731
Ph
CN
N
O
3au, ^{13}C NMR (100 MHz,CDCl$_3$)
f1 (ppm)

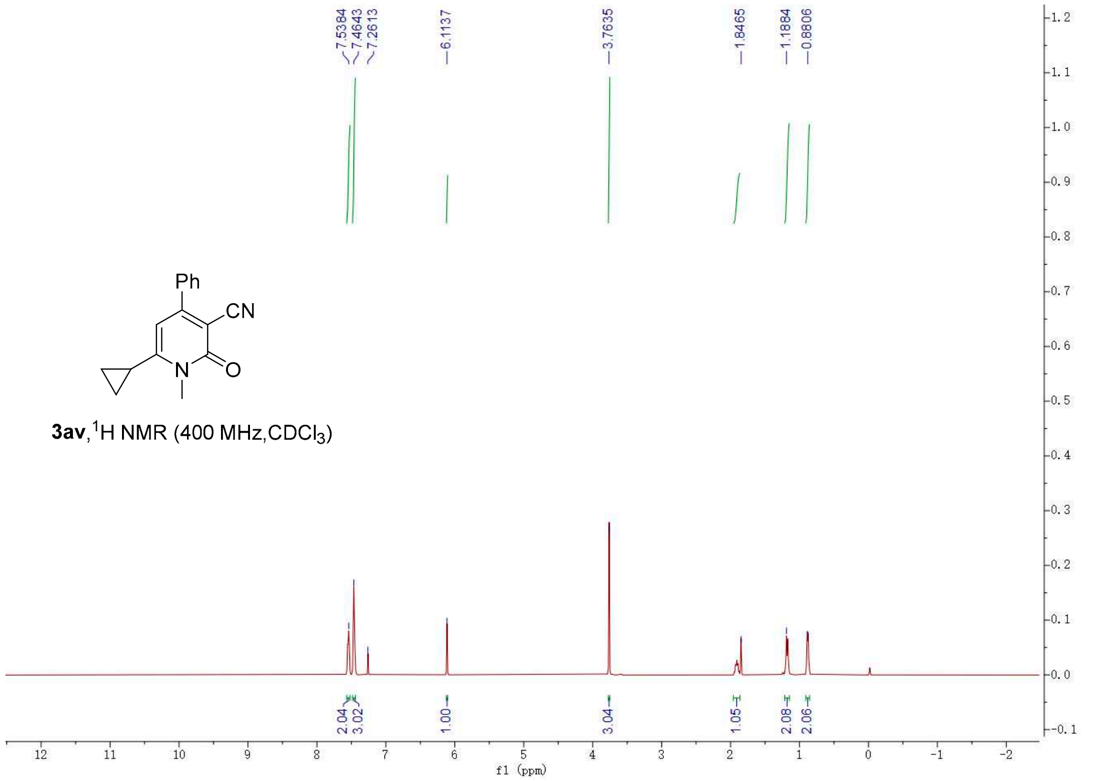
7.5384
7.4643
7.2613
6.1137
3.7635
1.8465
1.1884
0.8806
Ph
CN
N
O
3av, ^{1}H NMR (400 MHz,CDCl$_3$)
2.04
3.02
1.00
3.04
1.05
2.08
2.06
f1 (ppm)

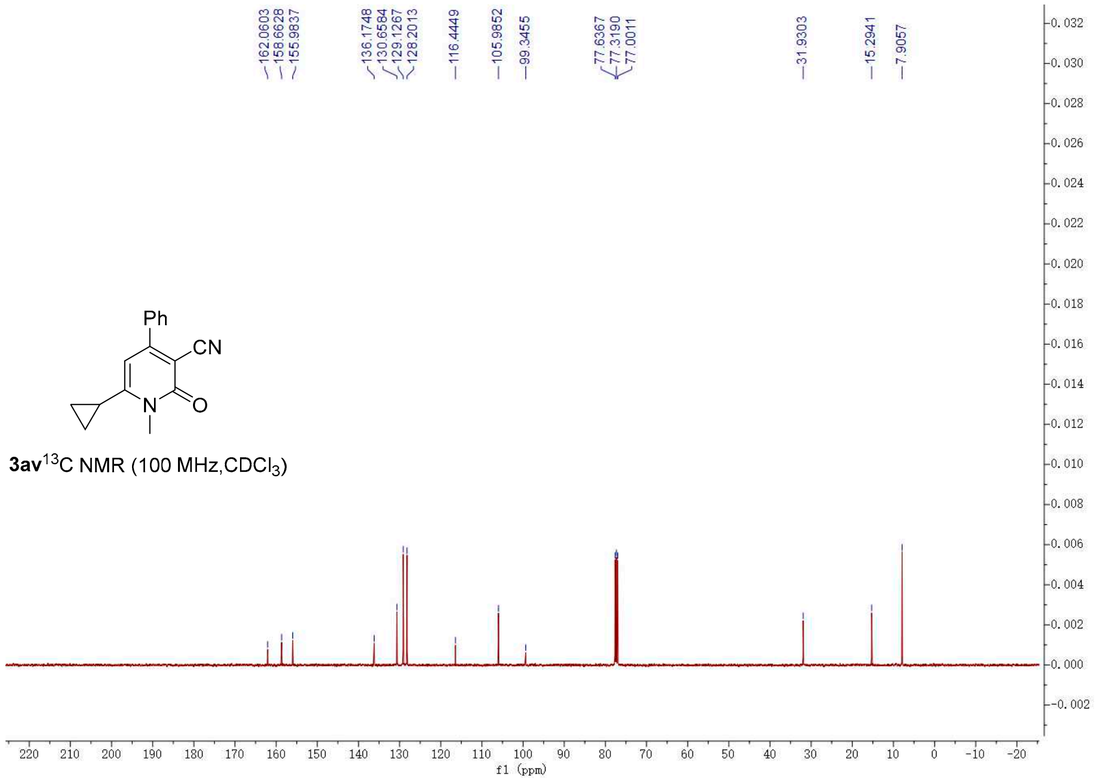

3av^{13}C NMR (100 MHz,CDCl$_3$)

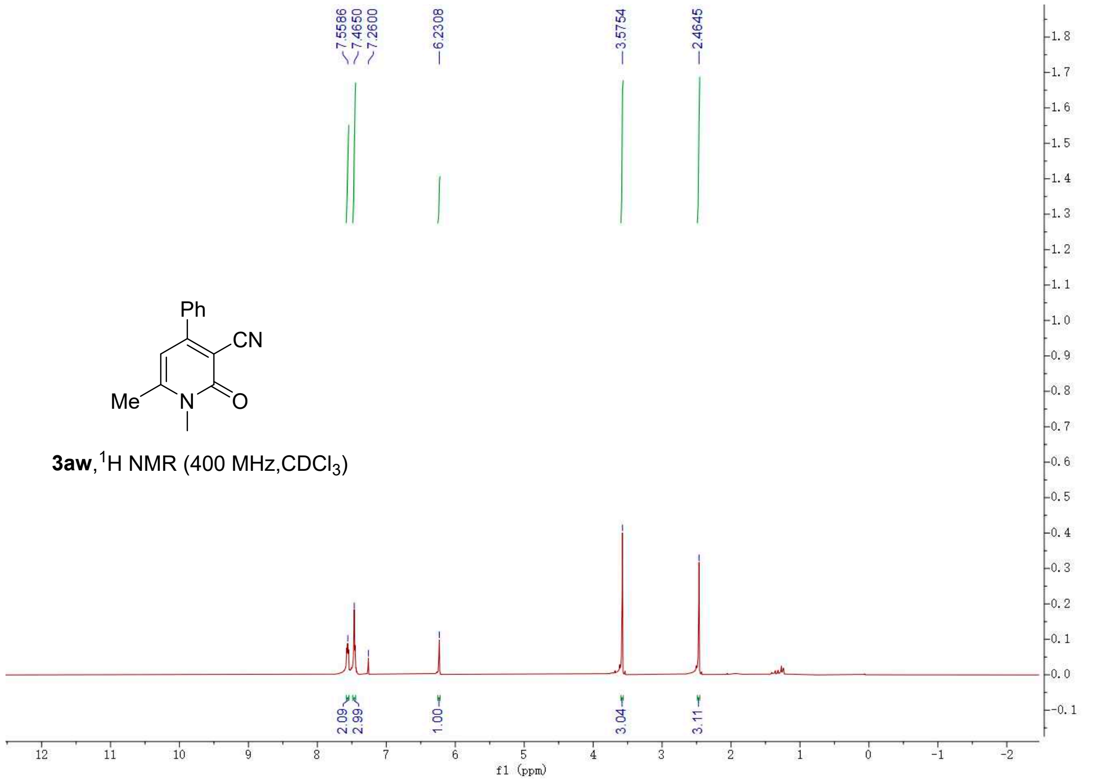

3aw,^{1}H NMR (400 MHz,CDCl$_3$)

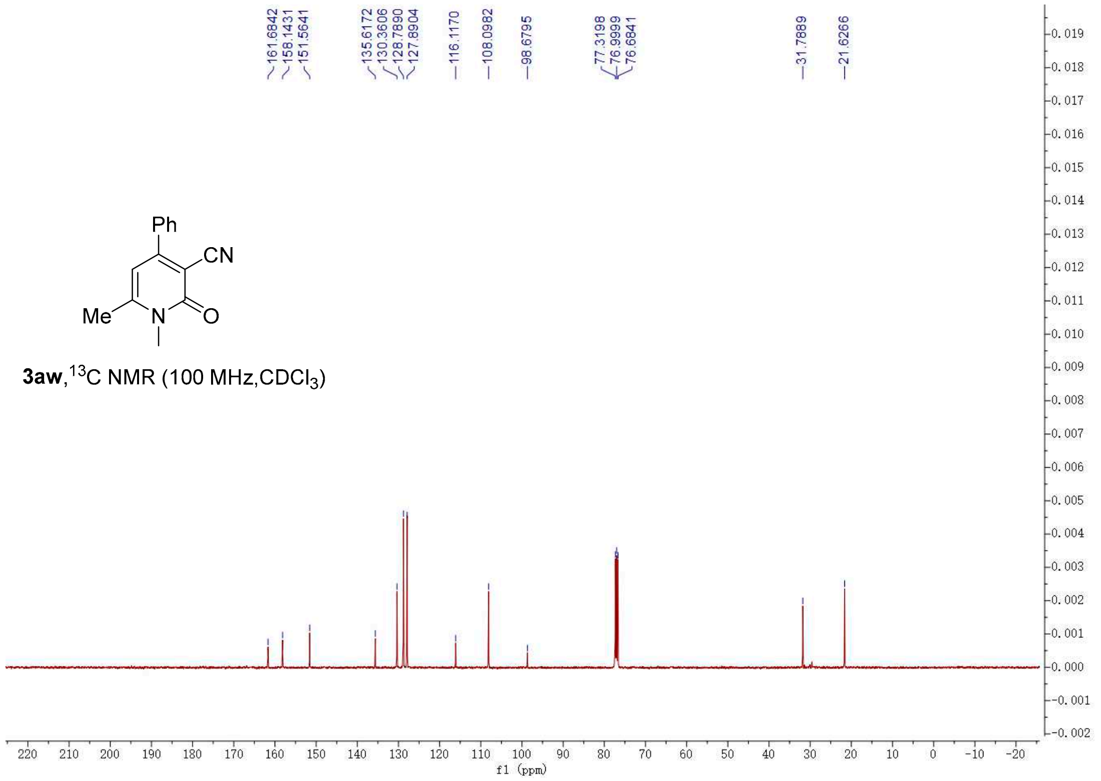

3aw, ^{13}C NMR (100 MHz, $CDCl_3$)

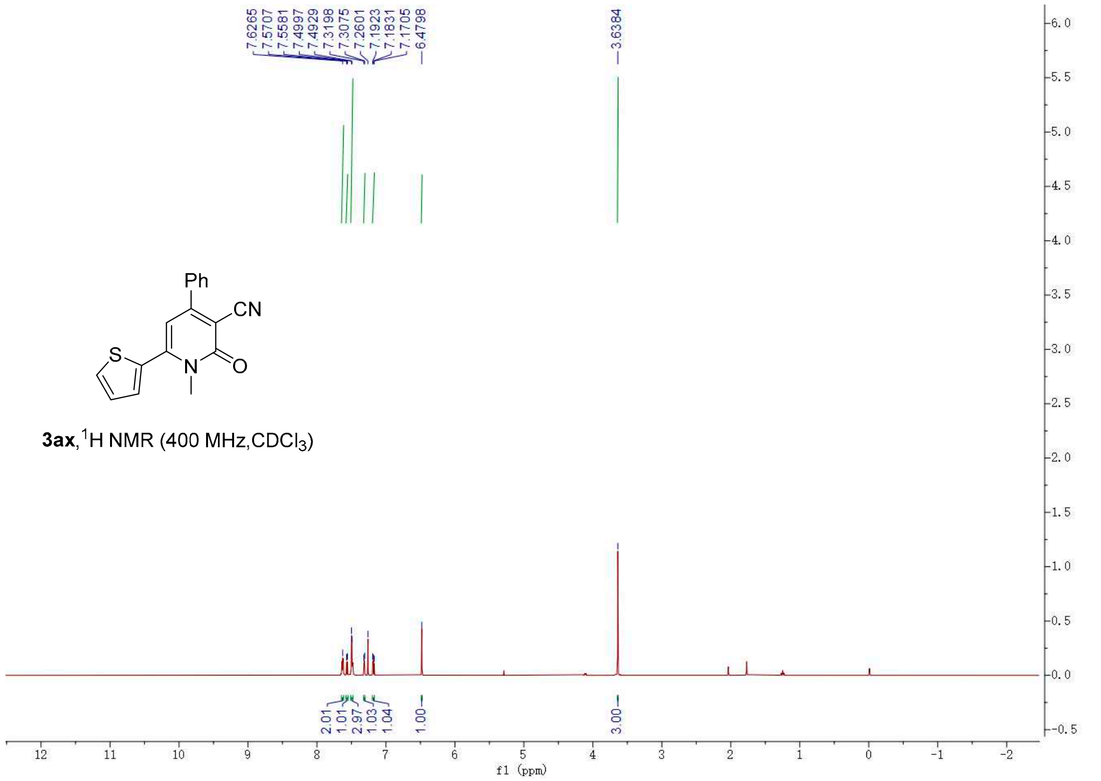

3ax, ^{1}H NMR (400 MHz, $CDCl_3$)

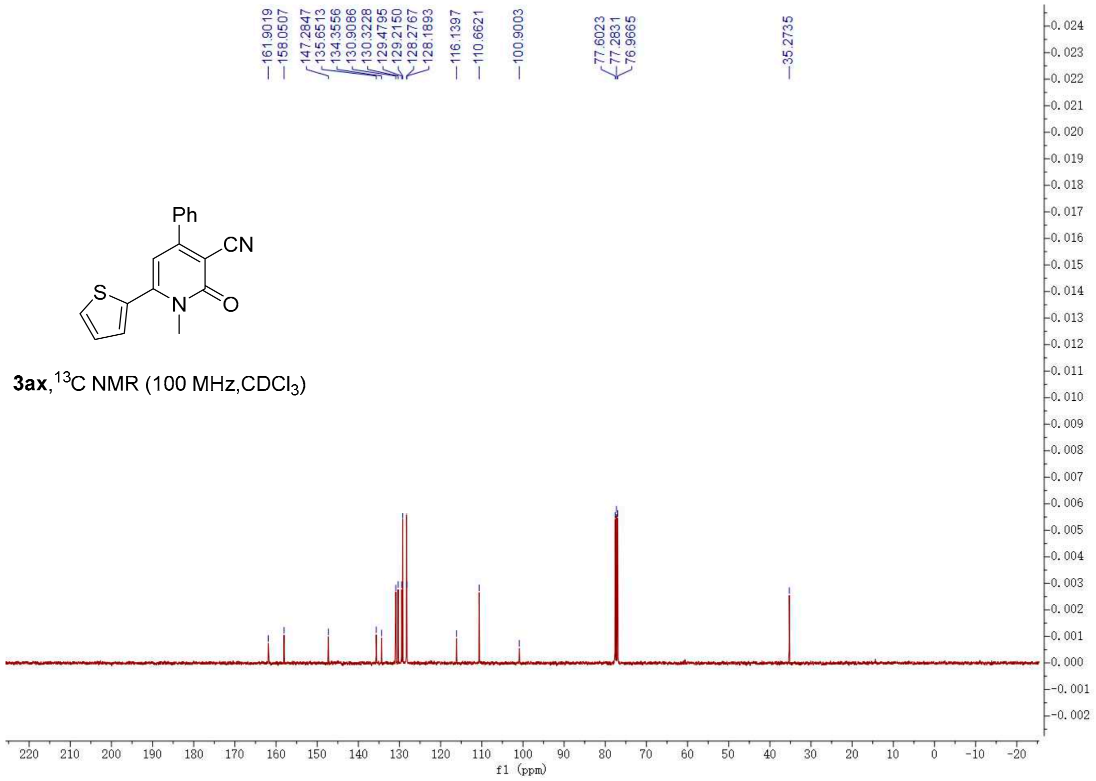

3ax, ^{13}C NMR (100 MHz, $CDCl_3$)

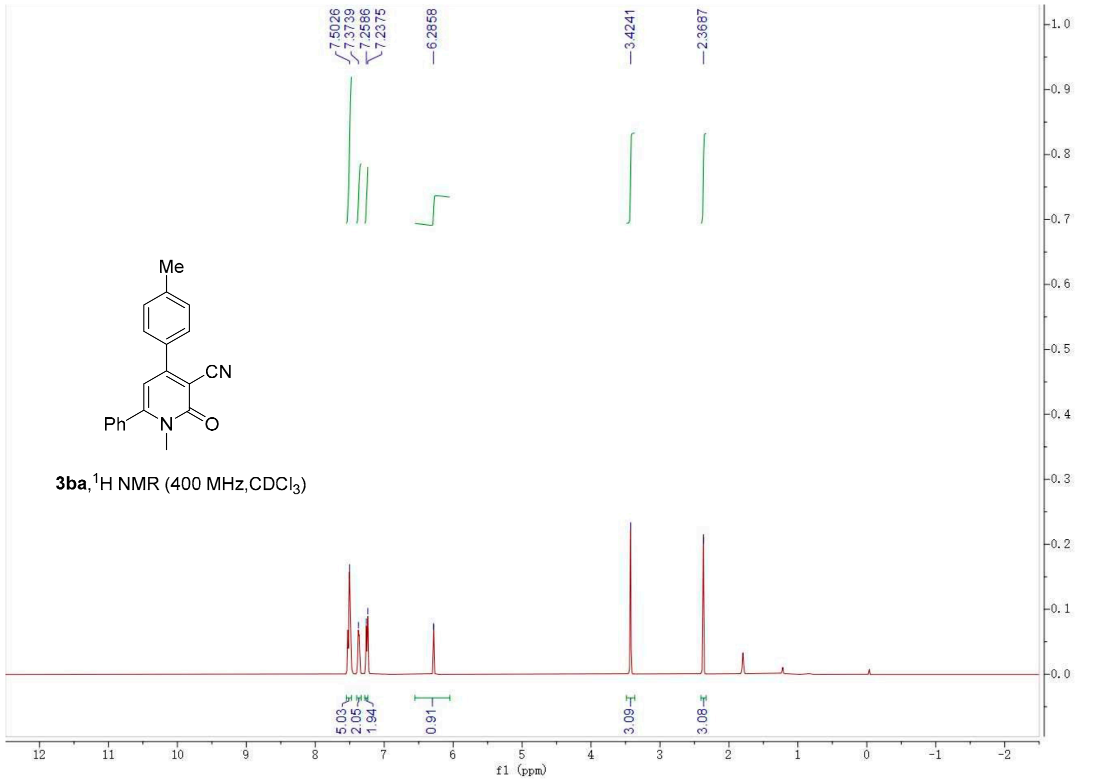

3ba, ^{1}H NMR (400 MHz, $CDCl_3$)

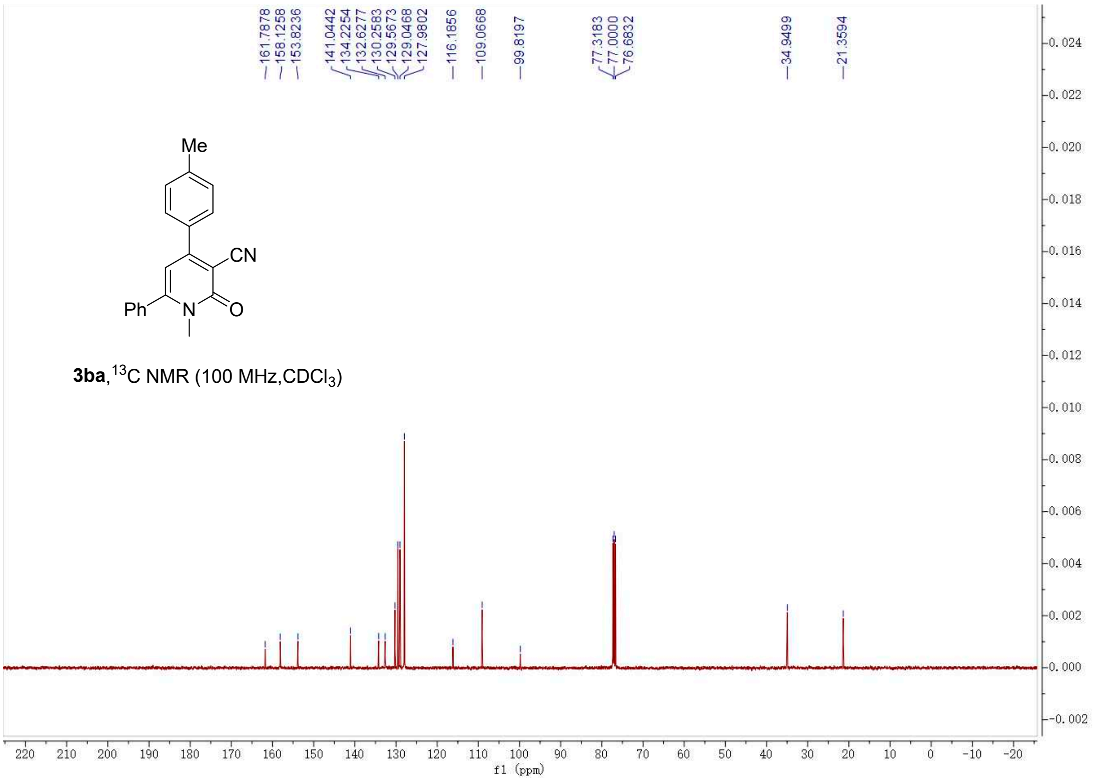

3ba, ^{13}C NMR (100 MHz, $CDCl_3$)

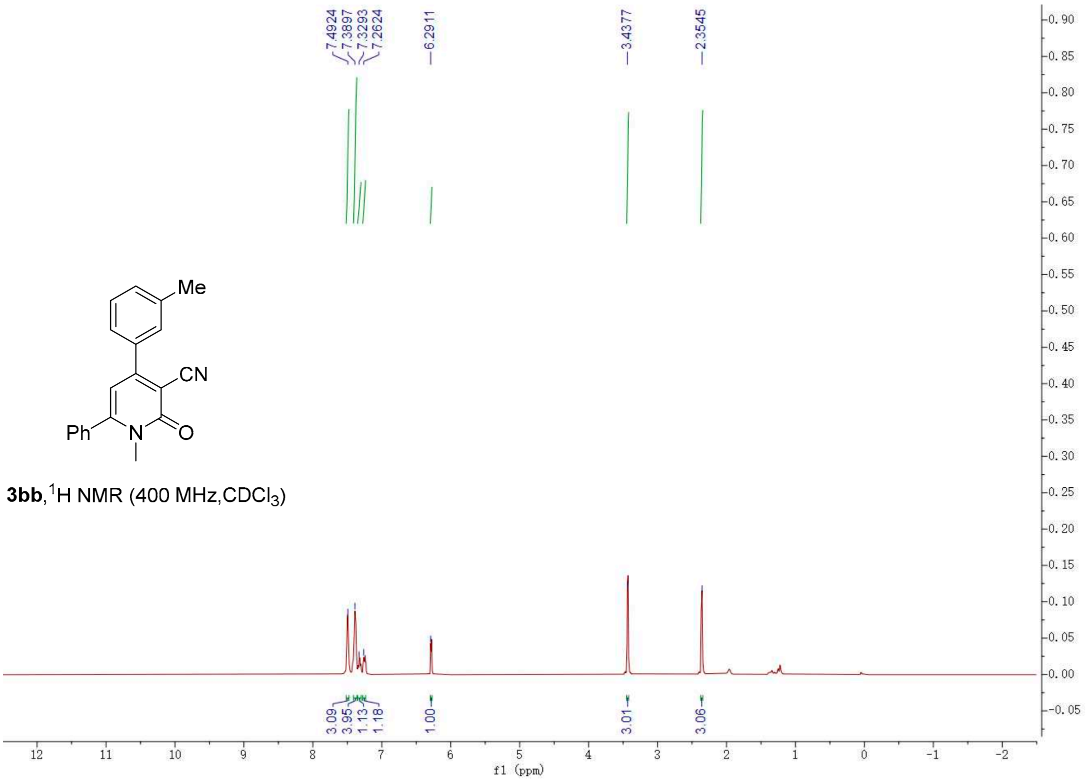

3bb, ^{1}H NMR (400 MHz, $CDCl_3$)

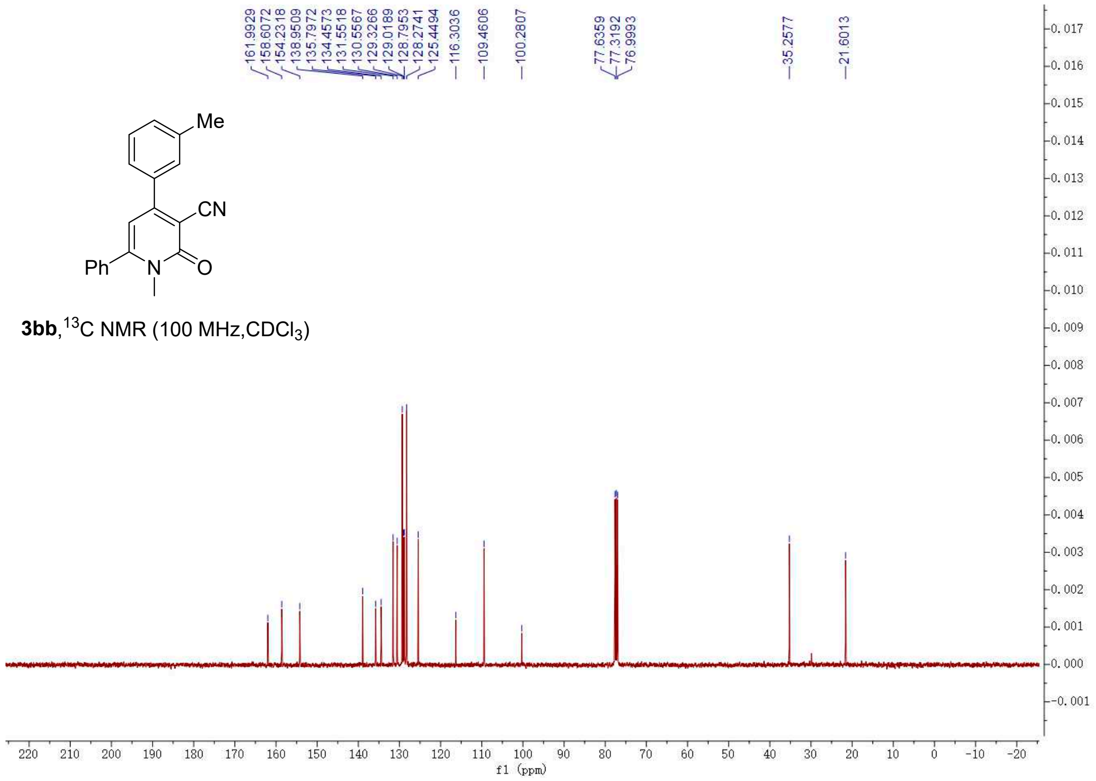

3bb, ^{13}C NMR (100 MHz, $CDCl_3$)

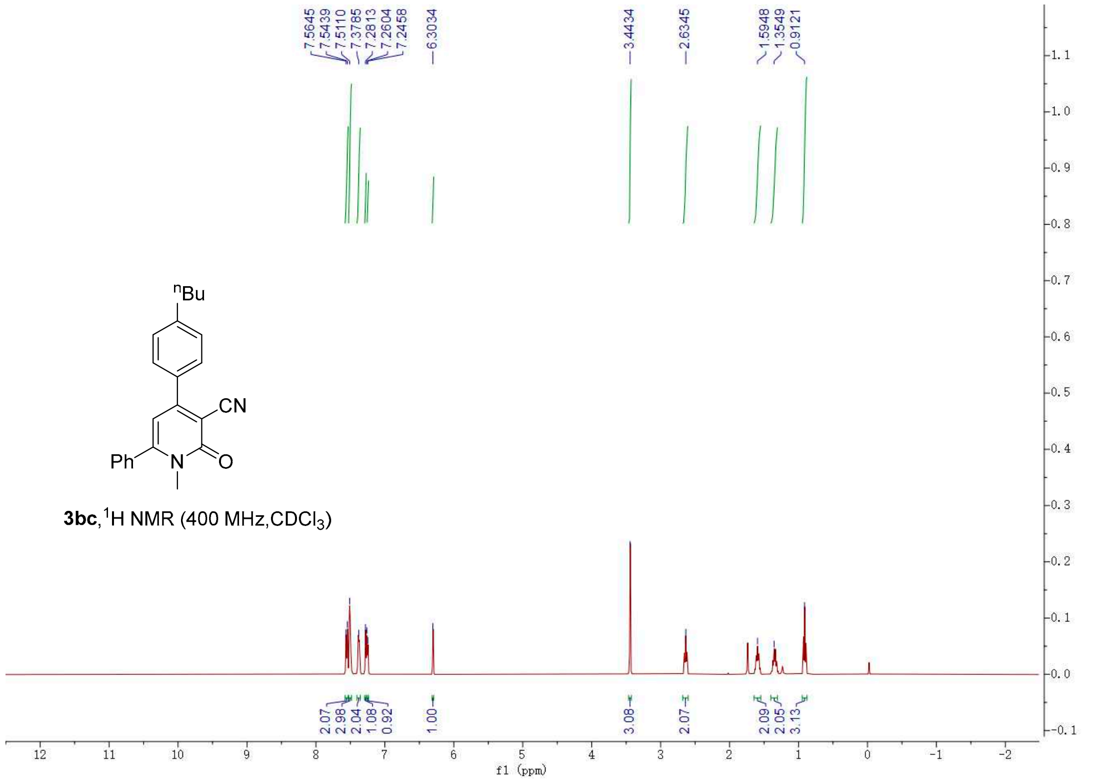

3bc, ^{1}H NMR (400 MHz, $CDCl_3$)

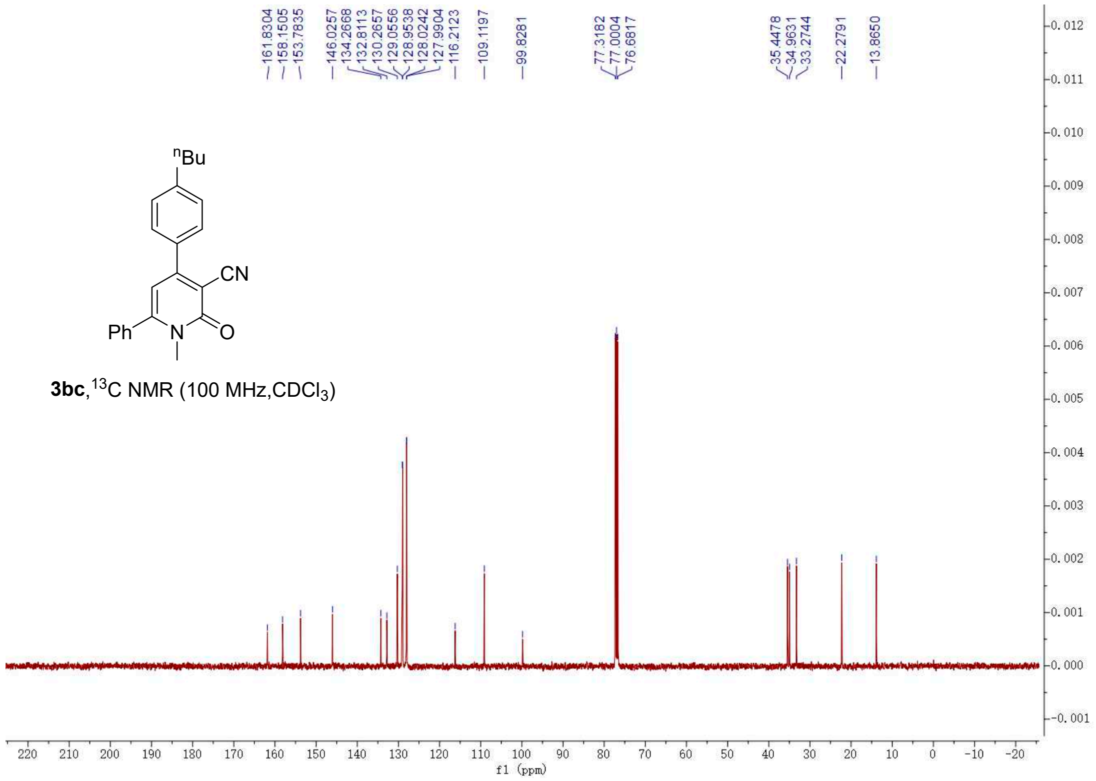

3bc, ^{13}C NMR (100 MHz, $CDCl_3$)

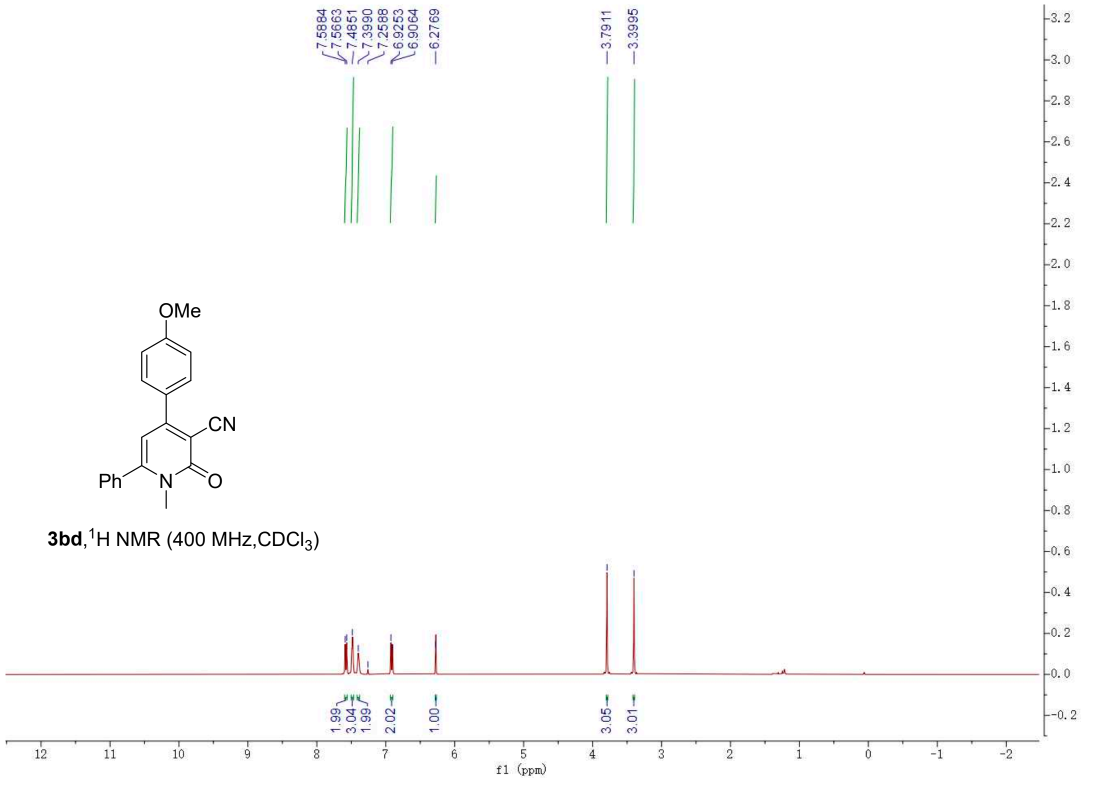

3bd, ^{1}H NMR (400 MHz, $CDCl_3$)

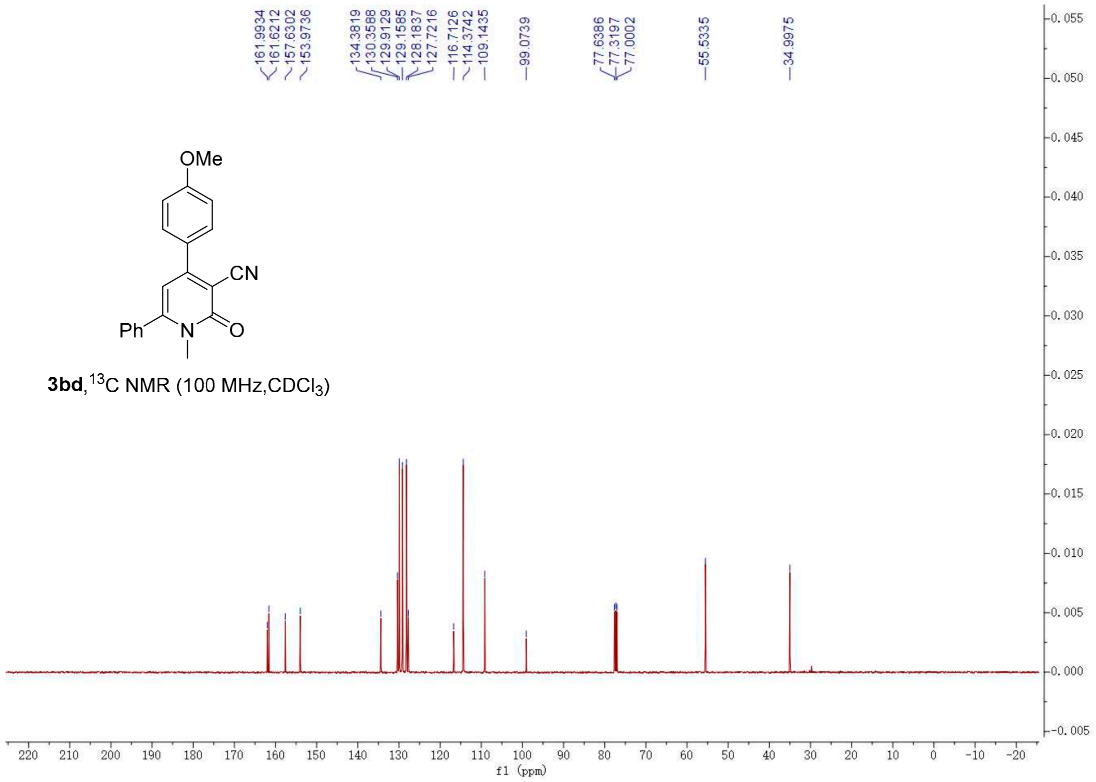

3bd, ^{13}C NMR (100 MHz, $CDCl_3$)

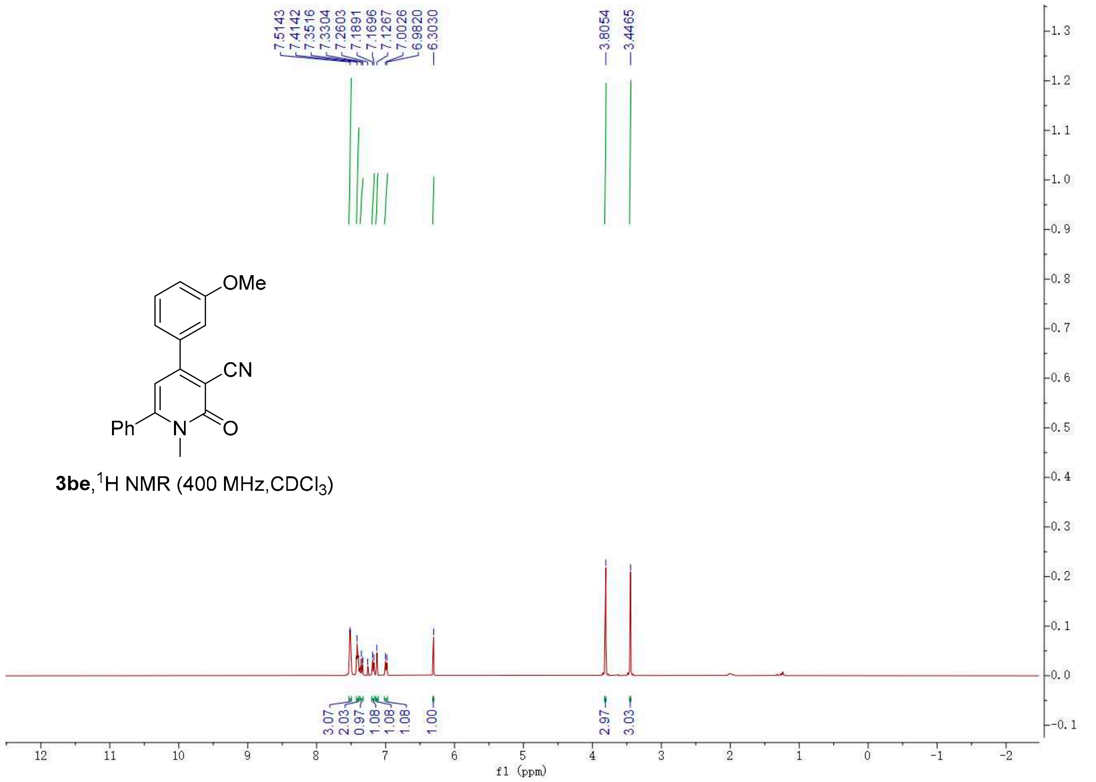

3be, ^{1}H NMR (400 MHz, $CDCl_3$)

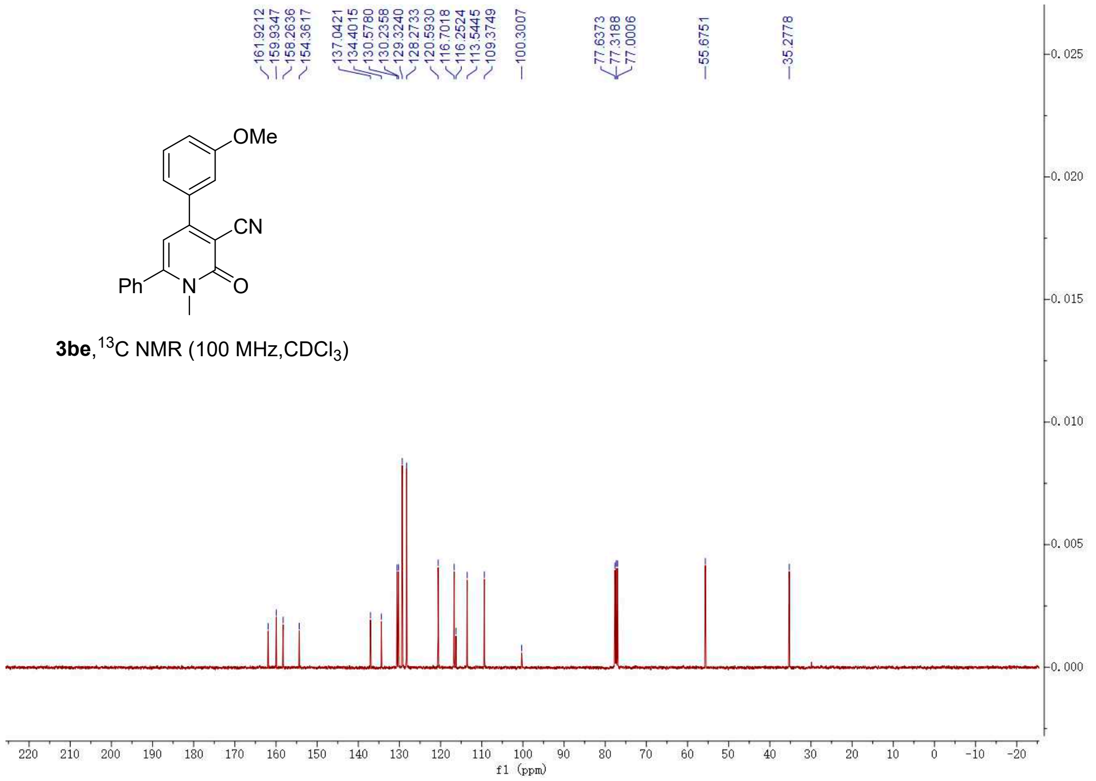

3be, ^{13}C NMR (100 MHz, $CDCl_3$)

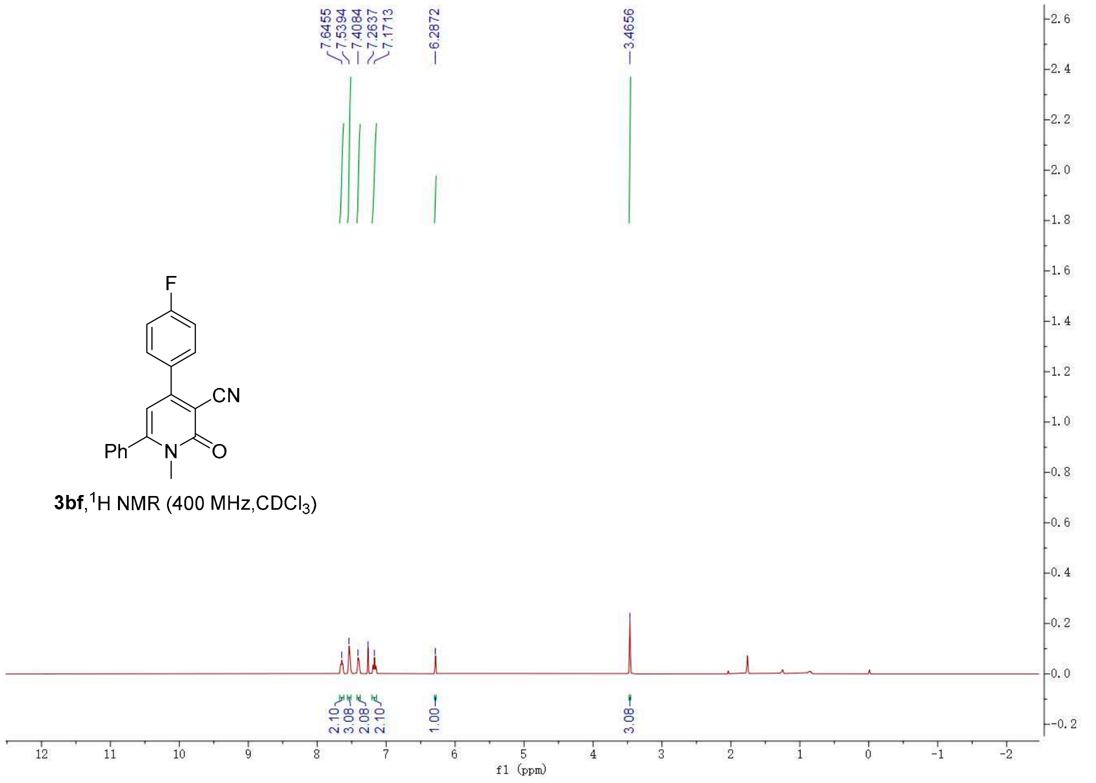

3bf, ^{1}H NMR (400 MHz, $CDCl_3$)

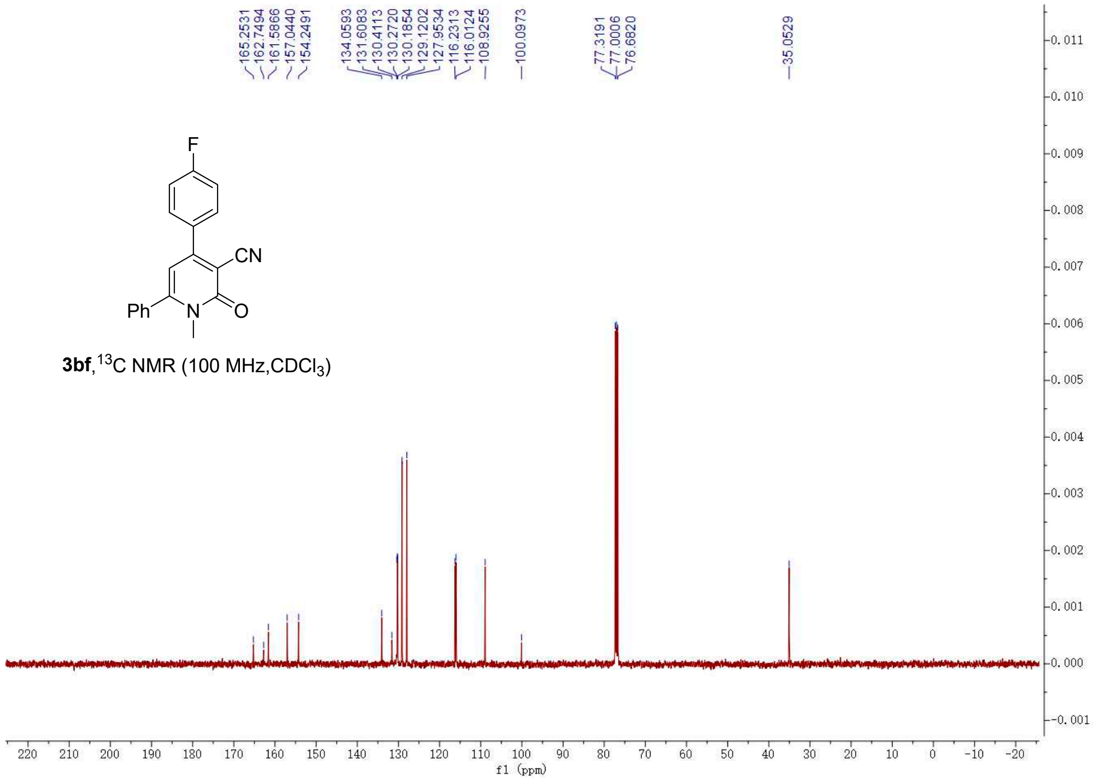

3bf, ^{13}C NMR (100 MHz, $CDCl_3$)

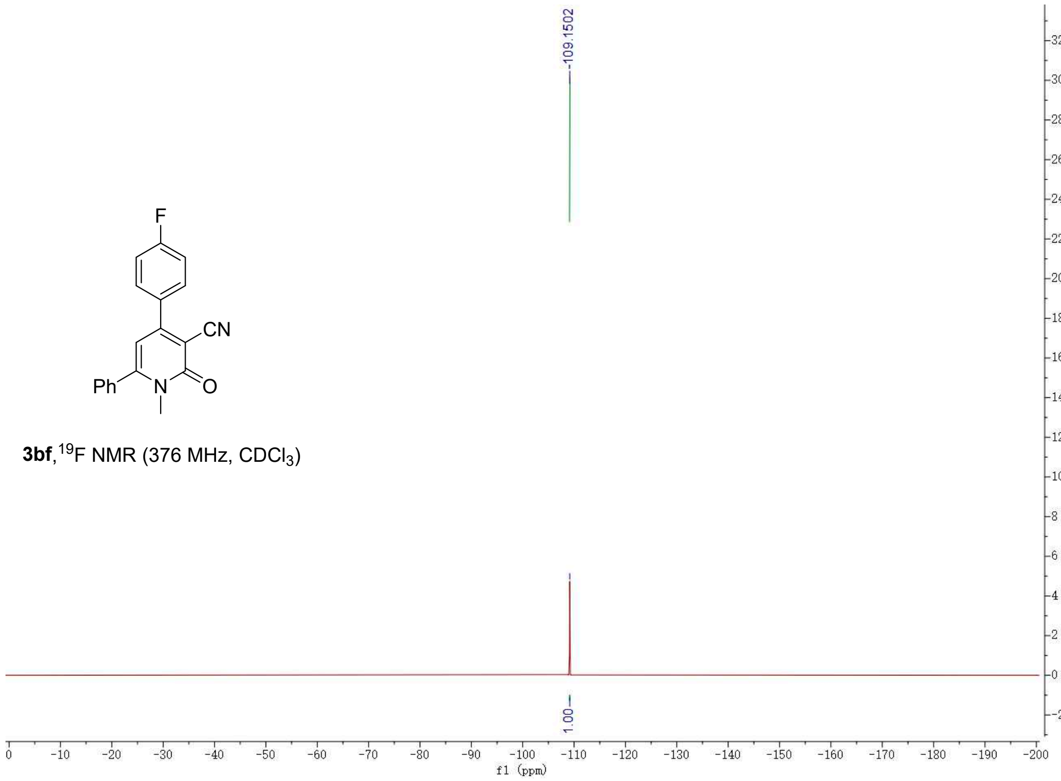

3bf, ^{19}F NMR (376 MHz, $CDCl_3$)

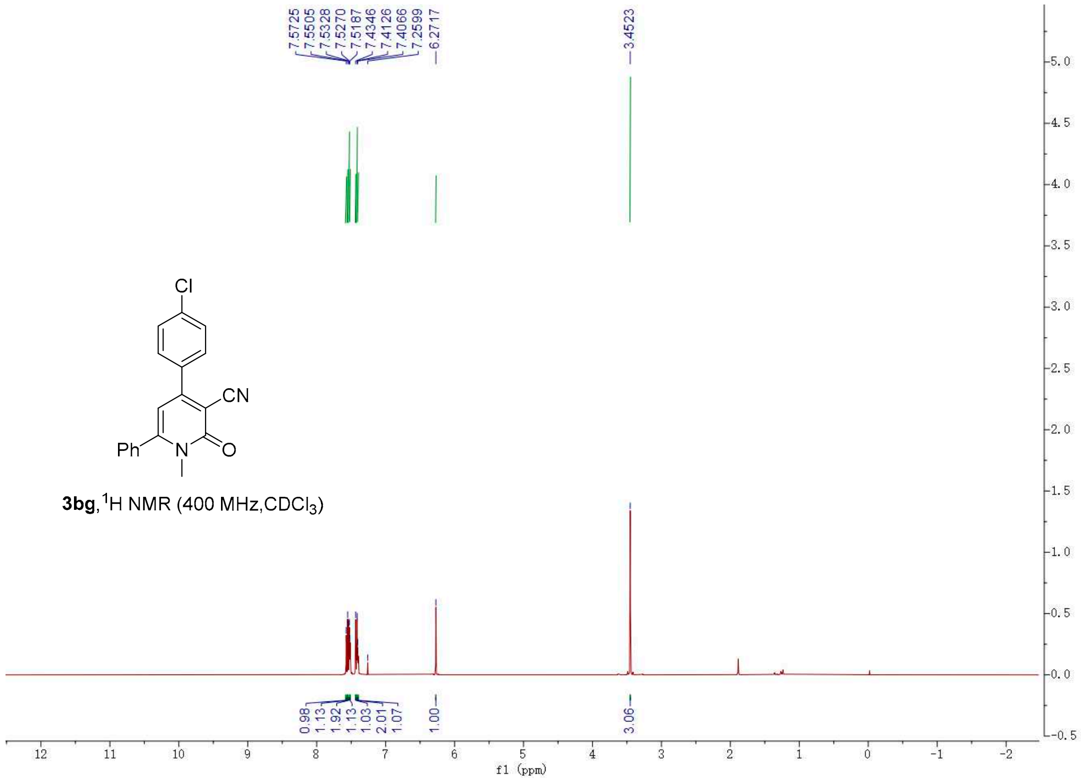
7.5725
7.5505
7.5328
7.5270
7.5187
7.4346
7.4126
7.4066
7.2599
6.2717
3.4523
0.98
1.13
1.92
1.13
1.03
2.01
1.07
1.00
3.06
Cl
CN
Ph
N
O
3bg, 1H NMR (400 MHz,CDCl3)
f1 (ppm)

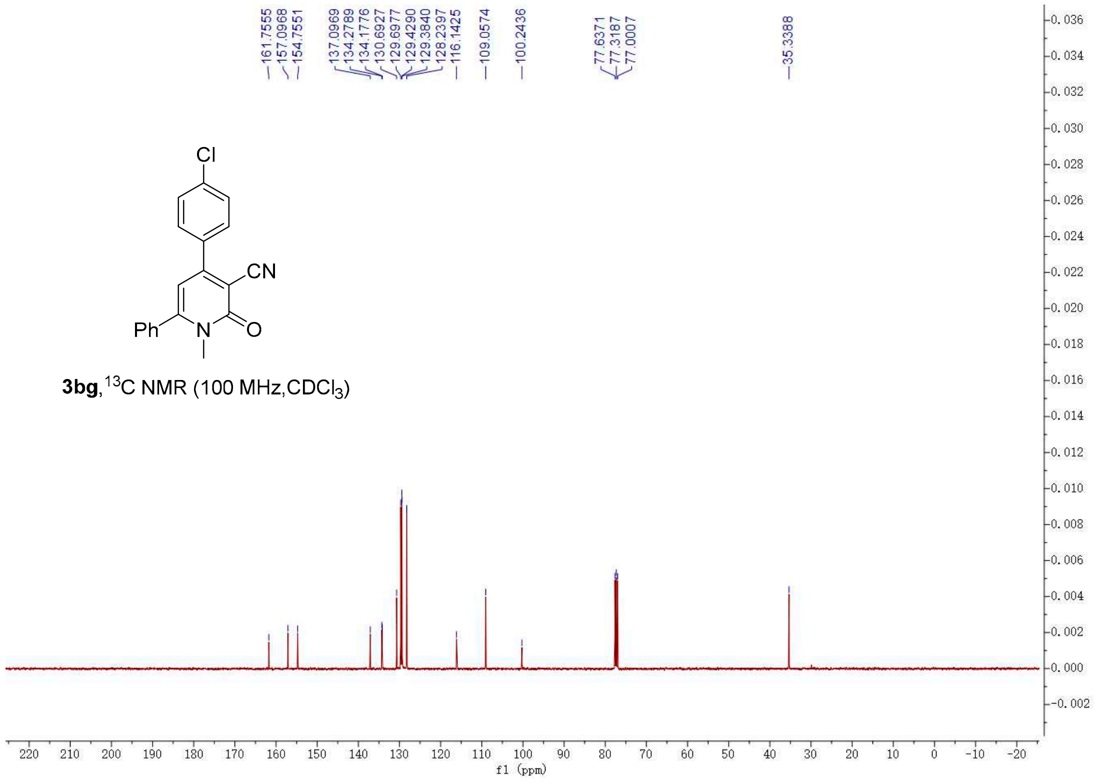
161.7555
157.0968
154.7551
137.0969
134.2789
134.1776
130.6927
129.6977
129.4290
129.3840
128.2397
116.1425
109.0574
100.2436
77.6371
77.3187
77.0007
35.3388
Cl
CN
Ph
N
O
3bg, 13C NMR (100 MHz,CDCl3)
f1 (ppm)

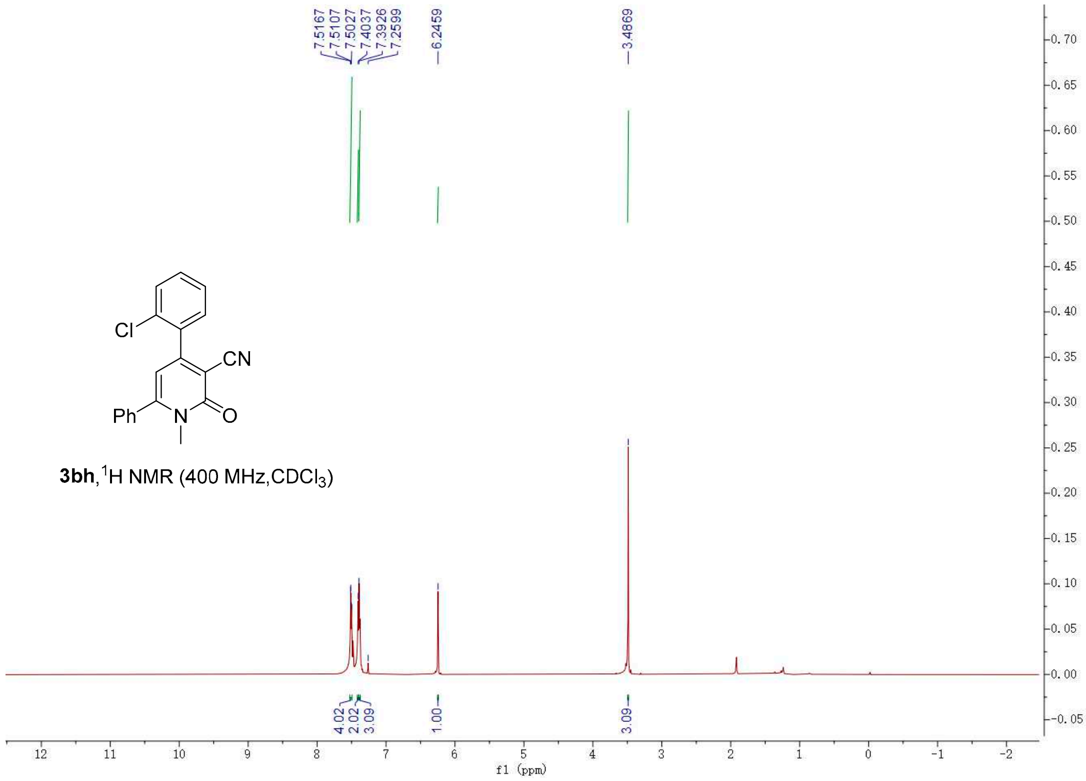
7.5167
7.5107
7.5027
7.4037
7.3926
7.2599
6.2459
3.4869
Cl
CN
Ph
N
O
3bh,^{1}H NMR (400 MHz,$CDCl_3$)
4.02
2.02
3.09
1.00
3.09
f1 (ppm)

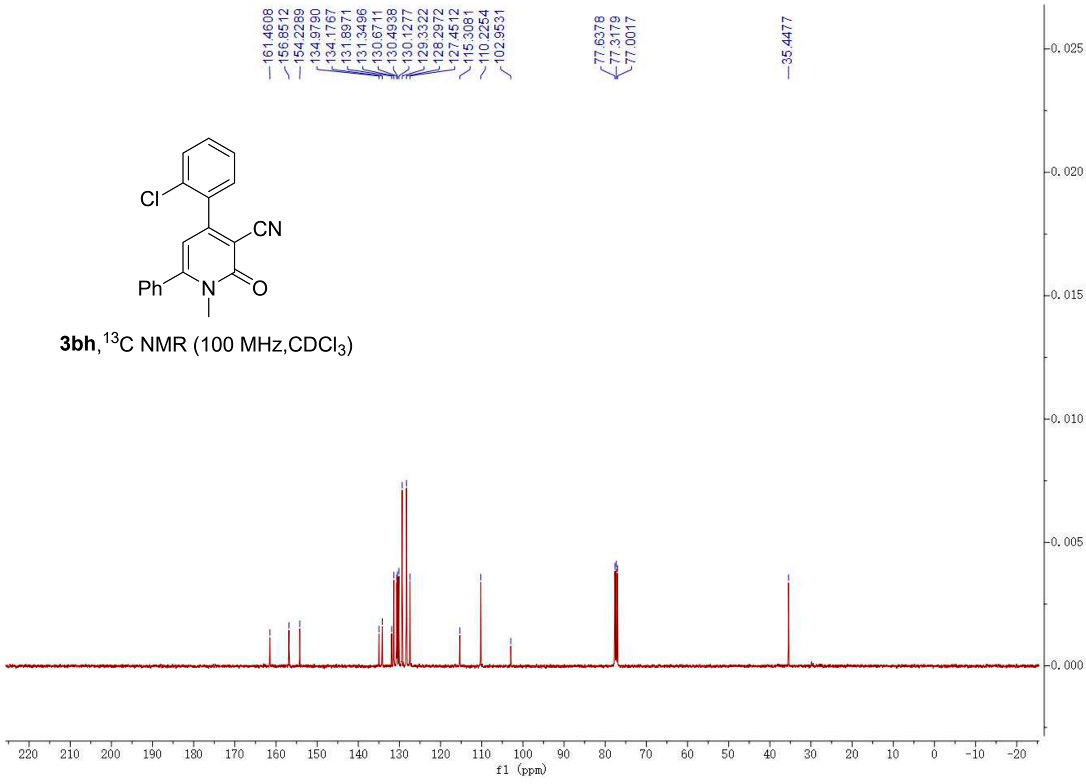
161.4608
156.8512
154.2289
134.9790
134.1767
131.8971
131.3496
130.6711
130.4938
130.1277
129.3322
128.2972
127.4512
115.3081
110.2254
102.9531
77.6378
77.3179
77.0017
35.4477
Cl
CN
Ph
N
O
3bh,^{13}C NMR (100 MHz,$CDCl_3$)
f1 (ppm)

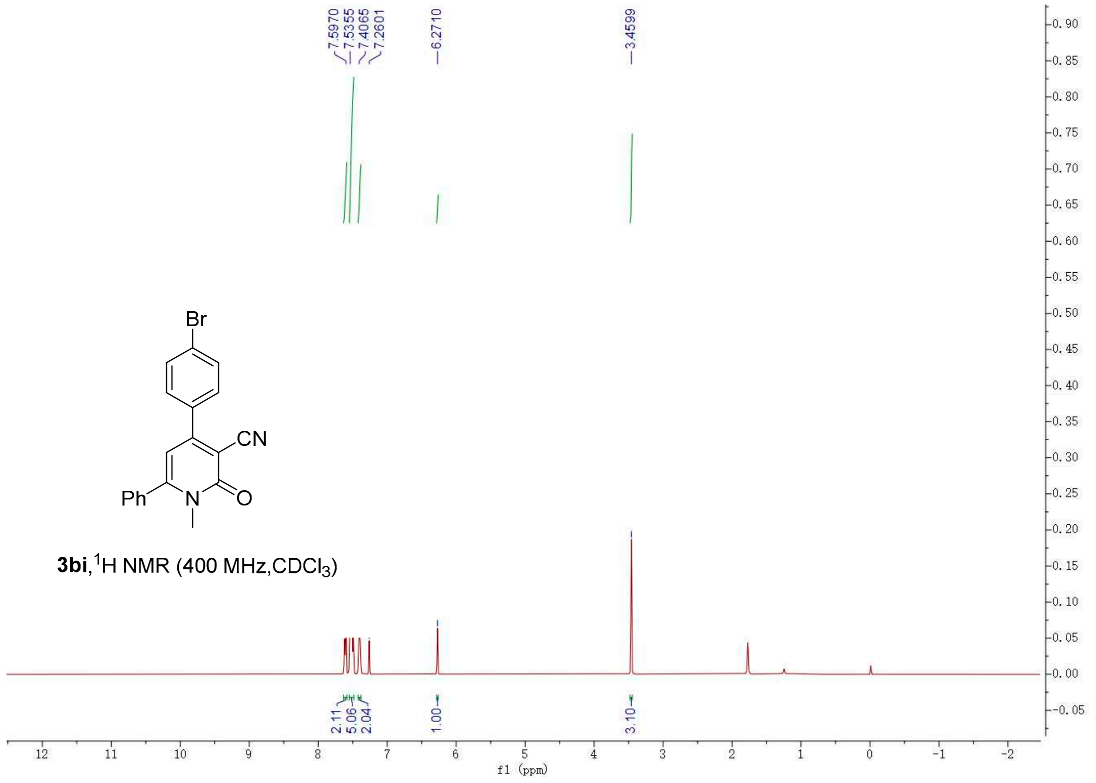

3bi,^{1}H NMR (400 MHz,$CDCl_3$)

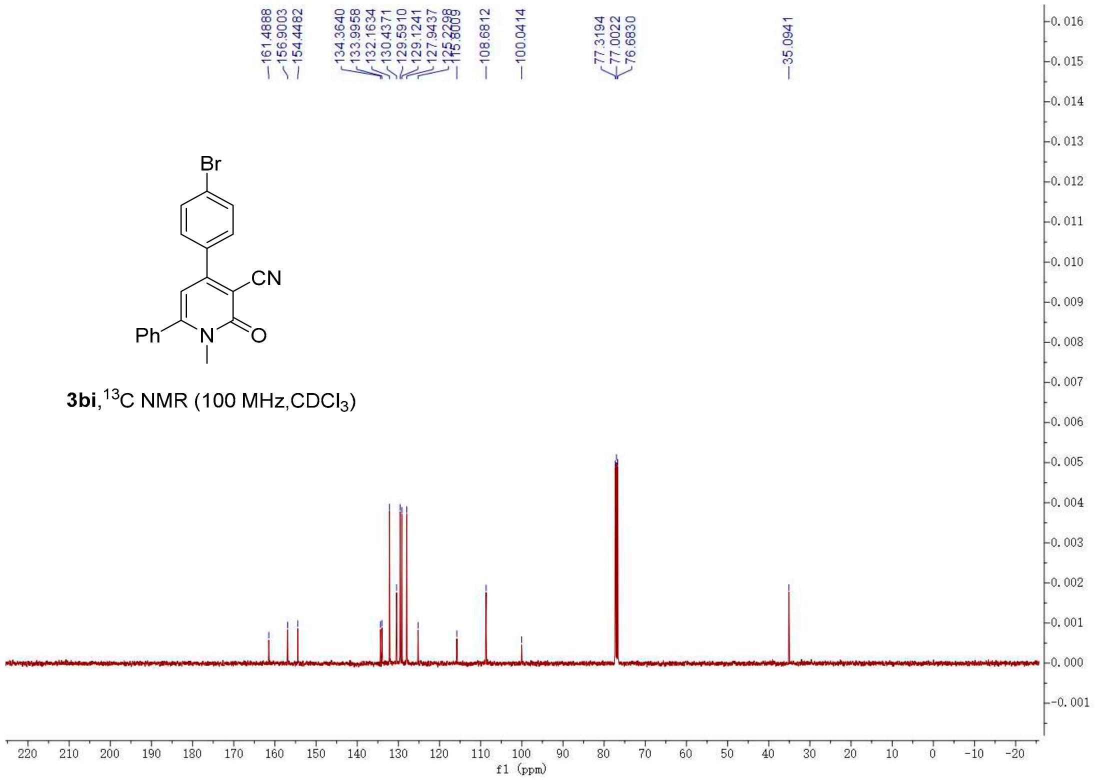

3bi,^{13}C NMR (100 MHz,$CDCl_3$)

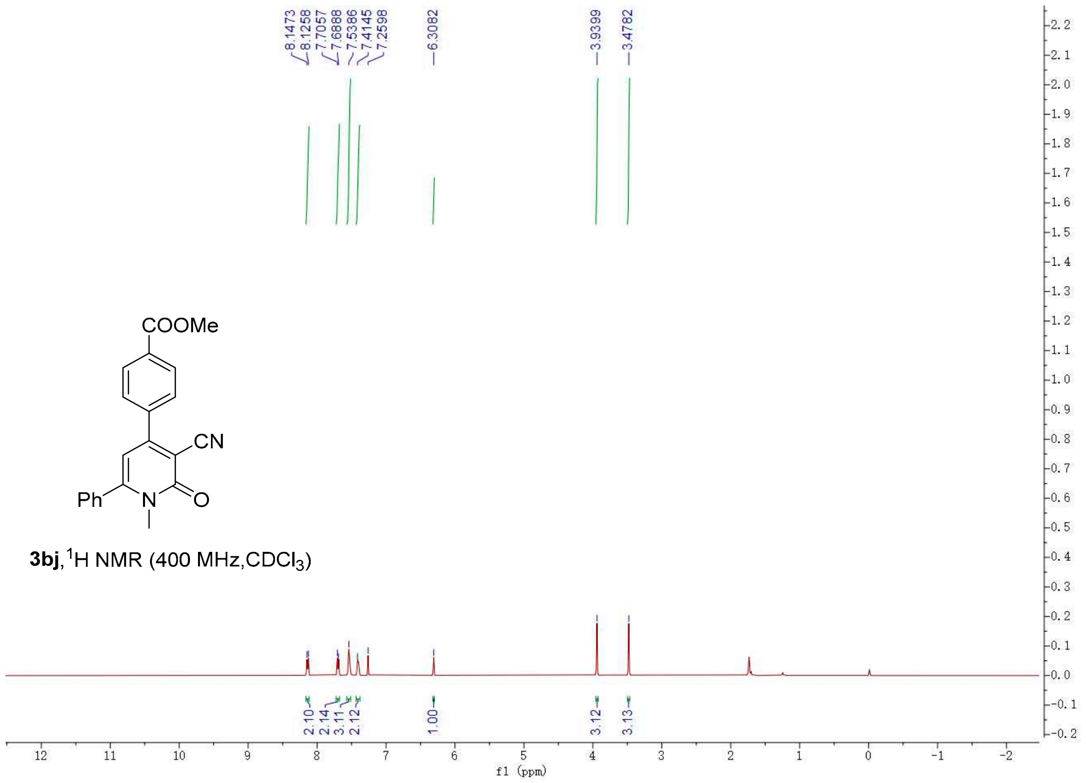

3bj, ^{1}H NMR (400 MHz, $CDCl_3$)

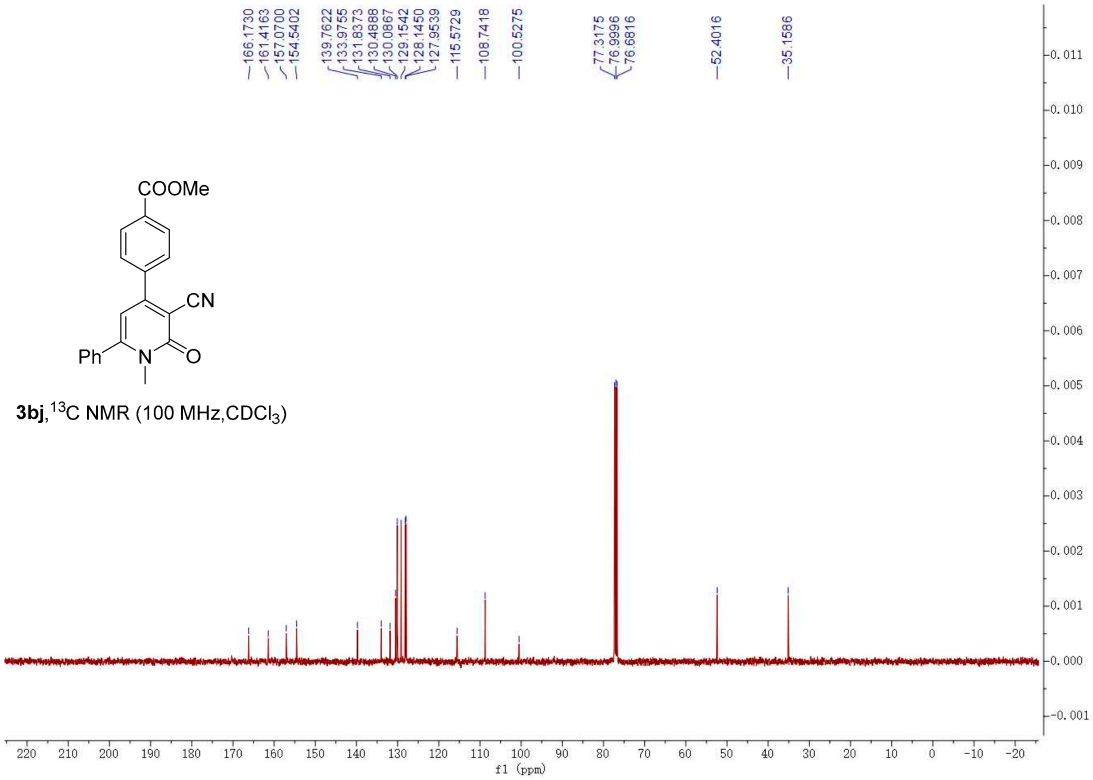

3bj, ^{13}C NMR (100 MHz, $CDCl_3$)

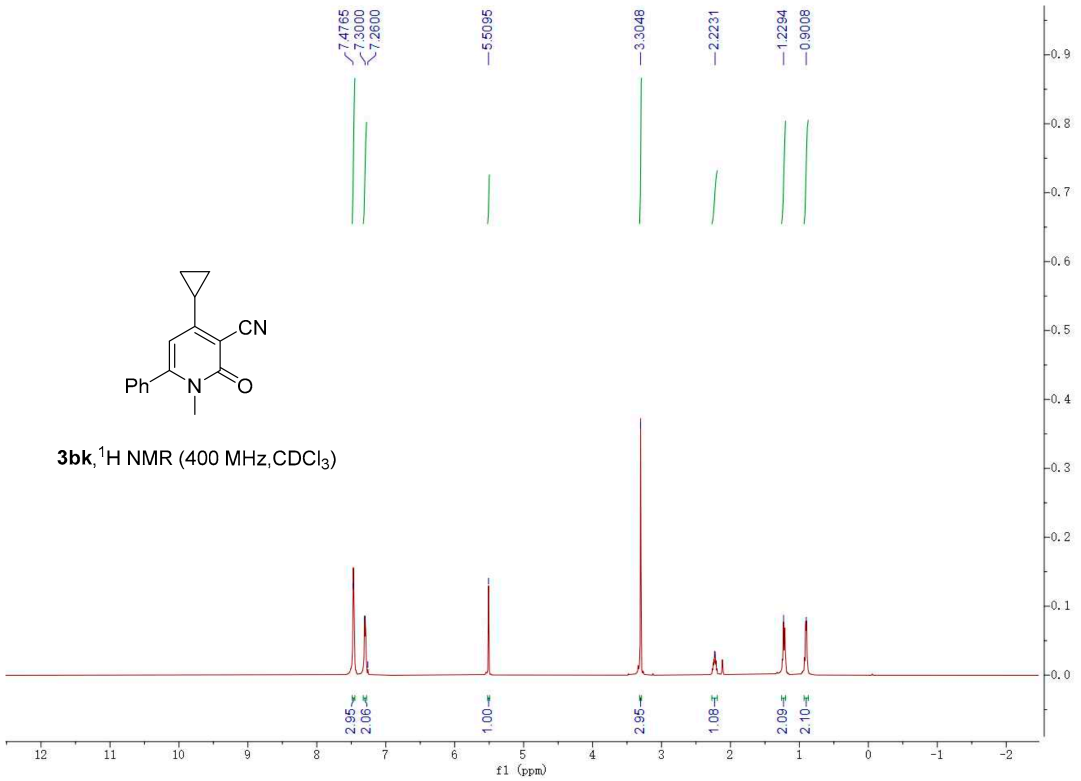
7.4765
7.3000
7.2600
5.5095
3.3048
2.2231
1.2294
0.9008
CN
Ph
N
O
3bk, 1H NMR (400 MHz,CDCl3)
2.95
2.06
1.00
2.95
1.08
2.09
2.10
f1 (ppm)

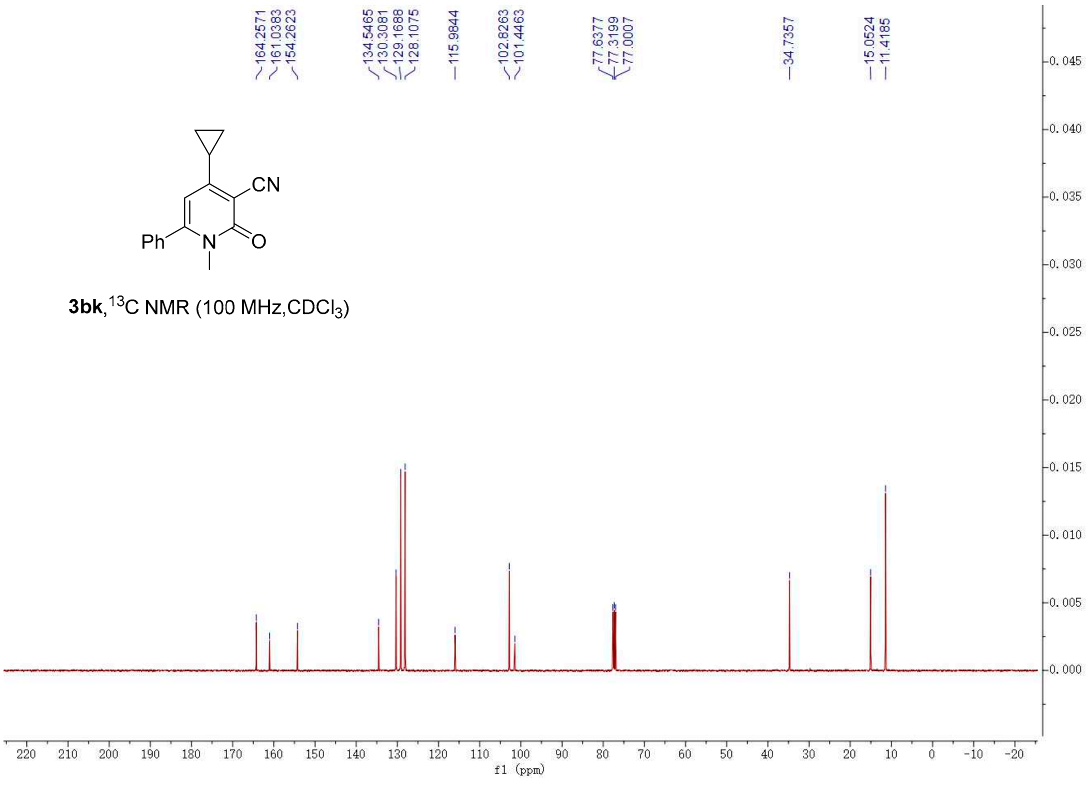
164.2571
161.0383
154.2623
134.5465
130.3081
129.1688
128.1075
115.9844
102.8263
101.4463
77.6377
77.3199
77.0007
34.7357
15.0524
11.4185
CN
Ph
N
O
3bk, 13C NMR (100 MHz,CDCl3)
f1 (ppm)

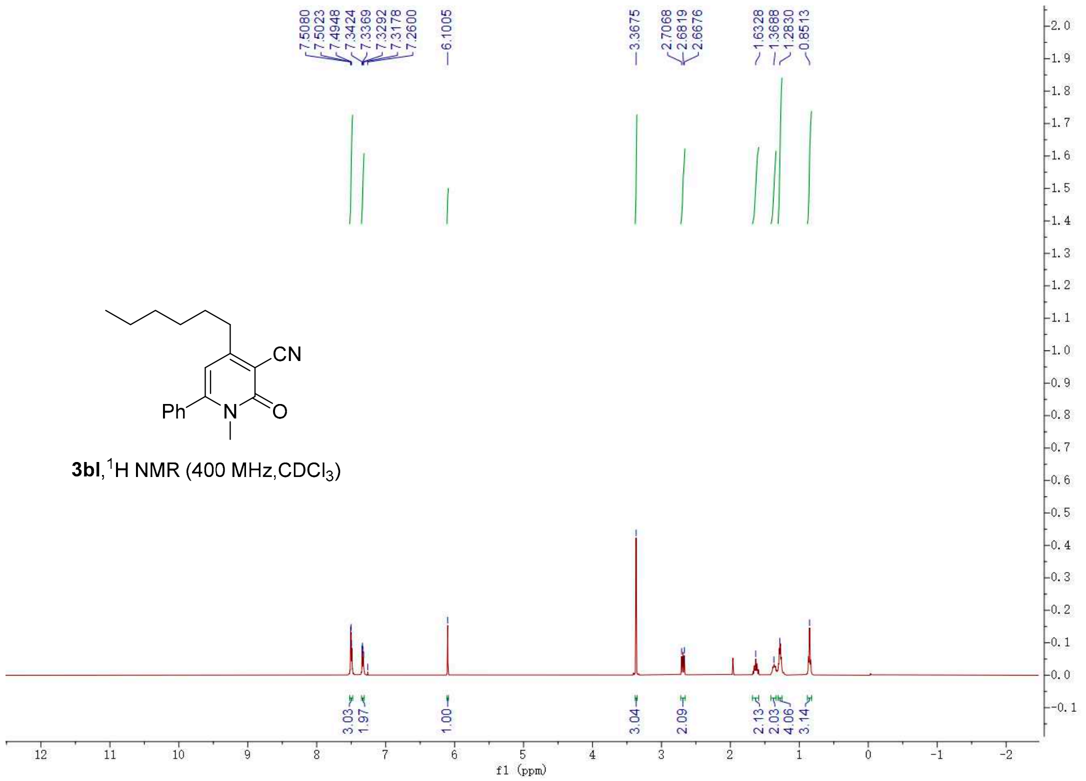

3bl, ^{1}H NMR (400 MHz, $CDCl_3$)

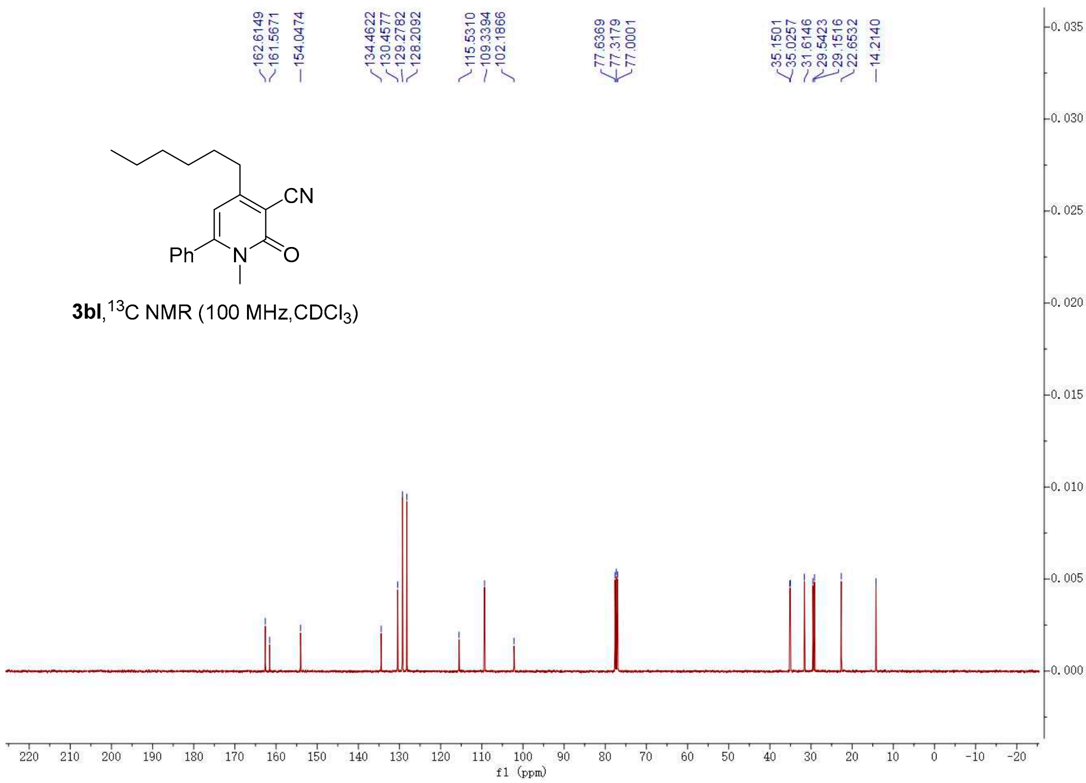

3bl, ^{13}C NMR (100 MHz, $CDCl_3$)

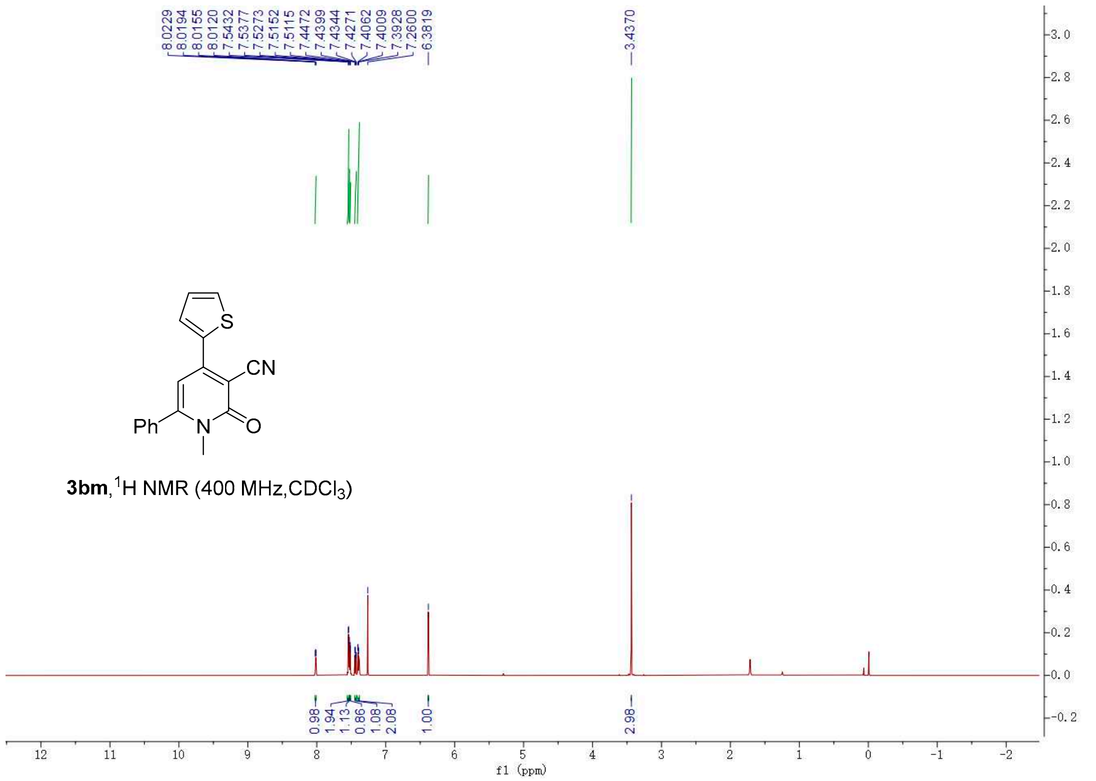

3bm,^{1}H NMR (400 MHz,$CDCl_3$)

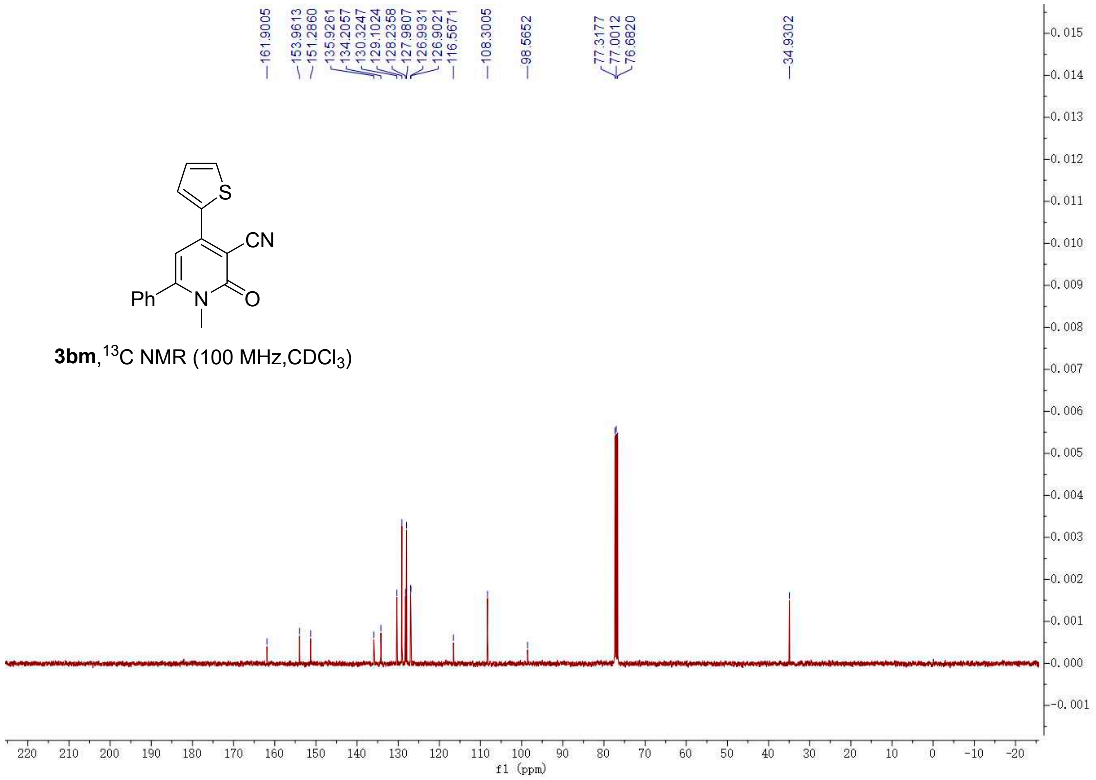

3bm,^{13}C NMR (100 MHz,$CDCl_3$)

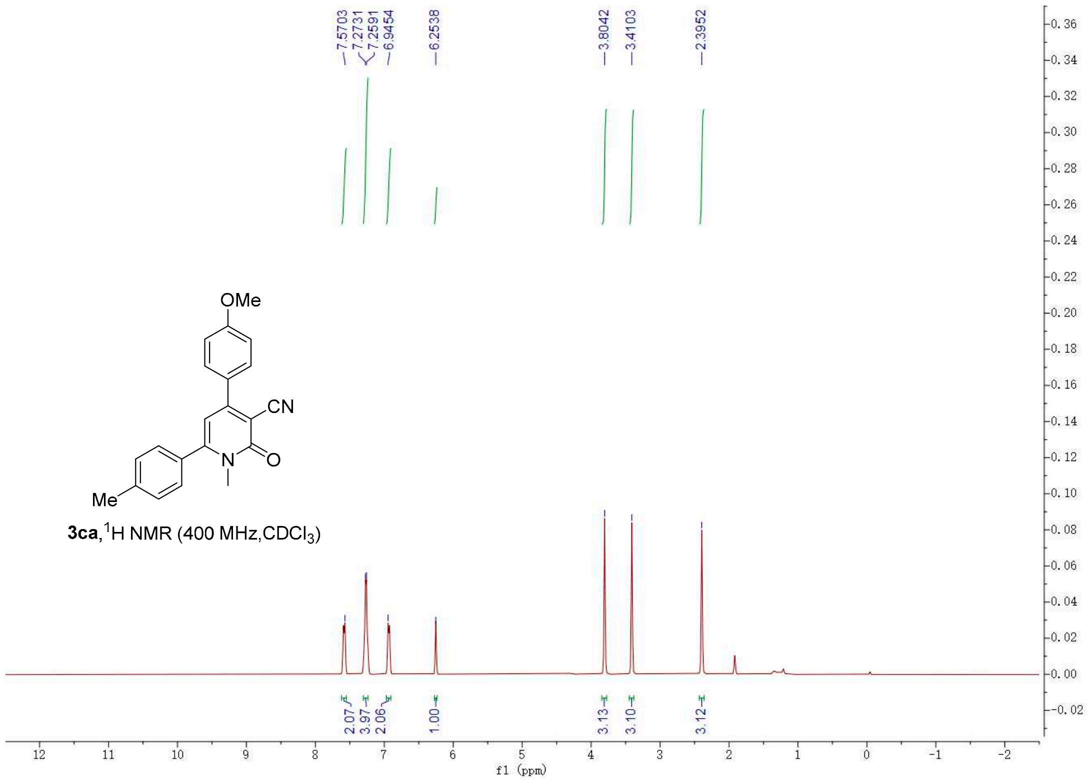
7.5703
7.2731
7.2591
6.9454
6.2538
3.8042
3.4103
2.3952
OMe
CN
N
O
Me
3ca, ^{1}H NMR (400 MHz, $CDCl_3$)
2.07
3.97
2.06
1.00
3.13
3.10
3.12
f1 (ppm)

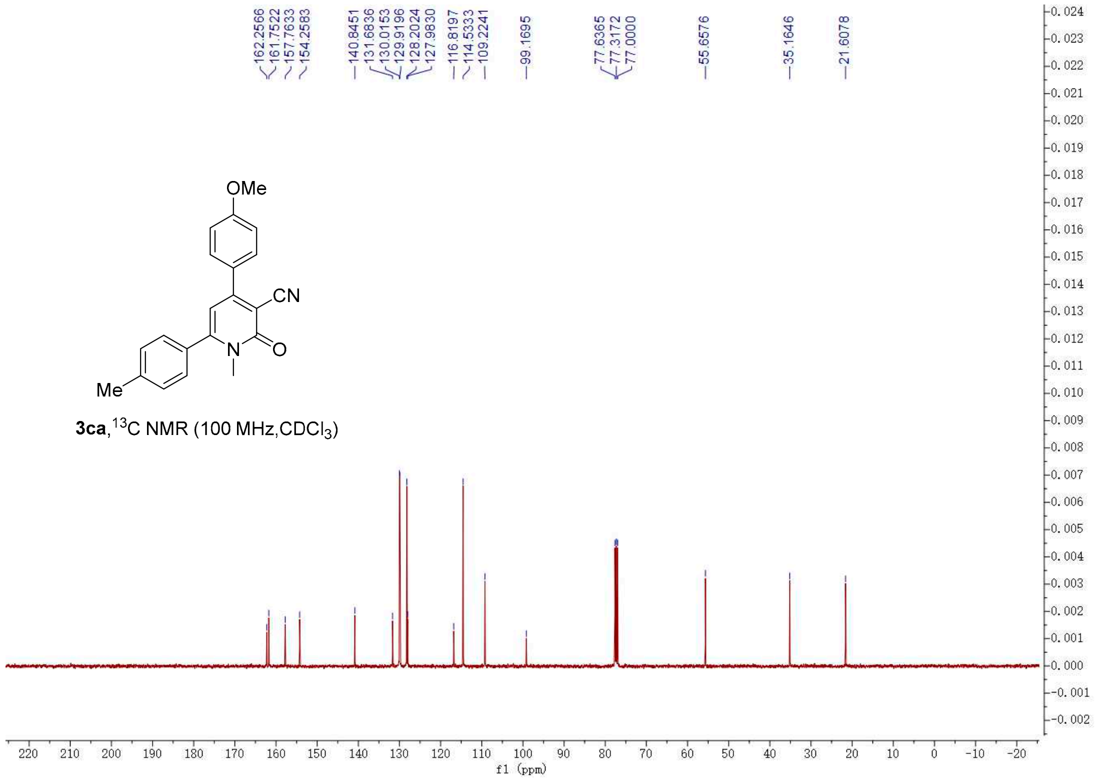
162.2566
161.7522
157.7633
154.2583
140.8451
131.6836
130.0153
129.9196
128.2024
127.9830
116.8197
114.5333
109.2241
99.1695
77.6365
77.3172
77.0000
55.6576
35.1646
21.6078
OMe
CN
N
O
Me
3ca, ^{13}C NMR (100 MHz, $CDCl_3$)
f1 (ppm)

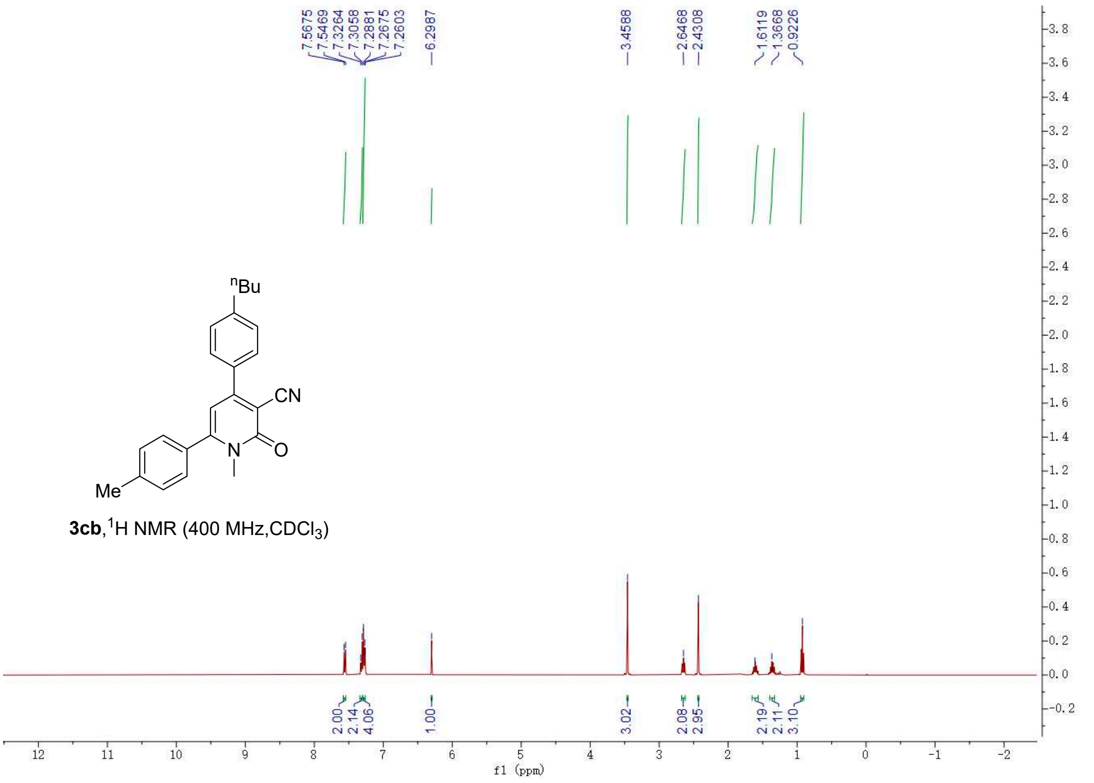

3cb, ^{1}H NMR (400 MHz, $CDCl_3$)

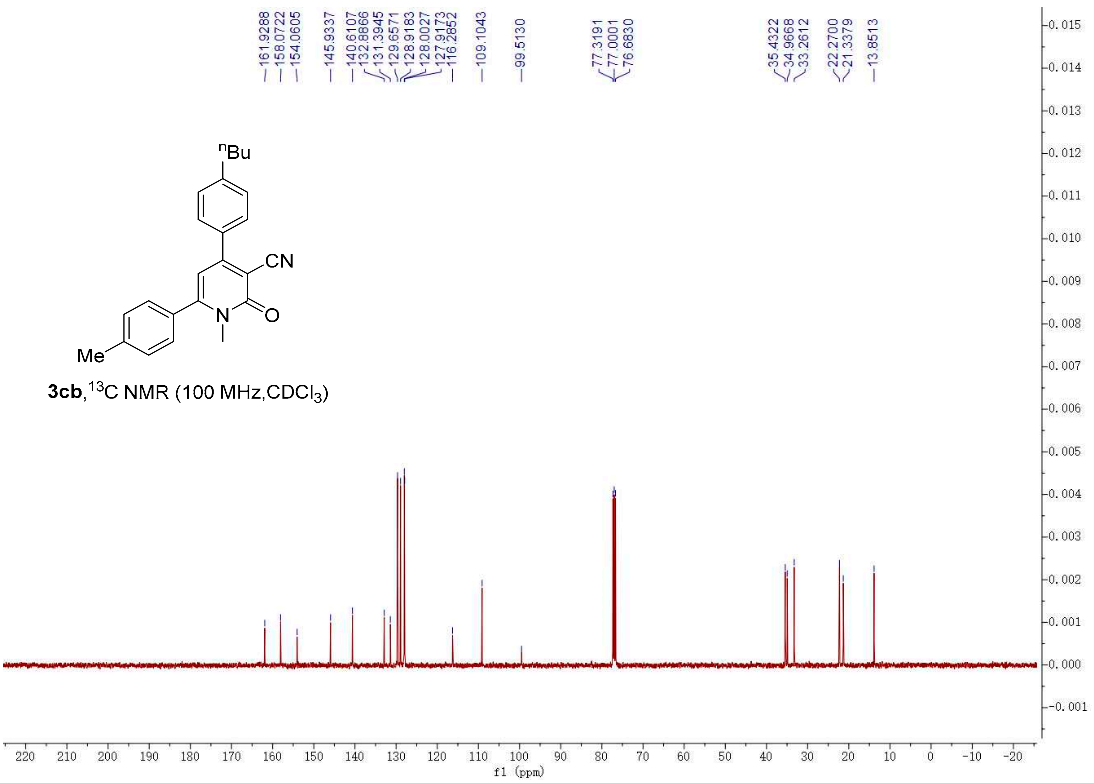

3cb, ^{13}C NMR (100 MHz, $CDCl_3$)

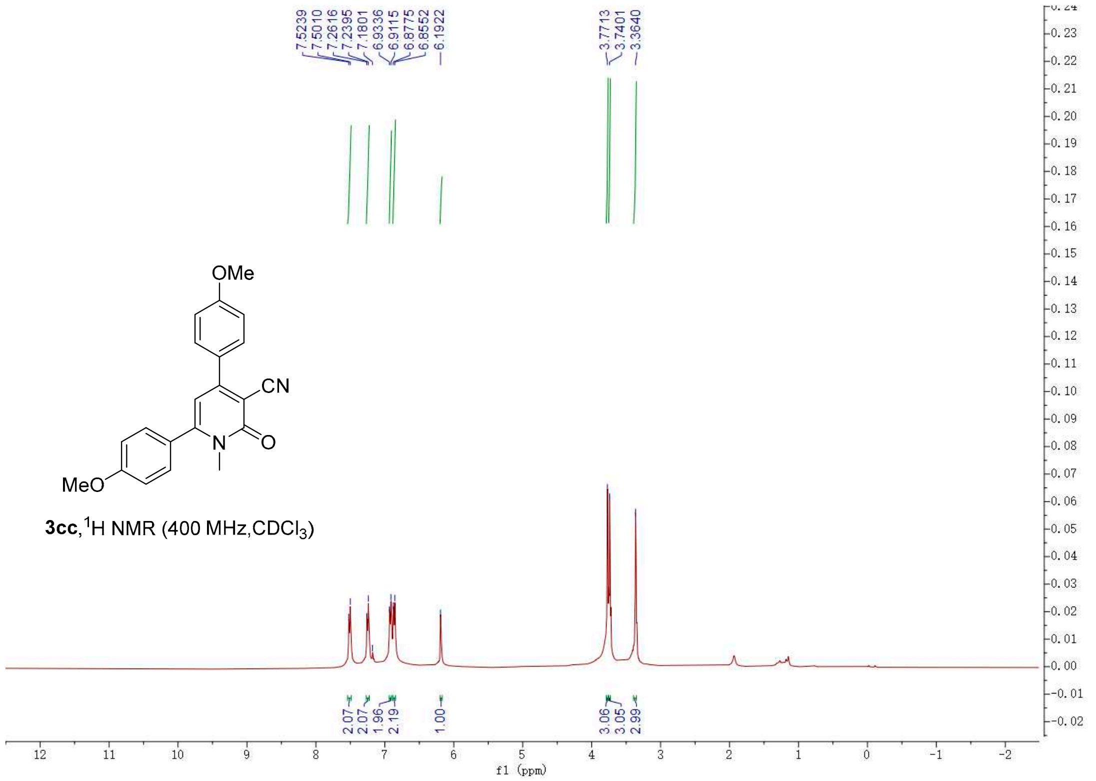

3cc, ^{1}H NMR (400 MHz, $CDCl_3$)

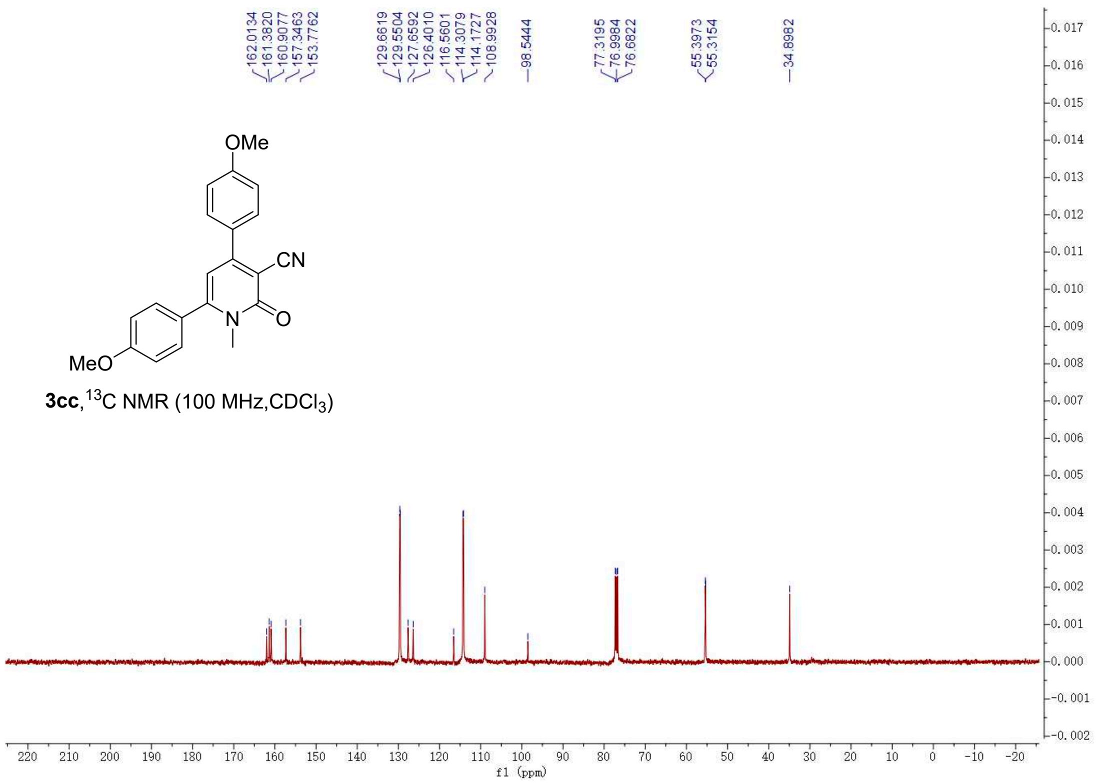

3cc, ^{13}C NMR (100 MHz, $CDCl_3$)

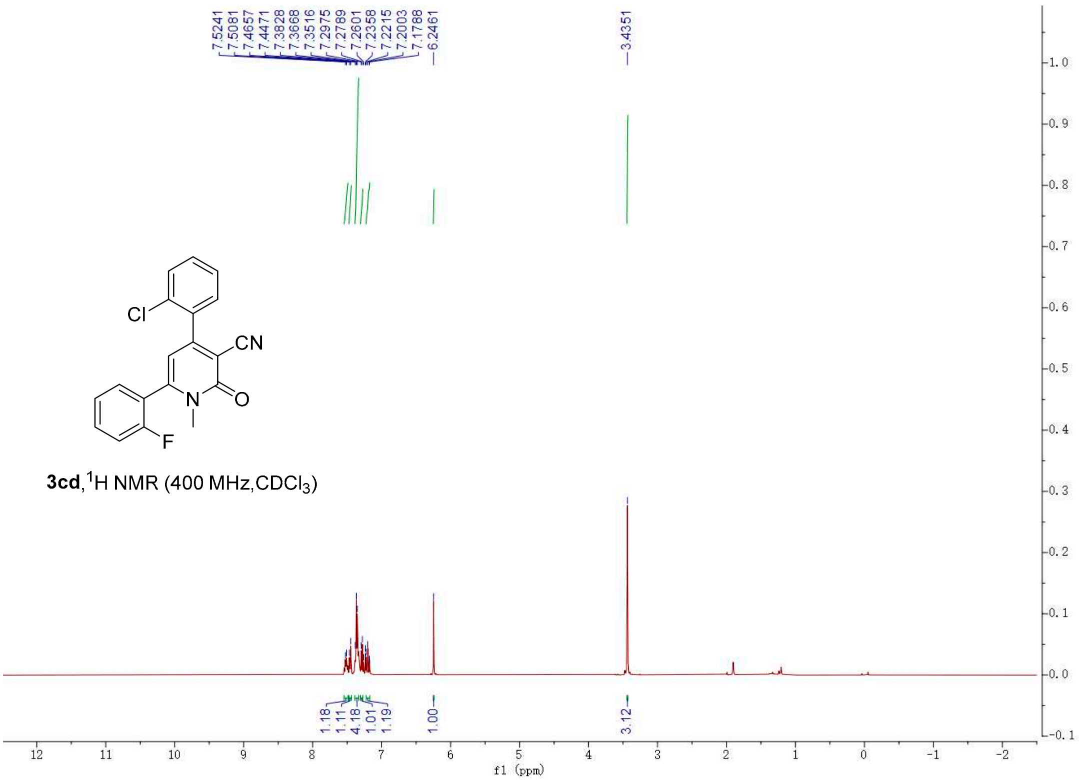

3cd,¹H NMR (400 MHz,$CDCl_3$)

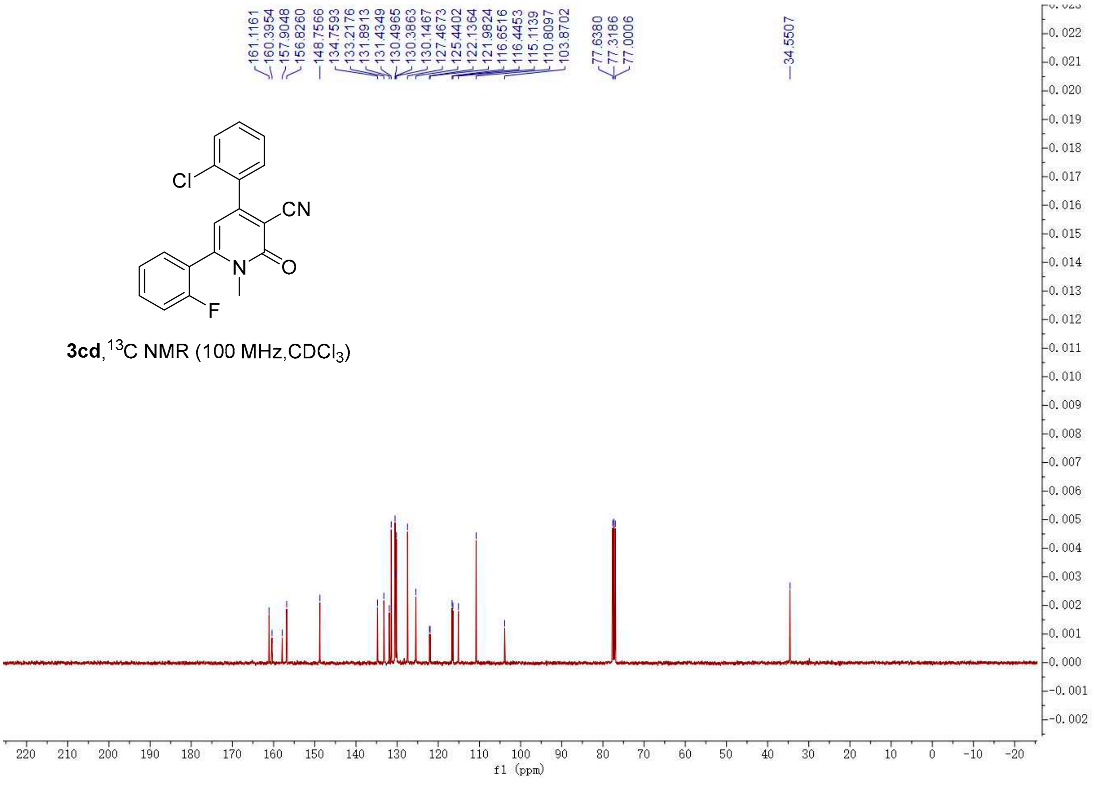

3cd,¹³C NMR (100 MHz,$CDCl_3$)

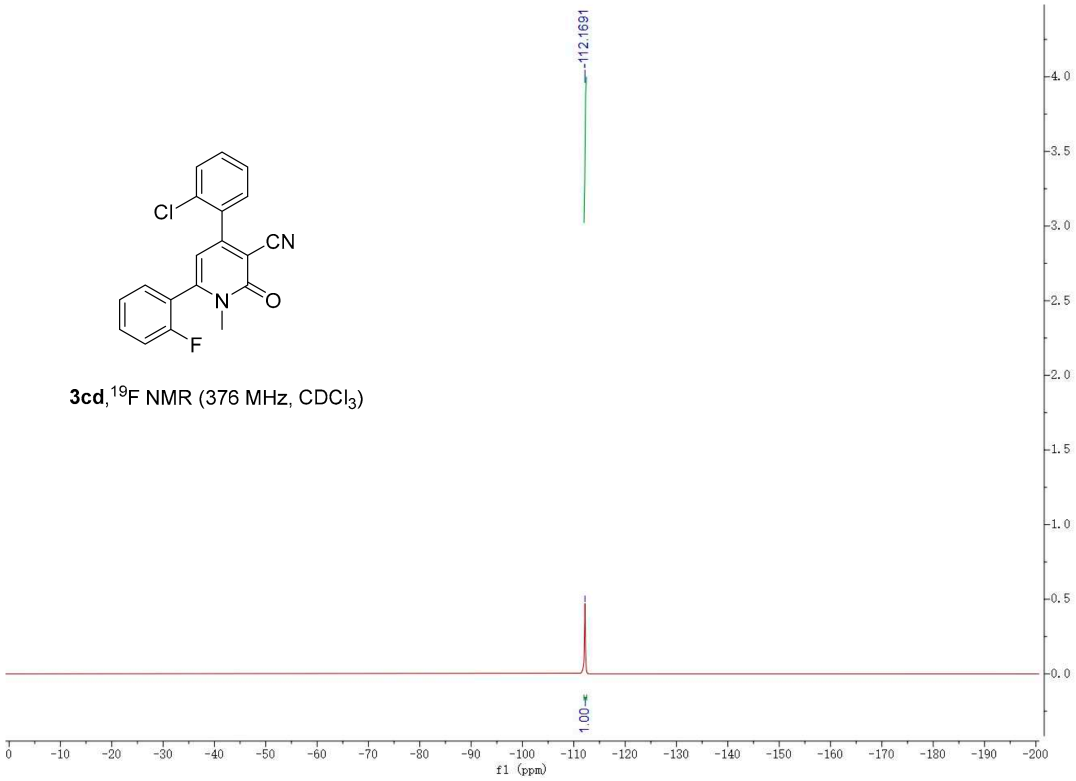

3cd, ^{19}F NMR (376 MHz, CDCl$_3$)

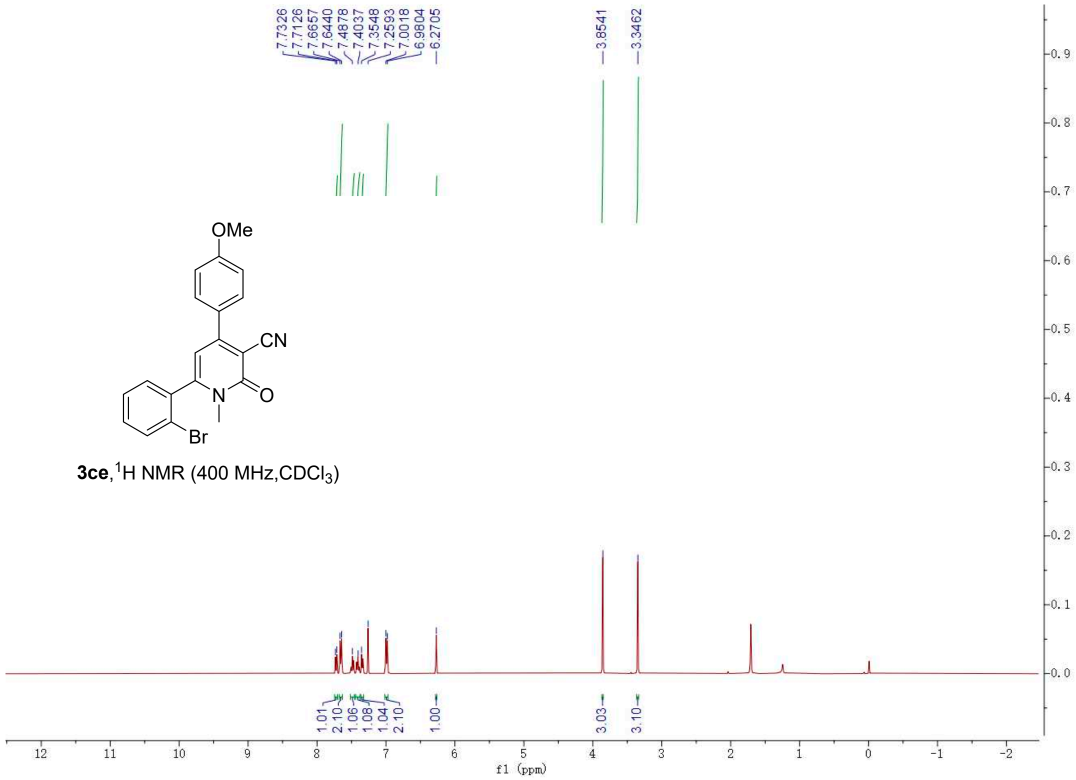

3ce, ^{1}H NMR (400 MHz, CDCl$_3$)

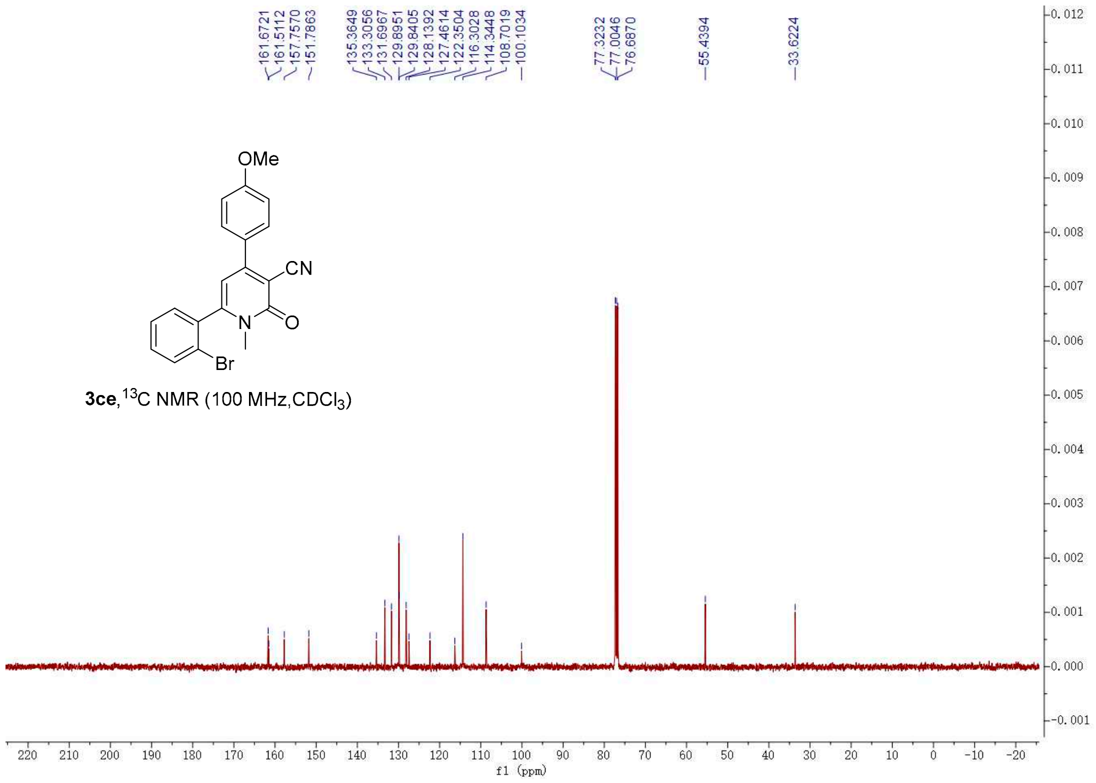

3ce, ^{13}C NMR (100 MHz, $CDCl_3$)

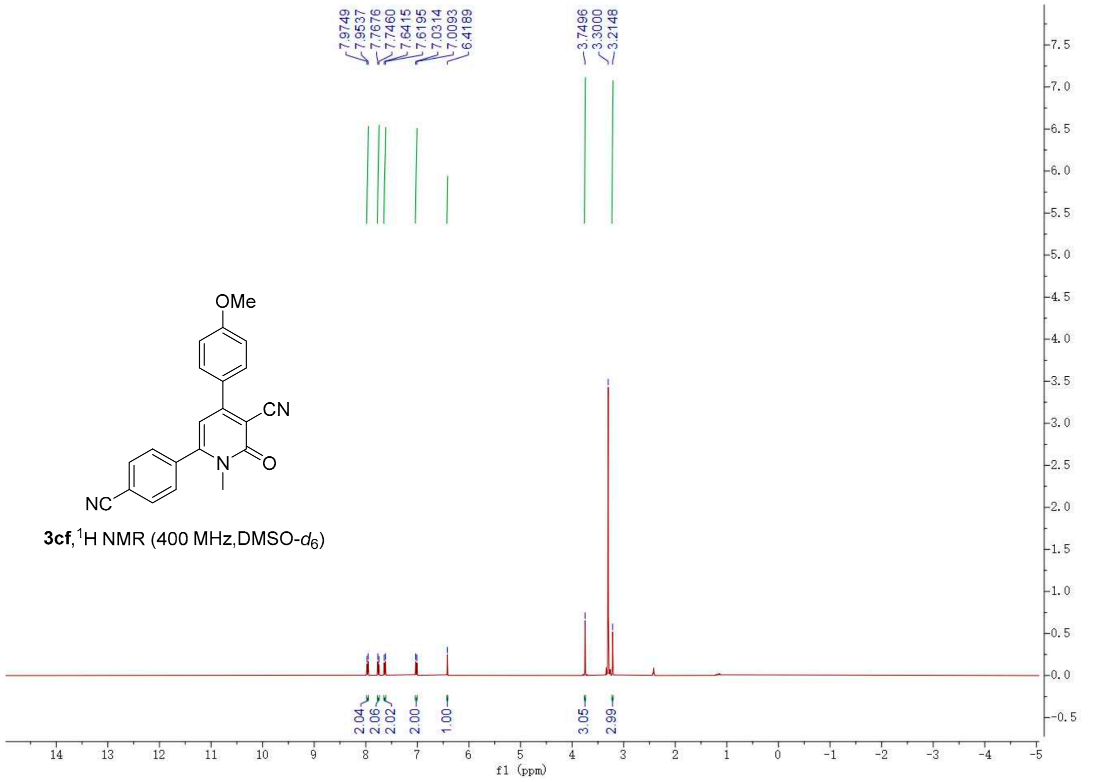

3cf, ^{1}H NMR (400 MHz, DMSO-d_6)

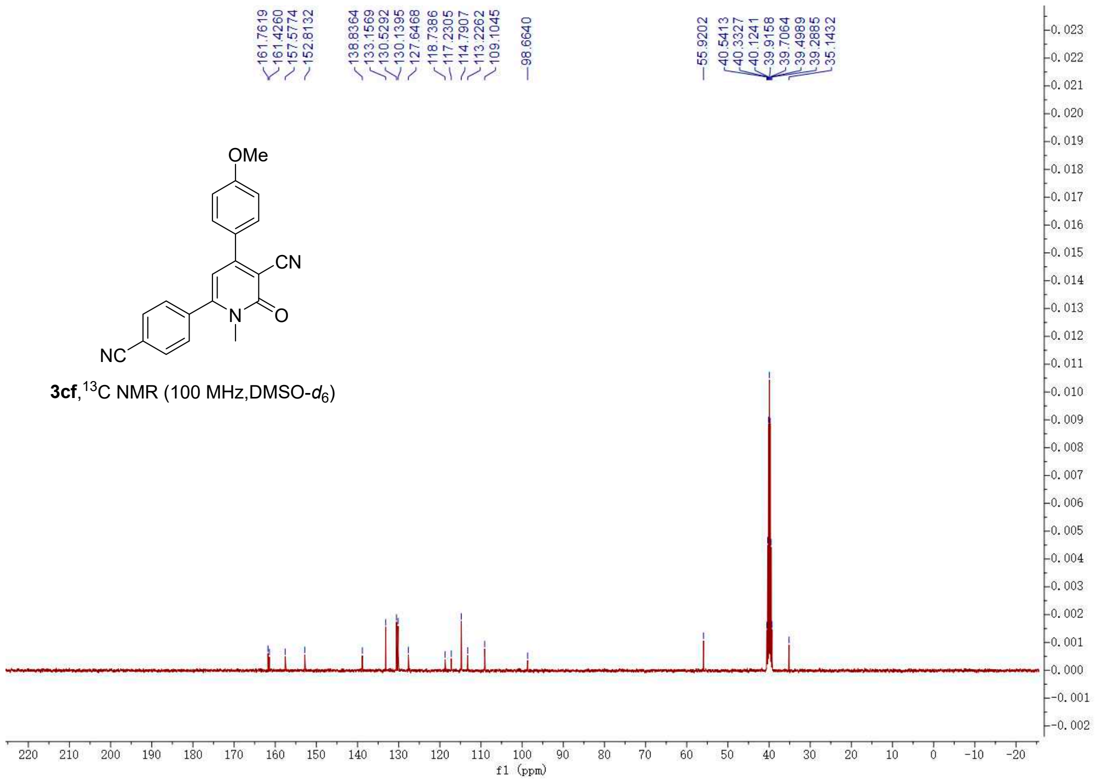

3cf, ^{13}C NMR (100 MHz,DMSO-d_6)

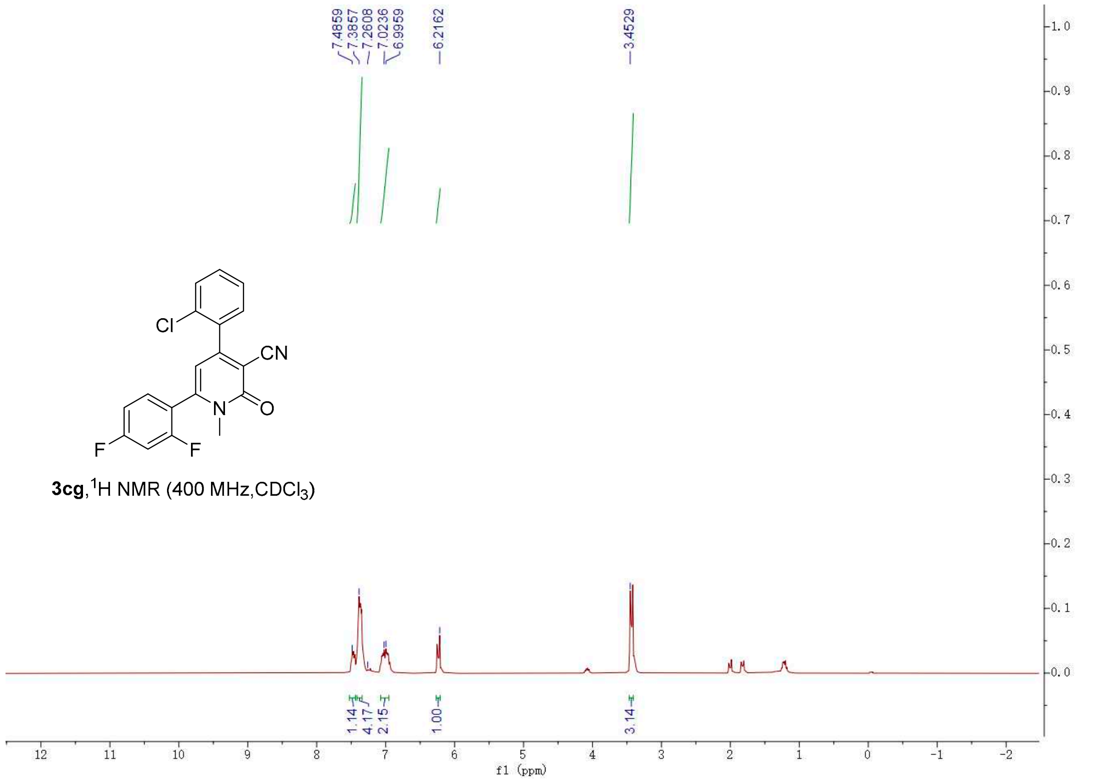

3cg, ^{1}H NMR (400 MHz,CDCl$_3$)

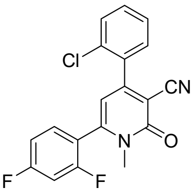

3cg,^{13}C NMR (100 MHz,CDCl$_3$)

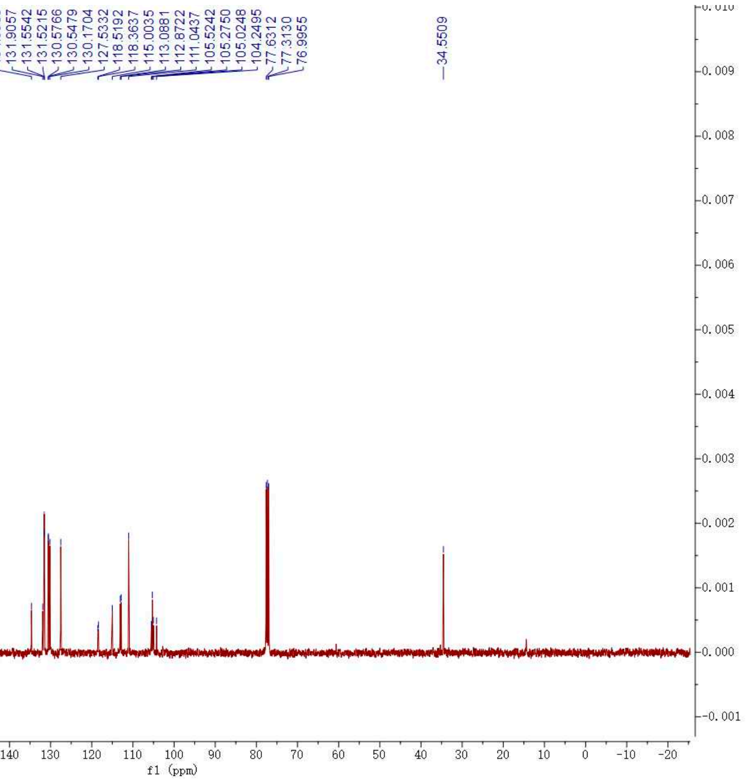

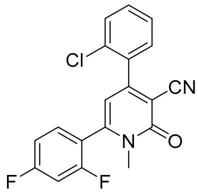

3cg,^{19}F NMR (376 MHz, CDCl$_3$)

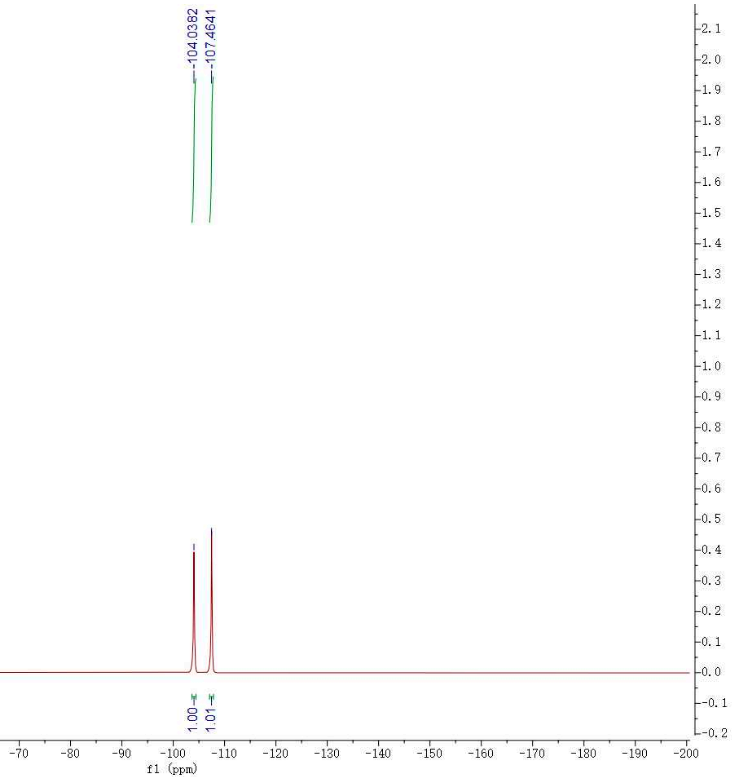

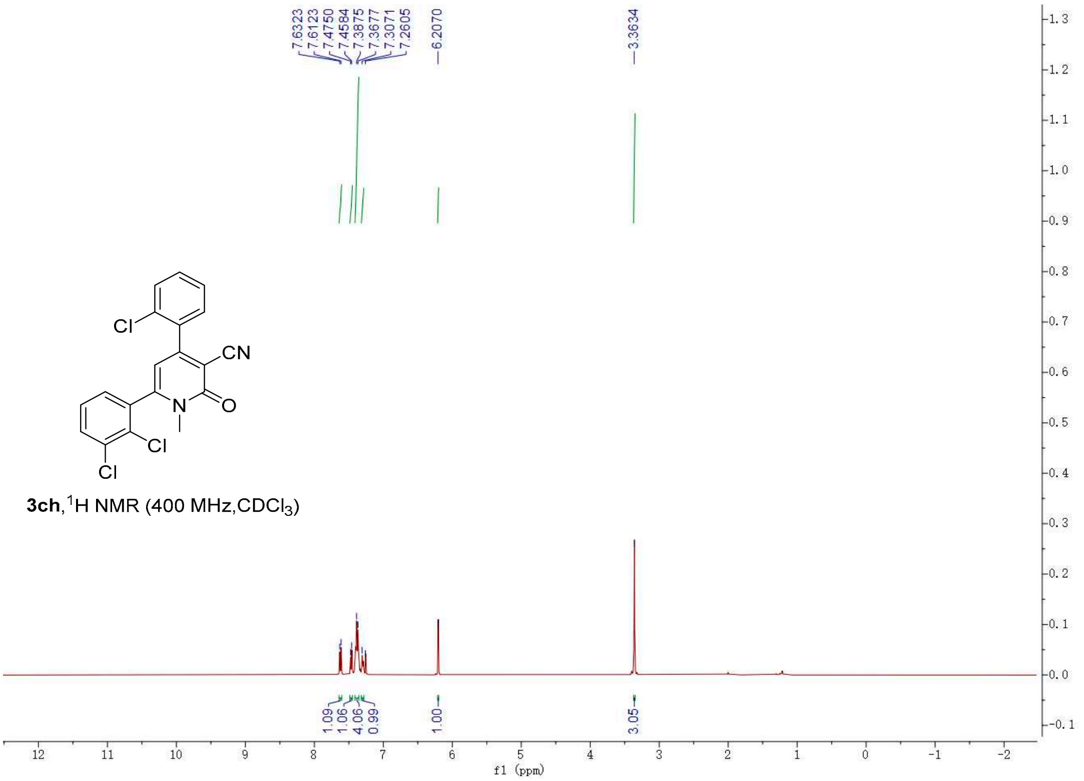

3ch, ^{1}H NMR (400 MHz, $CDCl_3$)

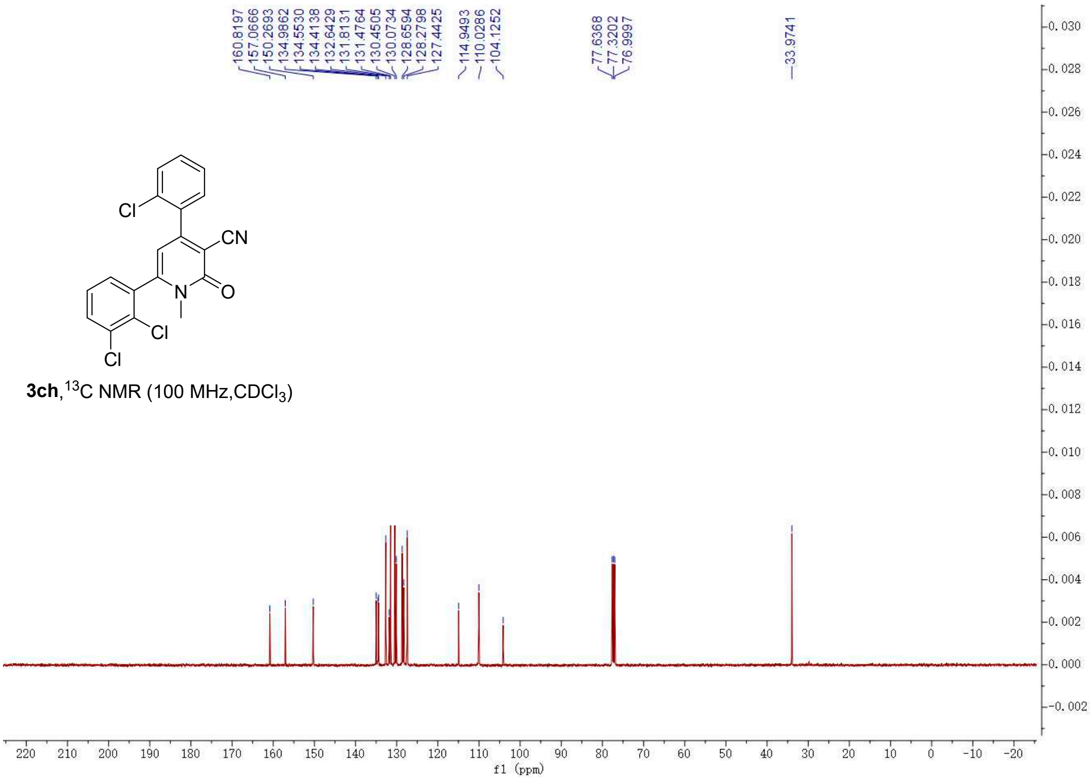

3ch, ^{13}C NMR (100 MHz, $CDCl_3$)

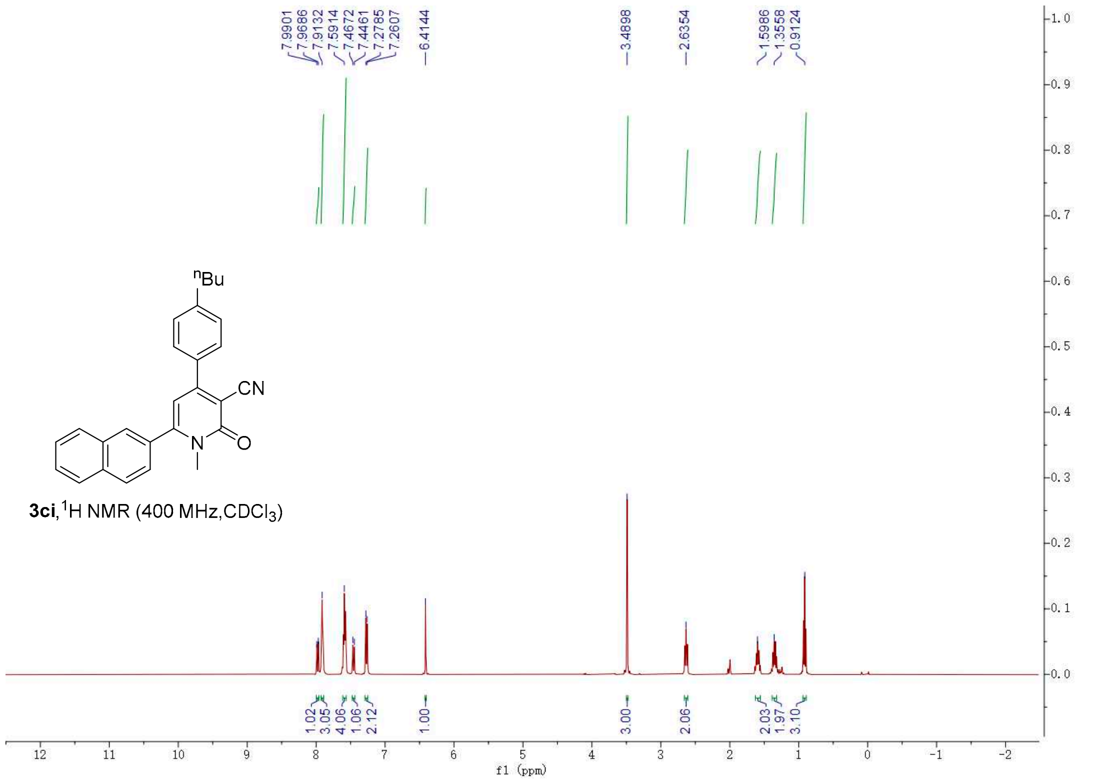

3ci, ^{1}H NMR (400 MHz, $CDCl_3$)

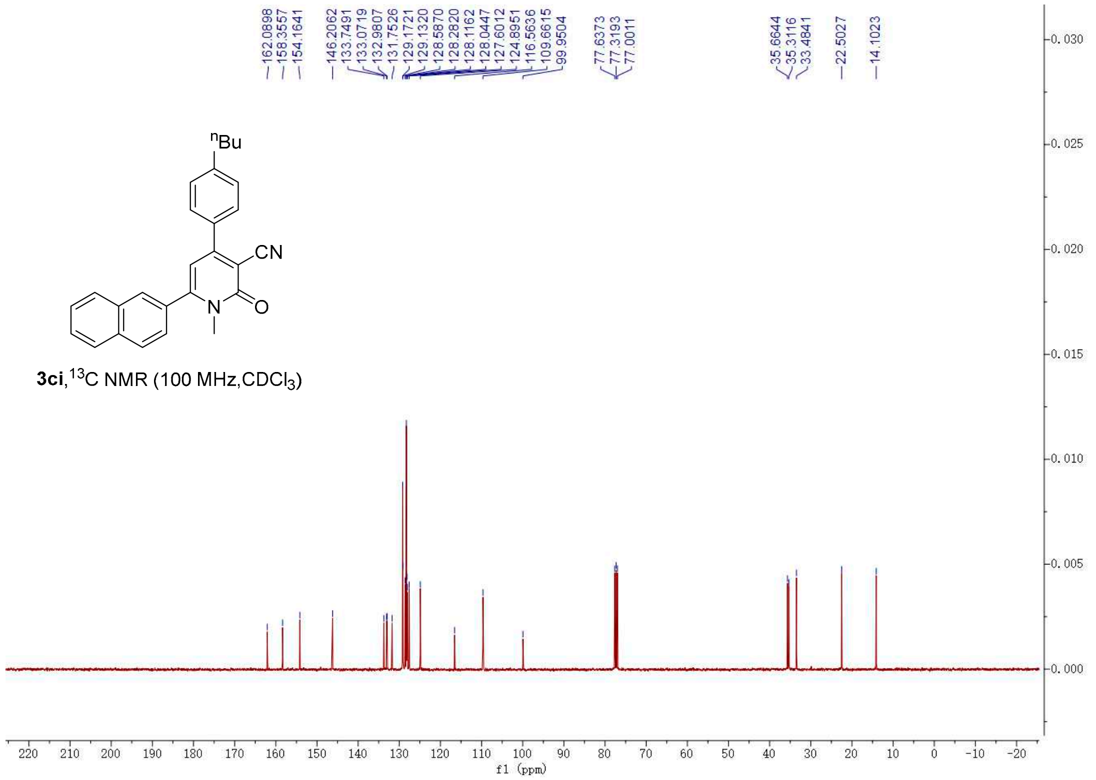

3ci, ^{13}C NMR (100 MHz, $CDCl_3$)

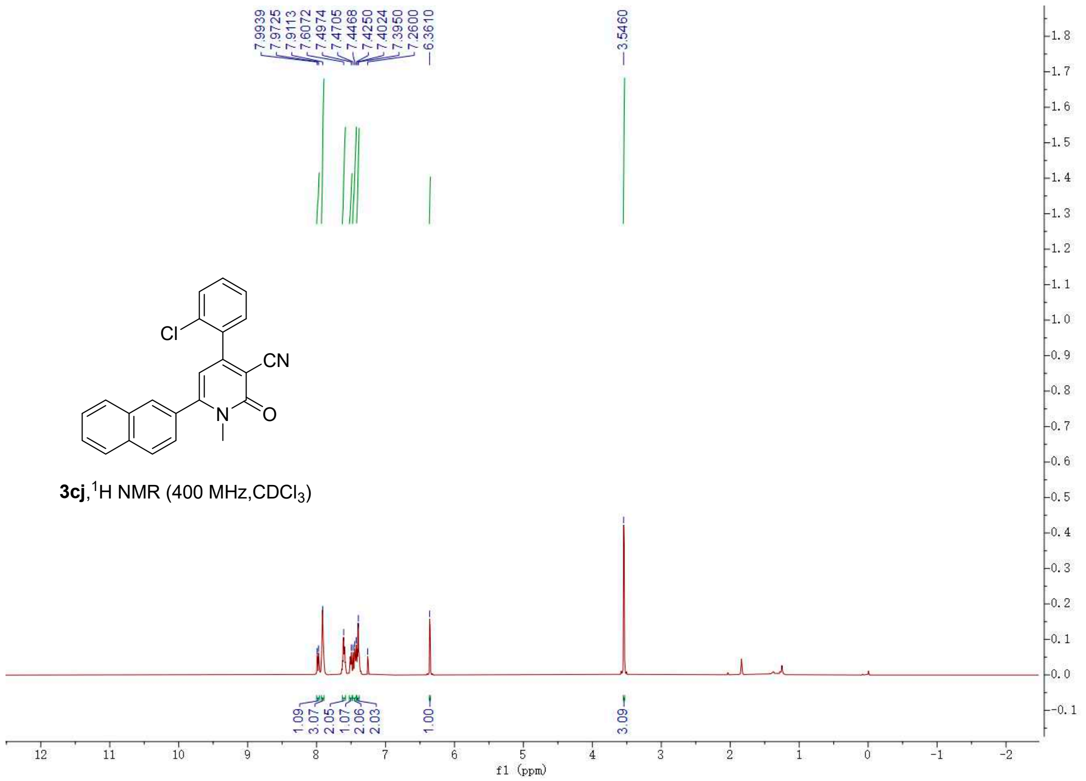

3cj, ^{1}H NMR (400 MHz, $CDCl_3$)

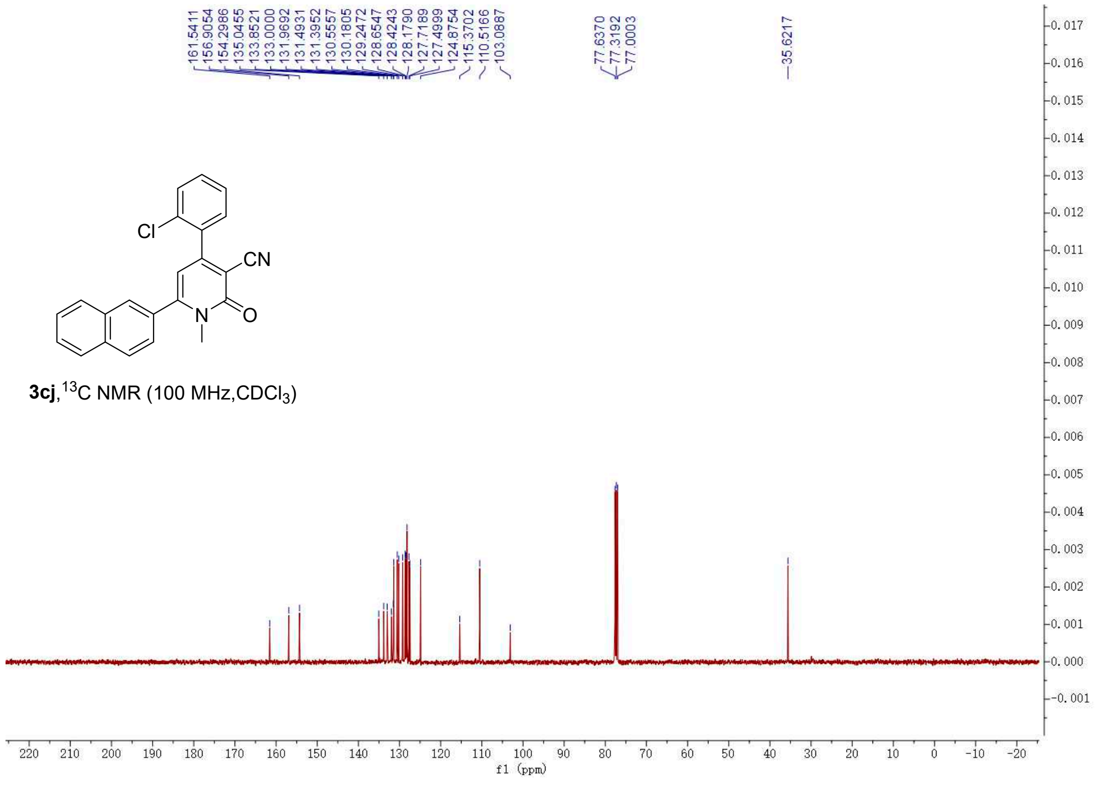

3cj, ^{13}C NMR (100 MHz, $CDCl_3$)

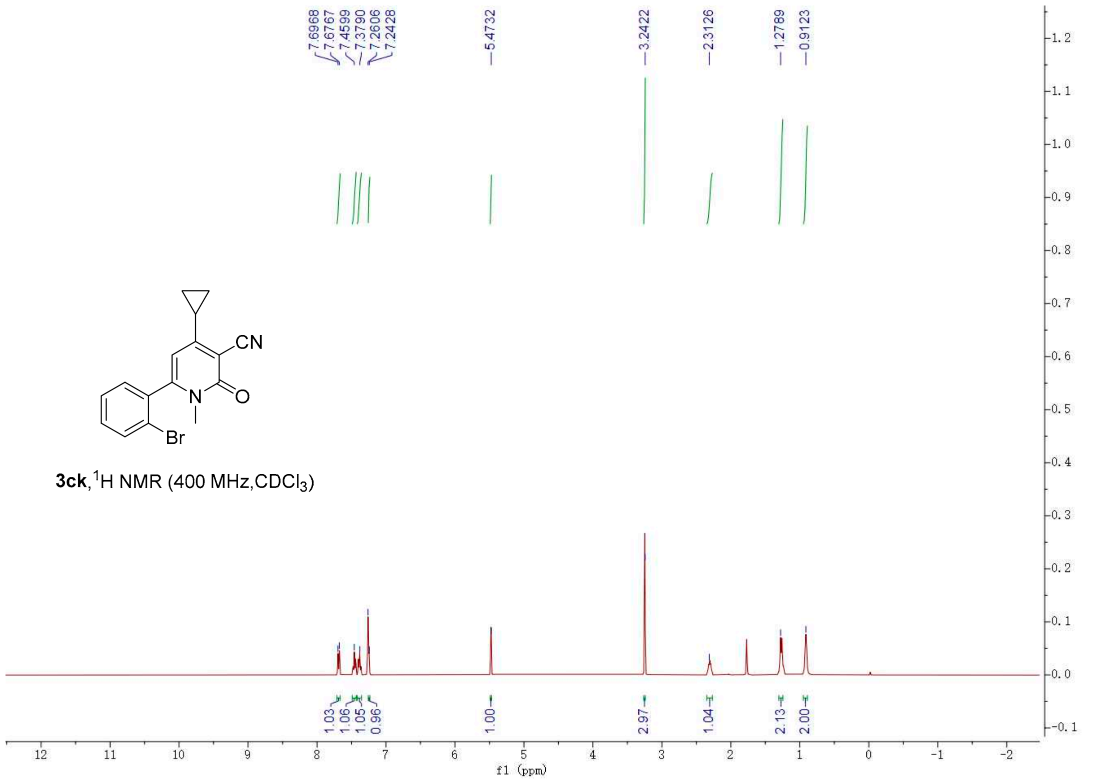

3ck,^{1}H NMR (400 MHz,CDCl$_3$)

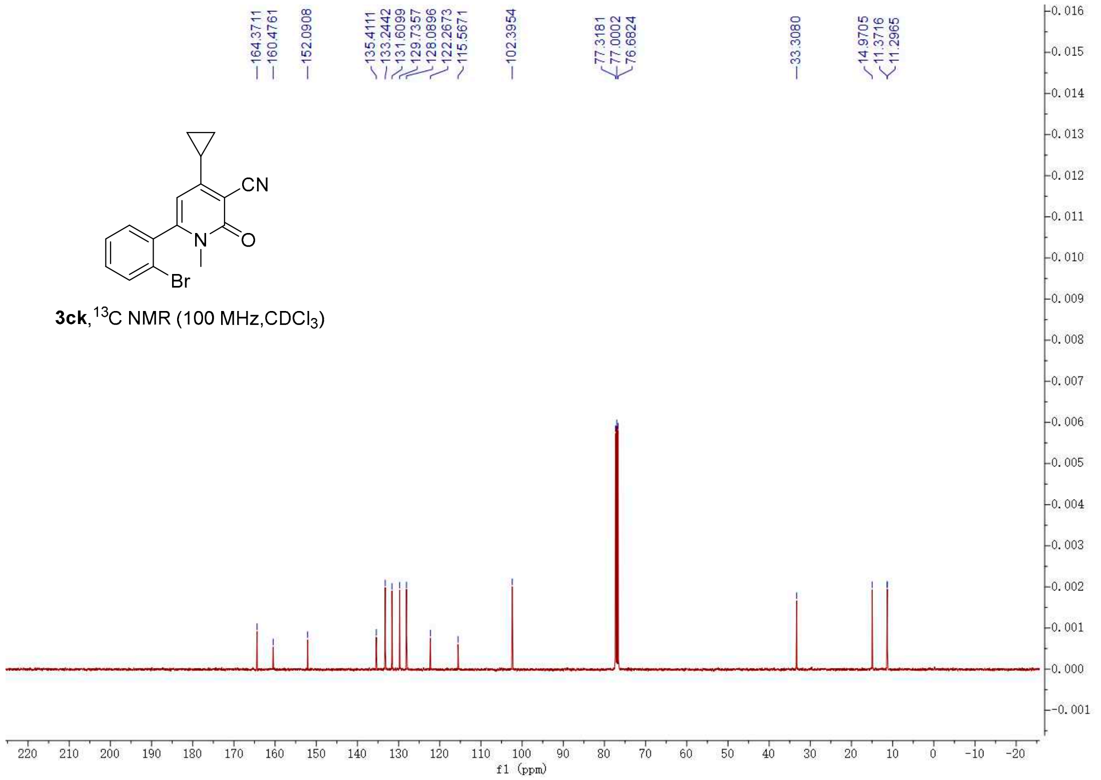

3ck,^{13}C NMR (100 MHz,CDCl$_3$)

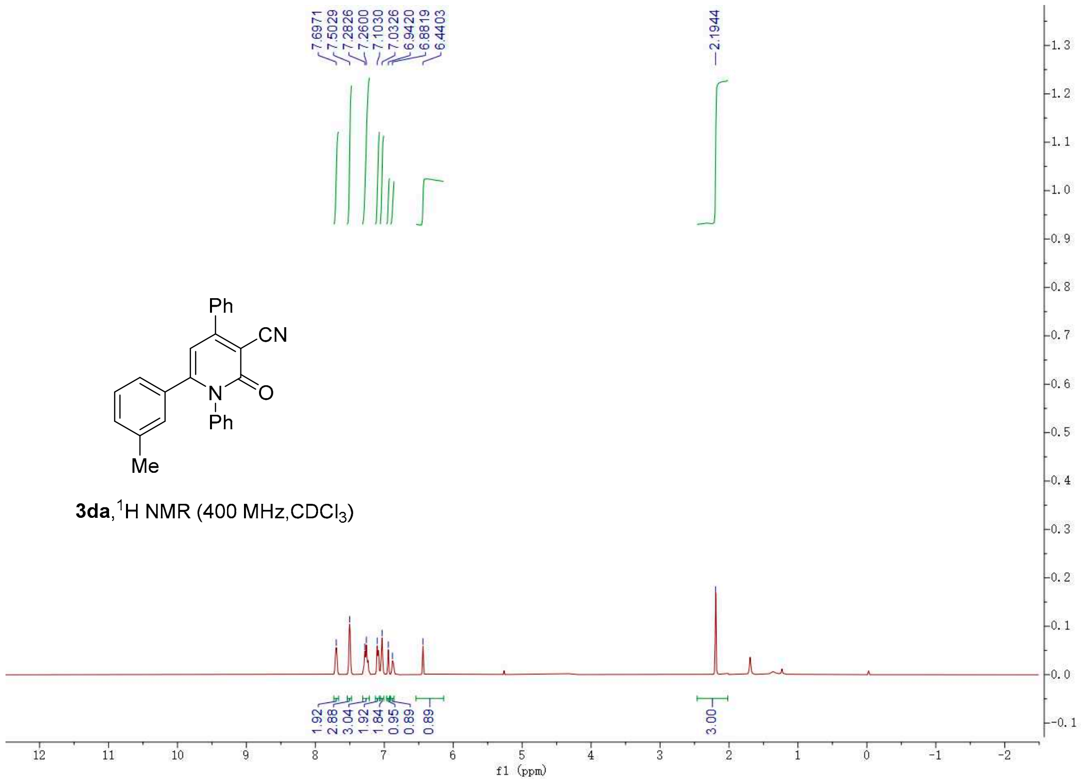

3da, ^{1}H NMR (400 MHz, $CDCl_3$)

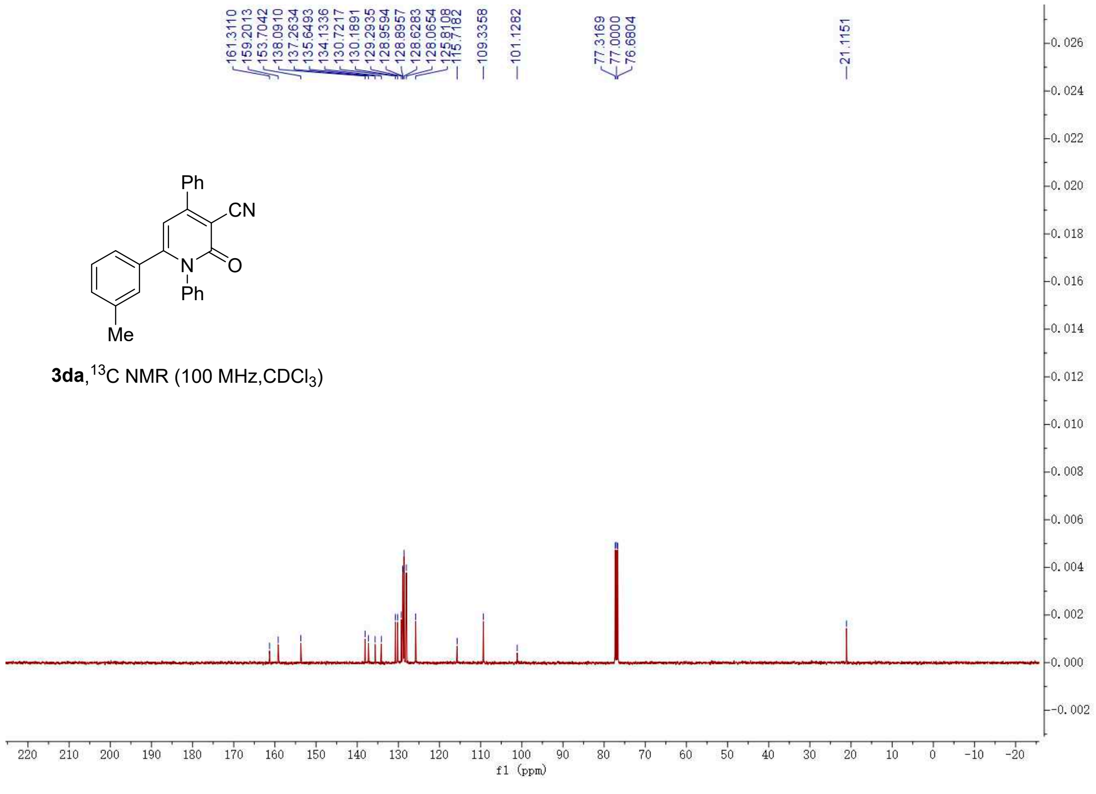

3da, ^{13}C NMR (100 MHz, $CDCl_3$)

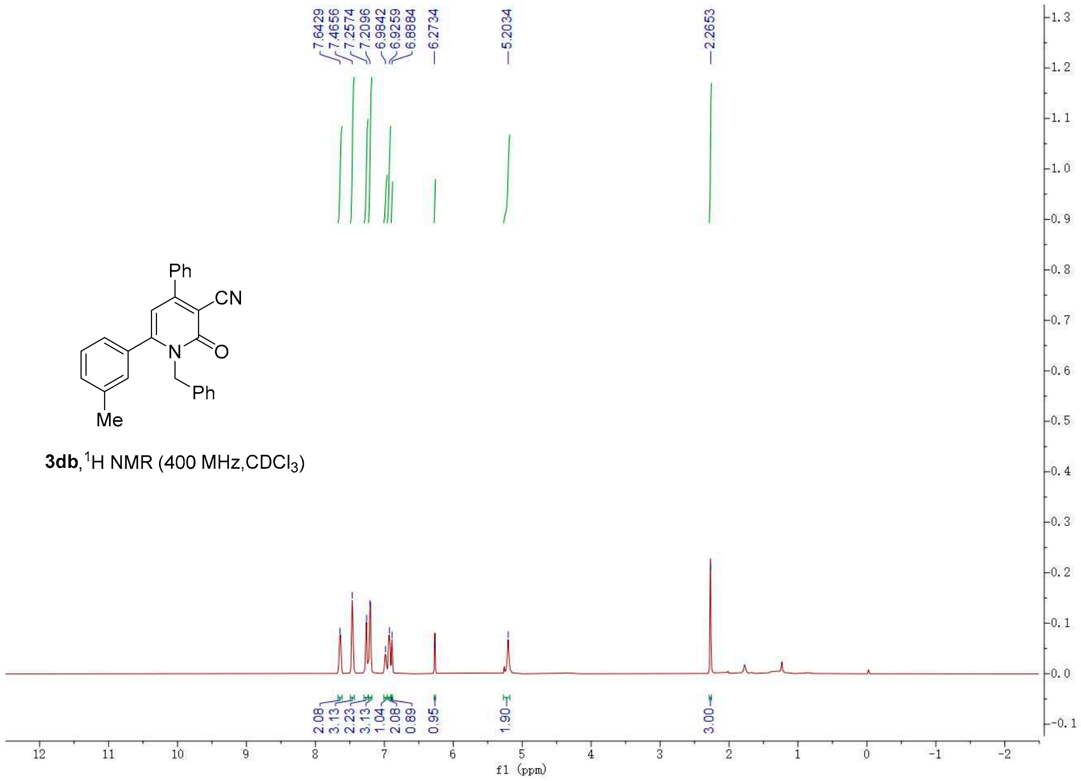

3db, ^{1}H NMR (400 MHz,CDCl$_3$)

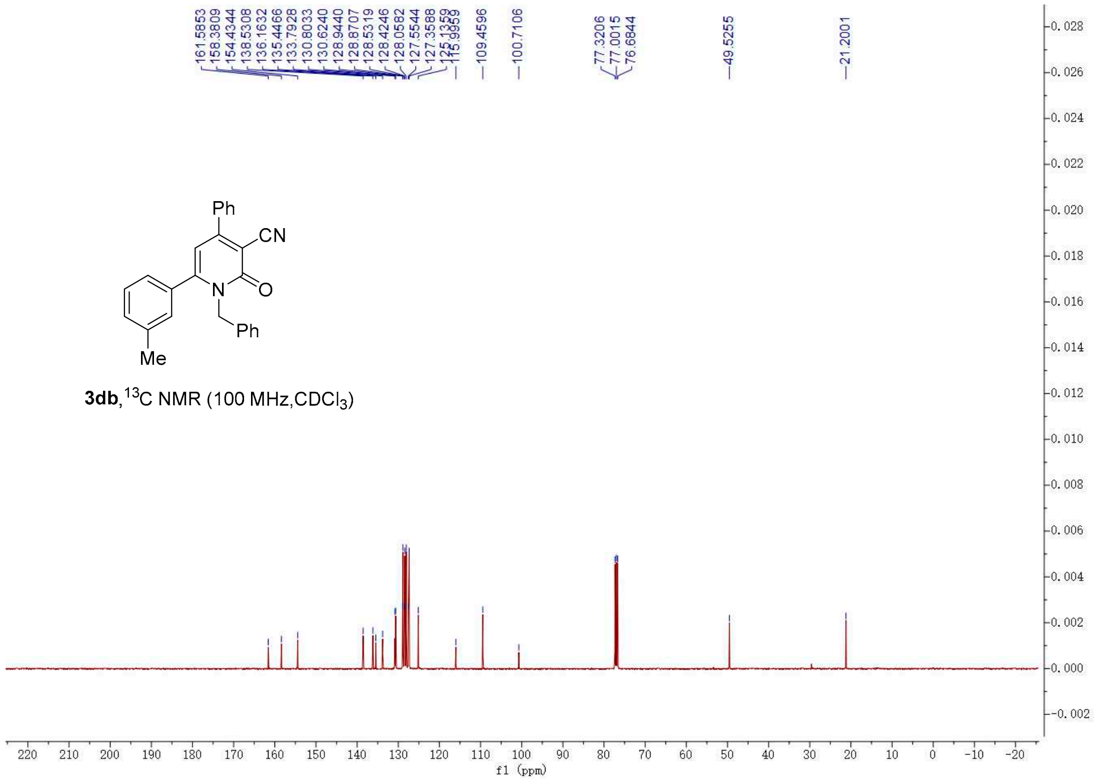

3db, ^{13}C NMR (100 MHz,CDCl$_3$)

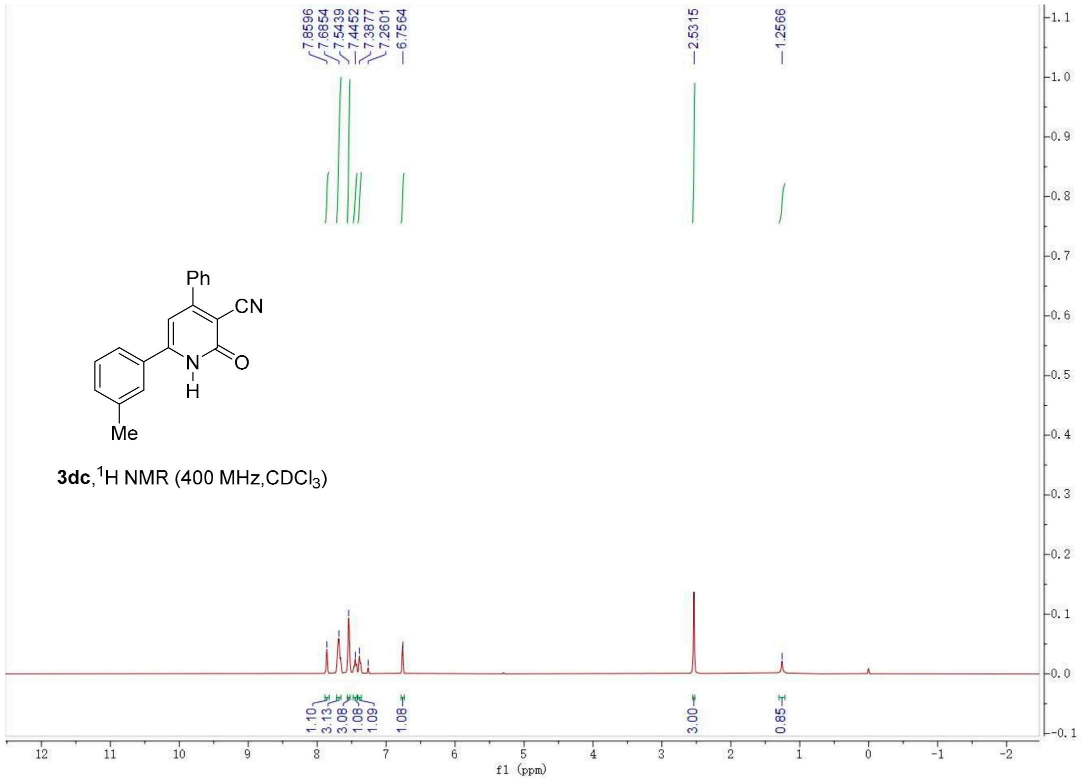
7.8596
7.6854
7.5439
7.4452
7.3877
7.2601
6.7564
2.5315
1.2566
Ph
CN
N
H
O
Me
3dc, 1H NMR (400 MHz,CDCl3)
1.10
3.13
3.08
1.08
1.09
1.08
3.00
0.85
f1 (ppm)

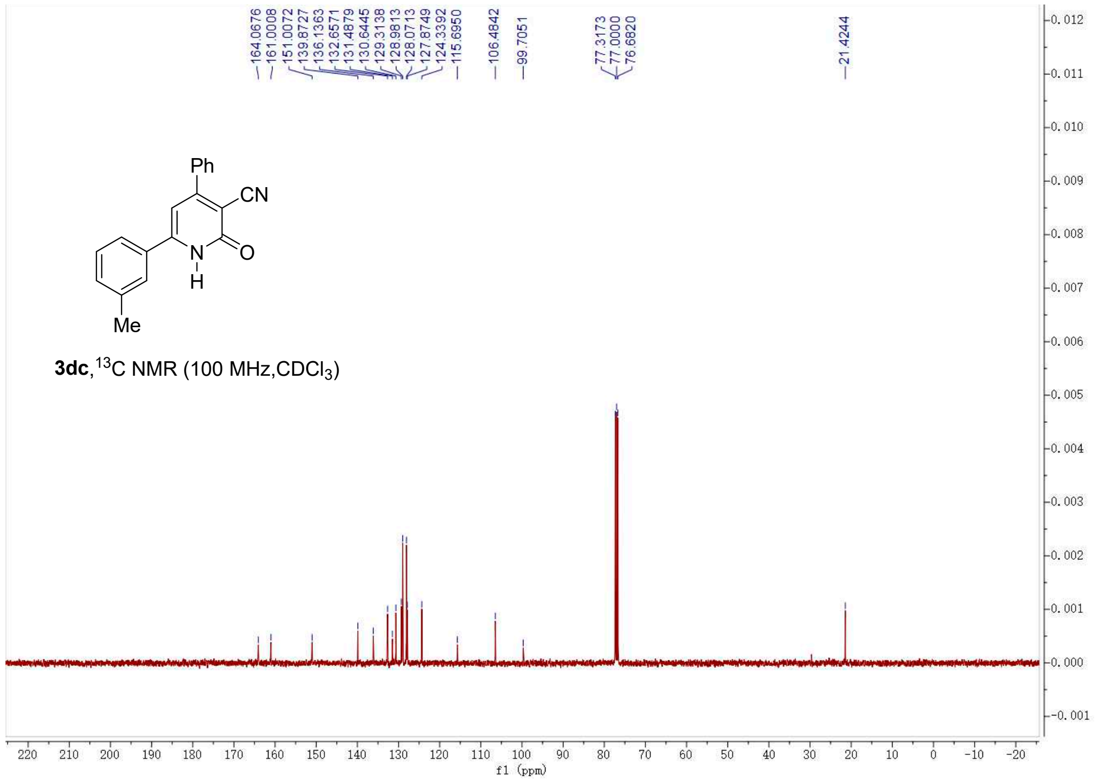
164.0676
161.0008
151.0072
139.8727
136.1363
132.6571
131.4879
130.6445
129.3138
128.9813
128.0713
127.8749
124.3392
115.6950
106.4842
99.7051
77.3173
77.0000
76.6820
21.4244
Ph
CN
N
H
O
Me
3dc, 13C NMR (100 MHz,CDCl3)
f1 (ppm)

Insecticidal Properties Study Methods:

This insecticidal activity test was conducted using the drug membrane method. Their main principle is to place the test insect on a drug membrane, causing it to crawl, come into contact with and die from poisoning, in order to determine the biological toxicity of the insecticide. This method often involves soaking, applying, spraying, doping and feeding insecticides to the surface of an object in a specific amount (or concentration) to form a dispersed and uniform drug membrane, allowing the insect to come into contact (or nibble) with the drug membrane, thereby producing a toxic effect.

This experiment involves evenly applying different concentrations of the tested compound solution onto clean and pollution-free bread, with a dual effect of feeding and drug contact, in order to achieve insecticidal effects. After applying the medicine, it will be left to stand for 1 hour. Then we put 30 equally sized and healthy second instar larvae of yellow mealworms into a 1000mL beaker.

We recorded the time of administration of yellow mealworms at the time of injection, and then observed the overall feeding and mortality of yellow mealworms at 6 time periods including 12h, 24h, 36h, 48h, 72h, 96h, and recorded the mortality and survival rates.

This article mainly focuses on the overall mortality rate of yellow mealworms after 96 hours at different drug concentrations, and then analyzes and processes the data using the SPSS Statistics27 software system and the Graphd Pism9.5 software system to obtain the LC_{50} value and toxicity regression equation of the tested ricinine derivatives.

1. Experimental materials

(1) Test reagents and solvents

Positive drug ricinine, provided by Nanjing Bailing Chemical Co., Ltd; Trichloromethane, Guangzhou Chemical Reagent Factory; Hot water (85°C~100°C), obtained by boiling laboratory tap water; and 48 ricinine derivatives synthesized in previous experiments.

(2) The source of the test insects

Second instar larvae of yellow mealworm, provided by Shanxi Dingxin Biotechnology Co., Ltd.

(3) Feed sources

Original bread, provided by Shandong Naisi Pet Products Co., Ltd.

(4) Operating experimental conditions

The optimal temperature control in the active laboratory is 25°C, maintaining a well ventilated, well lit, and dry indoor environment.

(5) Other precautions

a. The selected second instar larvae of yellow mealworms should be healthy, uniform in body shape, and of consistent size. Before conducting the activity experiment, yellow mealworms should be fed for 3 days in advance. When feeding, we only give bread and do not provide other

exogenous foods and nutrients.

b. Each group of experimental feed should be evenly distributed.

c. The number of test insects in each group should be equal.

d. Death criteria for the tested insect: The insect's body is withered and turns black, with its feet facing upwards, and there is no autonomous response when touched with a wooden stick, indicating a state of death.

e. Standard for testing live insects: The insect body is fresh, soft and moist, with a clear sense of autonomous avoidance, and it can eat normally, indicating a live insect state.

f. Mortality rate calculation.

Mortality rate (%)=(number of dead test worms ÷ number of test worms administered before administration) x 100%.

g. Survival rate calculation.

Survival rate (%)=(number of surviving test worms ÷ number of test worms administered before administration) x 100%.

h. Corrected mortality rate calculation.

Corrected mortality rate (%)={(treatment group test insect mortality rate - control group test insect mortality rate)/(1-control group test insect mortality rate)} x 100%.

2. The LC_{50} value of ricinine determination.

(1) We weighed ricinine in batches, labeled as No.1 (1mg), No.2 (2mg), No.3 (4mg), No.4 (6mg), No.5 (8mg), and No.6 (10mg) in 10mL ep tubes. We added 1mL of hot water (85°C~100°C) separately and let it stand for 3-5 minutes to completely dissolve into a transparent solution. The concentration of the obtained solution will be sequentially labeled as No.1 (1mg/mL), No.2 (2mg/mL), No.3 (4mg/mL), No.4 (6mg/mL), No.5 (8mg/mL), No.6 (10mg/mL), and No.7 will be set as a blank control group.

(2) We use an analytical balance to weigh 7 equal parts of 100g bread, and add 100g of bread to the 6 batches of different concentrations of medicine and blank control group in sequence, mixing evenly.

(3) In 7 beakers, 30 second instar larvae of yellow mealworms were added sequentially according to their serial numbers, and the administration time was recorded. The overall feeding and mortality of yellow mealworms were observed at 6 time periods, including 12h, 24h, 36h, 48h, 72h, and 96h, respectively. The mortality and survival rates were recorded.

(4) Finally, we need to calculate the mortality rate for 96 hours at different drug concentrations and correct the mortality rate to obtain the corresponding toxicity regression equation and LC_{50} value.

Table S1. Mortality rate of positive drugs at different concentrations

drug level (mg/mL)	Time (h)	death rate (%)
1	96h	6.67
2	96h	23.30
4	96h	50.0
6	96h	76.7
8	96h	90.0
10	96h	100.0
Clean water control group	96h	3.33

We can obtain a univariate linear toxicity regression equation for the positive drug ricin by analyzing the mortality rate of different positive drug concentrations for 96 hours and after blank correction: Y=10.73x+2.008, r=0.987, LC_{50} value is 3.630mg/mL.

3. The LC_{50} value of ricinine derivatives determination

Taking ricinine positive group as reference, the ricinine derivatives to be tested were dissolved in 95% ethanol, and the concentrations were set as 1 mg/mL, 2 mg/mL, 4 mg/mL, 6 mg/mL, 8 mg/mL, 10 mg/mL and blank control group (95% ethanol), and the mortality and survival rate were calculated after 96 hours, and the LC_{50} value was obtained. The toxicity of ricinine derivatives was obtained.

Table S2. The toxic effect of **3aa-3ck** on yellow mealworm

Chemical compound	Treatment time/h	Regression equation	Correlation coefficient (r)	LC_{50}/ (mg/mL)	95% Confidence limit (mg/mL)
**3aa**	96 h	Y=7.379x+2.551	0.9932	5.877	4.630~7.455
**3ab**	96 h	Y=7.924x+8.239	0.9785	4.067	3.668~4.489
**3ac**	96 h	Y=7.006x+11.518	0.9610	4.148	3.339~5.091
**3ad**	96 h	Y=6.533x+2.020	0.9944	6.910	6.519~7.347
**3ae**	96 h	Y=7.462x+9.797	0.9779	4.203	3.043~5.591
**3af**	96 h	Y=5.560x+10.19	0.9498	6.496	5.675~7.587
**3ag**	96 h	Y=7.000x+7.100	0.9805	5.175	4.225~6.344
**3ah**	96 h	Y=6.331x+5.290	0.9807	6.543	5.284~8.487
**3ai**	96 h	Y=6.388x+2.176	0.9954	7.111	6.743~7.522
**3aj**	96 h	Y=7.460x+6.491	0.9800	4.972	3.266~7.413

**3ak**	96 h	Y=7.190x+5.303	0.9856	5.475	4.004~7.562

Table S2. The toxic effect of **3aa-3ck** on yellow mealworm

Chemical compound	Treatment time/h	Regression equation	Correlation coefficient (r)	LC_{50}/ (mg/mL)	95% Confidence limit (mg/mL)
**3al**	96 h	Y=6.210x+9.169	0.9684	5.749	4.458~7.725
**3am**	96 h	Y=7.290x+5.187	0.9863	5.360	4.084~7.014
**3an**	96 h	Y=6.144x+4.693	0.9873	7.019	5.889~8.729
**3ao**	96 h	Y=7.823x+2.827	0.9933	5.401	4.101~6.930
**3ap**	96 h	Y=7.240x+6.992	0.9798	5.062	3.461~7.365
**3aq**	96 h	Y=7.917x+3.982	0.9905	5.090	3.661~6.791
**3ar**	96 h	Y=6.719x+1.688	0.9946	6.846	5.685~8.529
**3as**	96 h	Y=7.444x+6.093	0.9855	4.974	3.923~6.230
**3at**	96 h	Y=7.021x+5.094	0.9933	5.718	4.628~7.096
**3au**	96 h	Y=9.347x+8.177	0.9748	3.245	2.663~3.891
**3av**	96 h	Y=5.161x+4.286	0.9894	9.416	7.612~13.55
**3aw**	96 h	Y=6.091x+1.309	0.9959	7.844	6.641~9.882
**3ax**	96 h	Y=6.567x+7.576	0.9750	5.595	4.527~7.033
**3ba**	96 h	Y=8.140x+3.477	0.9790	4.735	4.184~5.328
**3bb**	96 h	Y=6.810x+0.280	0.9721	6.630	4.527-9.093
**3bc**	96 h	Y=7.520x+12.40	0.9610	3.579	2.990~4.225
**3bd**	96 h	Y=7.567x+6.487	0.9878	4.748	4.067~5.500
**3be**	96 h	Y=6.535x+7.402	0.9838	5.718	4.837~6.831
**3bf**	96 h	Y=6.865x+11.51	0.9664	4.317	3.372~5.458
**3bg**	96 h	Y=7.080x+6.762	0.9810	5.348	3.670~7.887
**3bh**	96 h	Y=6.636x+7.769	0.9803	5.614	3.973~8.383
**3bi**	96 h	Y=7.317x+6.654	0.9906	5.036	3.854~6.497
**3bj**	96 h	Y=7.061x+3.975	0.9840	5.923	4.380~8.281
**3bk**	96 h	Y=5.886x+6.319	0.9809	7.184	5.500~10.78
**3bl**	96 h	Y=6.354x+5.662	0.9860	6.366	5.704~7.170
**3bm**	96 h	Y=5.615x+8.476	0.9707	7.037	5.835~8.951
**3ca**	96 h	Y=7.492x+2.048	0.9950	5.711	5.212~6.251
**3cb**	96 h	Y=6.877x+4.289	0.9926	6.092	4.808~7.906
**3cc**	96 h	Y=6.659x+5.741	0.9862	6.048	4.643~8.207
**3cd**	96 h	Y=7.082x+11.02	0.9701	4.249	3.030~5.785
**3ce**	96 h	Y=6.825x+5.961	0.9890	5.719	4.594~7.217
**3cf**	96 h	Y=9.044x+3.293	0.9885	4.421	2.705~6.357
**3cg**	96 h	Y=8.026x+8.272	0.9820	4.067	2.990~5.302
**3ch**	96 h	Y=7.379x+9.234	0.9792	4.334	3.769~4.951
**3ci**	96 h	Y=8.810x+19.56	0.9502	2.206	1.225~3.343
**3cj**	96 h	Y=7.819x+11.56	0.9520	3.483	2.928~4.093
**3ck**	96 h	Y=6.957x+7.290	0.9825	5.204	4.455~6.071
**3da**	96 h	Y=5.874x+7.789	0.9762	6.672	5.771~7.900
**3db**	96 h	Y=4.657x+12.72	0.8953	7.995	6.352~11.13
**3dc**	96 h	Y=6.800x+11.33	0.9651	4.451	3.183~6.132
**Ricinine**	96 h	Y=10.73x+2.008	0.9870	3.630	3.063~4.234
